# Effects of Diet, Lifestyle, Chrononutrition and Alternative Dietary Interventions on Postprandial Glycemia and Insulin Resistance

**DOI:** 10.3390/nu14040823

**Published:** 2022-02-16

**Authors:** Emilia Papakonstantinou, Christina Oikonomou, George Nychas, George D. Dimitriadis

**Affiliations:** 1 Laboratory of Dietetics and Quality of Life, Department of Food Science and Human Nutrition, Agricultural University of Athens, 11855 Athens, Greece; christina.oikonomou12@gmail.com; 2 Laboratory of Microbiology and Biotechnology of Foods, Agricultural University of Athens, 11855 Athens, Greece; george.nychas@gmail.com; 3 Sector of Medicine, Medical School, National and Kapodistrian University of Athens, 15772 Athens, Greece; gdimitr@med.uoa.gr

**Keywords:** postprandial hyperglycemia, insulin resistance, insulin secretion, chrononutrition, dietary interventions, exercise

## Abstract

As years progress, we are found more often in a postprandial than a postabsorptive state. Chrononutrition is an integral part of metabolism, pancreatic function, and hormone secretion. Eating most calories and carbohydrates at lunch time and early afternoon, avoiding late evening dinner, and keeping consistent number of daily meals and relative times of eating occasions seem to play a pivotal role for postprandial glycemia and insulin sensitivity. Sequence of meals and nutrients also play a significant role, as foods of low density such as vegetables, salads, or soups consumed first, followed by protein and then by starchy foods lead to ameliorated glycemic and insulin responses. There are several dietary schemes available, such as intermittent fasting regimes, which may improve glycemic and insulin responses. Weight loss is important for the treatment of insulin resistance, and it can be achieved by many approaches, such as low-fat, low-carbohydrate, Mediterranean-style diets, etc. Lifestyle interventions with small weight loss (7–10%), 150 min of weekly moderate intensity exercise and behavioral therapy approach can be highly effective in preventing and treating type 2 diabetes. Similarly, decreasing carbohydrates in meals also improves significantly glycemic and insulin responses, but the extent of this reduction should be individualized, patient-centered, and monitored. Alternative foods or ingredients, such as vinegar, yogurt, whey protein, peanuts and tree nuts should also be considered in ameliorating postprandial hyperglycemia and insulin resistance. This review aims to describe the available evidence about the effects of diet, chrononutrition, alternative dietary interventions and exercise on postprandial glycemia and insulin resistance.

## 1. Introduction

“Let food be thy medicine, and let medicine be thy food”, the famous quote by Greek physician Hippocrates stands well the test of time. Diet is considered the cornerstone of prevention and treatment of glucose dysmetabolism and insulin resistance (IR), and food has major effects on postprandial glycemia and overall physical health [1]. Our modern lives are characterized by being more often in a postprandial state, decreased energy expenditure, exposure to a “toxic” food environment, sedentary lifestyle with prolonged sitting time, high consumption of energy dense foods, irregular eating occasions and times of eating, skipping meals, chronic psychological stress, emotional eating, and food consumption late at night [1]. This way of life triggers mechanisms, such as development of insulin resistance (IR), proposed as a defense system against metabolic stress, especially for the heart [2].

**Figure 1:**
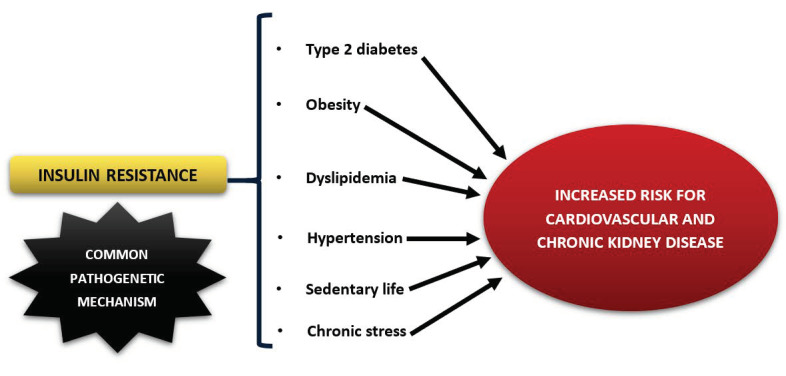
Risk factors for cardiovascular/kidney disease in type 2 diabetes that can be modified by lifestyle interventions.

Factors associated with IR include obesity, particularly abdominal obesity, increased waist circumference, familial history of type 2 diabetes (T2DM), sedentary lifestyle, hypertension, and fatty liver [3,4,5,6] (Figure 1). Obesity pathogenesis involves two related but distinct processes: a sustained positive energy balance and resetting of the body’s weight “set point” at an increased level [7]. Obesity is a heterogeneous phenotype and not every obese person is at risk for developing metabolic abnormalities; therefore, one needs to focus on the individual [8,9]. It has been reported that although there is a causal role of hyperinsulinemia in the progression of obesity with a well-established connection, its contribution varies [9]. The amount of visceral adipose tissue, estimated by increased waist circumference, may reflect the severity of IR [10]. IR is also characterized by hypertriglyceridemia, lower concentrations of high-density lipoprotein (HDL) cholesterol in blood, and increased inflammation [11]. As a result, glucose metabolism deteriorates, resulting in hyperglycemia, impaired glucose tolerance (IGT) or overt T2DM. Weight loss and regular moderate intensity physical activity/exercise are significant factors for IR prevention and/or treatment [12]. From a dietary perspective, dietary fiber, cereal fiber, fruit fiber, whole grains, full-fat dairy products [13,14,15,16], magnesium, and calcium lower IR, whereas high glycemic index (GI) and glycemic load (GL) foods, saturated fat, salt (deficiency or excess), and alcohol (>30 g/day) increase IR [17].

T2DM development stems from various genetic and/or environmental factors and is characterized by deficient pancreatic β-cell insulin secretion and decreased sensitivity/responsiveness of insulin-sensitive tissues to insulin [18]. The prevailing view in the physical history of T2DM is that IR precedes causing a progressive increase in insulin secretion to compensate for IR and maintain glucose tolerance (IGT-prediabetes). In the long term β-cell function declines, and hyperglycemia follows (overt T2DM) [19]. However, in some individuals, hyperinsulinemia may not be a compensatory response to insulin resistance but rather a primary defect due to hypersensitivity of β-cells explained by genetic/environmental factors, gastrointestinal/neural signals, or various substrates such as lactate, non-esterified fatty acids (NEFA)/triglycerides, or amino acids [20,21,22,23].

Uncontrolled T2DM may lead to serious consequences on health and health care cost [24,25] and its prevalence is high with over 427 million adults being affected worldwide expected to reach 629 million people by 2045 [25,26,27]. T2DM is related to micro- and macrovascular chronic complications, such as retinopathy, neuropathy, chronic kidney disease, cardiovascular disease (CVD), and non-alcoholic fatty liver disease (NAFLD) [25,27,28,29].

The current COVID-19 pandemic has led to dramatic societal changes leading to changes in consumers’ food practices with fewer cooking-related practices, bulk buying, increased fruit, vegetables and saturated fat intake, and increased body weight [30], all of which may have a significant effect on postprandial glycemia and IR.

### Mechanisms for the Regulation of Postprandial Hyperglycemia

During meal ingestion, several mechanisms operate in concert (gastric emptying/intestinal glucose absorption, secretion/action of gastrointestinal hormones, changes in insulin/glucagon secretion/action, hyperglycemia mass action) to ensure optimal regulation of postprandial glucose fluctuations in the bloodstream via coordination by the central nervous system (CNS) (reviewed in detail in reference [31]): (a) After a meal, insulin plays a primary role in the regulation of glucose homeostasis through its effect on insulin-sensitive tissues (liver/skeletal muscle/adipose tissue) [32]. In the beginning of meal ingestion, there is a rapid increase in the secretion of insulin and a parallel decrease in that of glucagon from pancreatic β- and α-cells, respectively. These hormonal changes, rapidly inhibit hepatic glucose production (HGP) to allow incorporation of more than 30% of the ingested glucose into liver glycogen (direct pathway of glycogen synthesis) [33,34]. The preferential use of the liver to dispose a significant amount of the ingested glucose during first pass, along with a 60–70% hepatic clearance of insulin, protect the peripheral circulation from excessive hyperglycemia/hyperinsulinemia in the postprandial period [35,36,37,38,39,40,41,42,43]. The entrance of the remaining insulin into the peripheral circulation rapidly suppresses lipolysis in the adipose tissue and reduces plasma levels of NEFA; this facilitates the decrease of HGP/increase of glucose storage by insulin and permits the increase of insulin-stimulated glucose uptake by skeletal muscle for oxidation and storage as glycogen [32,44]. However, if the liver is overpowered by increased amounts of carbohydrate in the meal, the escape of glucose to the peripheral circulation will be much higher engaging skeletal muscle for its removal; this will require a substantial increase in insulin secretion and plasma insulin levels which, in the long term, may lead to the development/aggravation of IR [45,46,47]. If circulating glucose exceeds the capacity of skeletal muscle for oxidation/storage, muscle cells convert glucose to lactate/alanine/glutamine which are then delivered back to the liver and incorporate their carbons to glycogen (indirect pathway of glycogen synthesis) [34,48,49]. Considering both the direct (first pass) and indirect pathways (through muscle) of glycogen synthesis, overall, the liver disposes more than 50% of the ingested glucose and hence allows a much smaller amount to remain in the peripheral circulation [50]. Therefore, as suggested by Kowalski et al. [51] the liver may represent an “evolutionary conserved mechanism” for the regulation of postprandial hyperglycemia with a mission to reduce the secretory burden on β-cells, thus preventing plasma glucose and insulin levels from rising too high and induce serious consequences [37,38,40,41,43]. Insulin also affects the vascular endothelium and increases the rates of blood flow in skeletal muscle and adipose tissue. After meals, the increase of blood flow in muscle facilitates the delivery of substrates/hormones for metabolism, whereas in adipose tissue it ensures the clearance of NEFA from the circulation [52,53]. In conditions such as IGT, T2DM or obesity, insulin-stimulated rates of blood flow in adipose tissue and muscle are severely impaired [54,55,56]. (b) The role of insulin sensitivity in postprandial glucose regulation is important to restrain hyperinsulinemia after meals. The amount of insulin release from the β-cells under all circumstances depends on the metabolic requirements of the insulin-sensitive tissues. Therefore, if the sensitivity of these tissues to insulin is increased, the management of an increased entry of glucose into the bloodstream after meals could be achieved in the absence of marked increases in the plasma levels of insulin [57]. Indeed, Kahn et al. [58] provided experimental evidence showing that the pancreatic β-cells and insulin-sensitive tissues interact in a tightly regulated manner so that when insulin sensitivity is high, insulin secretion is low and vice versa. Therefore, lifestyle interventions, such as those described in the present review, are important to improve tissue sensitivity to insulin and hence avoid marked hyperglycemia and hyperinsulinemia after meals. (c) Glucose can regulate its own metabolism in tissues independently of insulin [59]. In the liver, hyperglycemia per se increases the rates of glucose uptake and suppresses HGP, thus facilitating glucose storage [60], whereas in muscle it increases the rates of glucose uptake and phosphorylation [61]. Therefore, as suggested by Kowalski et al. [51], the subtle increase in plasma glucose levels seen after meals in physiological conditions, may serve to collaborate with insulin to minimize the amount of insulin required for its effects on insulin-sensitive tissues and, hence, reduce the secretory burden on β-cells. (d) The gastrointestinal tract plays a significant role in the regulation of hyperglycemia during food ingestion via feedback mechanisms coordinated by the CNS [62]. The rate of gastric emptying is a major determinant of the glycemic/insulin responses following a meal and has been shown to correlate positively with postprandial hyperglycemia [63,64]. Furthermore, in response to a meal the intestinal tract secretes several peptides (incretins) that regulate its motility/absorption capacity, and insulin/glucagon secretion and action [65,66]. Glucagon-like peptide-1 (GLP-1) and glucose-dependent insulinotropic polypeptide (GIP) have been heavily investigated due to their clinical application in T2DM; they collaborate to increase glucose-dependent insulin secretion and insulin sensitivity [67,68]. The coordinated interplay between the gastrointestinal tract and pancreatic β- and α-cells ensures a finely tuned adjustment of glucose supply to the metabolic needs and, therefore, helps to avoid high glucose/insulin fluctuations after meals. (e) Meal sequence within the day ensures that preceding meals sensitize the metabolic/incretin system to the following ones, thereby improving glucose tolerance during the day mostly by increasing glucose storage in the liver but also by facilitating glucose uptake in skeletal muscle [69,70,71,72,73] (discussed in detail in Section 2.3). Finally, meal composition, and ingestion of low GI/GL foods, particularly when consumed along with protein and/or fat, reduce postprandial glucose and insulin responses, thus improving insulin sensitivity [45,46,74,75,76,77,78,79] (discussed further in Section 4.2.1).

## 2. Effects of Macronutrients in Foods and Meals on Postprandial Hyperglycemia and IR

**Figure 2:**
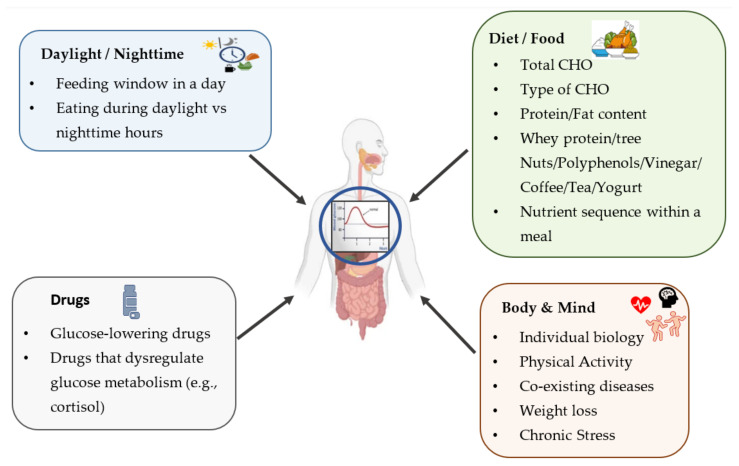
Biological and environmental factors affecting postprandial hyperglycemia and insulin resistance (abbreviations: CHO: carbohydrates).

The glycemic response depends on many factors including the amount of total consumed carbohydrates, carbohydrate type, starch type (i.e., resistant starch), food preparation (i.e., gelatinization, bran size), other macronutrients in foods (i.e., fat, protein, fiber), and the physiological organic functions (stomach, pancreatic and enteral hydrolysis, gastric emptying, intestinal absorption rate of nutrients, etc.) [78] (Figure 2). Regarding dietary components, a key dietary strategy for treating postprandial hyperglycemia, hyperinsulinemia, and IR is the consumption of foods and meals that diminish the glucose fluctuations known to induce oxidative stress and β-cell damage [80]. Indeed, increased glucose variability from peaks to nadirs has been recognized as a major metabolic defect leading to endothelial damage and CVD in T2DM [81]. The eating pace of consuming a meal has also been implicated in modulating postprandial hyperglycemia; for example, eating fast has been associated with higher glycemic excursions in healthy women [82]. Moreover, another important strategy to consider is the consumption of foods and meals that induce a lower GL and delay gastric emptying, thus leading to decreased insulin requirements and postprandial glucose excursions and may also reduce hunger and desire to eat [31]. Such foods and meals typically contain high fiber, particularly soluble fiber, low amounts of easily absorbable carbohydrates, low amounts of total carbohydrates, and are high in proteins [31,46,75].

Carbohydrates are well known to be the major macronutrients affecting postprandial glycemia. A study using continuous glucose monitoring in subjects with T2DM showed that for achieving lower 24-h glucose peaks it was better to consume at least half of the amount of carbohydrates at lunch time and avoid consuming significant amount of carbohydrates in the morning (at breakfast) or in the evening dinner [83]. A cross-sectional study examining the effects of different proportions of carbohydrates at breakfast on postprandial glucose fluctuations in IGT (*N* = 55) and normal glucose tolerance (NGT) individuals (*N* = 78), using continuous glucose monitoring, recorded breakfast meals according to the proportion of carbohydrates into low (<45%), medium (45% to 65%) and high carbohydrates (>65%), and reported a gradual increase in postprandial glucose fluctuations with increasing proportions of carbohydrates in breakfast, higher postprandial glucose excursions, higher postprandial glucose spikes, and longer time period in which glucose levels decreased to baseline in subjects with IGT compared to NGT individuals; this study concluded that in IGT a high-carbohydrate meal at breakfast should be avoided and a low carbohydrate meal should be recommended instead [84]. Carbohydrate-rich foods that may be more effective in ameliorating postprandial hyperglycemia and IR include legumes/pulses [85,86,87], whole grains [88,89,90,91], and pasta [92]. In 2017, the American Diabetes Association (ADA) proposed consuming the following foods for preventing T2DM along with weight loss: whole grain cereal products, nuts, yogurt, coffee and tea and limit consumption of red meat and sodas containing sugar [93].

In conclusion, macronutrient content of foods and meals affects significantly postprandial hyperglycemia and IR. Carbohydrates are the main macronutrients that affect glycemic responses. However, the type, total amount, other macronutrients consumed in parallel, physiological organic function, the GI/GL of carbohydrate containing foods, the extent to glucose excursions and fluctuations, and the time of day that the majority of carbohydrates is consumed, will be determinant factors of postprandial glucose and insulin increases and amelioration or not of IR.

### 2.1. Effects of Diet on Postprandial Hyperglycemia and IR

There are several dietary patterns that have been suggested as choices for prevention and/or treatment of T2DM individuals, independently of body weight status. All rigorously investigated healthy dietary patterns, i.e., Mediterranean-style diet [94,95,96], vegetarian diets [97,98,99,100], Nordic diet [101], and the Dietary Approaches to Stop Hypertension (DASH) [94,102] have been associated with a lower risk of developing T2DM. The PREDIMED trial that compared a Mediterranean-style to low-fat eating pattern for prevention of T2DM, reported a 30% lower relative risk in people of high cardiometabolic risk [103,104], whereas another trial examining a healthy Nordic diet, reported a 25% lower risk in women and 38% in men [101]. Results from the Dietary Intervention Randomized Controlled Trial (DIRECT) assigning obese adults with T2DM to a calorie-restricted Mediterranean-style, a calorie-restricted lower-fat, or a low carbohydrate eating pattern (28% of calories from carbohydrate), showed that HbA1c was lowest in the low carbohydrate group after 2 years, whereas fasting plasma glucose was lower in the Mediterranean-style group than in the lower-fat group [105]. Results from another RCT showed that despite only a 2-kg difference in weight loss, the group following a low carbohydrate Mediterranean-style eating pattern experienced greater rates of at least partial diabetes remission, with rates of 15% at year 1 and 5% at year 6 compared with 4.7% and 0%, respectively, in the group following a low-fat eating plan [106].

Results from a network meta-analysis of RCTs in T2DM adults examining 9 different dietary approaches (vegetarian, Mediterranean-style diet, high protein, moderate-carbohydrate, low carbohydrate, low-GI/GL, paleolithic, low-fat and control diet) with a duration of at least 12 weeks, showed that all dietary approaches significantly reduced Hb1Ac and fasting blood glucose levels, although the low carbohydrate and the Mediterranean-style diets were the most effective for HbA1c, and the Mediterranean-style and vegetarian diets were the most effective for fasting glucose reduction [107]. Although this network meta-analysis reported some benefits of the paleolithic diet, there was only one available study included in their analysis [107]. Moreover, this network meta-analysis reported that low carbohydrate diets were more effective in reducing HbA1c in T2DM aged ≥60 years, whereas the Mediterranean-style style, the moderate-carbohydrate, the low-GI/GL, the high protein, and the low-fat diets were more effective in HbA1c reduction in T2DM aged <60 years [107]. Compared to the 9 dietary approaches examined, the Mediterranean-style diet was a more effective dietary approach for improving postprandial hyperglycemia and IR compared to the other 8 analyzed dietary schemes [107].

### 2.2. Effects of Weight Loss on Postprandial Glycemia and IR

Weight loss has been proposed as a key strategy for the treatment of postprandial hyperglycemia, hyperinsulinemia, and IR [108]. The proposed treatment for overweight and obesity in people with prediabetes and/or overt T2DM includes firstly diet, physical activity, and behavioral therapy, then pharmacotherapy in those with body mass index (BMI) >27 kg/m^2^ and finally bariatric surgery in those with BMI >35 kg/m^2^ [109]. In the obese, insulin resistant, and T2DM individuals, the beneficial effects of weight loss on postprandial glycemia and IR are mostly due to the decreased blood levels of NEFA [110,111] and to an improvement in the insulin-mediated suppression of fat oxidation [112]. Postprandial switch from fat to carbohydrate oxidation was also observed in prediabetic subjects who had 14 kg weight loss [113]. This suggests that impairments in the regulation of substrate utilization by skeletal muscle can be reversible and contribute to improvements in metabolic health and glycemia. These changes are accompanied by improvements in insulin sensitivity and have been observed in most weight loss interventions. A recent consensus report of the ADA recommended a 7–10% of initial body weight reduction, if required, and maintenance to prevent progression from prediabetes to T2DM, and noted the potential for diabetes remission [114].

Eating plans that create an energy deficit should be customized to fit the person’s preferences, metabolic goals, and resources, to achieve long-term sustainment [114]. Regular physical activity, which can contribute to both weight loss and prevention of weight regain, and behavioral strategies are also important components of lifestyle therapy for weight management [115,116,117,118,119,120]. Structured weight loss programs with regular visits have been shown to enhance weight loss in T2DM [121,122,123]. The threshold of weight loss for maximal clinical benefits in T2DM is unknown, but it has been shown that the greater the weight loss, the greater the benefits [114]. The UK Prospective Diabetes Study (UKPDS) demonstrated that decreases in fasting blood glucose levels were correlated with the degree of weight loss [124]. A meta-analysis found that lifestyle interventions producing <5% weight loss had less effect on HbA1c, lipid profile, or blood pressure (BP) compared to studies achieving weight loss >5% [119]. Table 1 describes the effects of dietary schemes/patterns and lifestyle interventions in postprandial hyperglycemia and cardiovascular disease risk factors.

There is still a great debate as to which is the best dietary macronutrient composition for weight loss, amelioration of postprandial hyperglycemia and IR. Individualization of the macronutrient composition of the diet should depend on the health status of the individuals, their metabolic goals (glycemia, lipid profile, BP, hepatic status, renal status, etc.), physical activity/exercise levels, food preferences or aversions, and food availability [114].

#### 2.2.1. Low Calorie Diets for Weight Loss

The Look AHEAD Trial, the Diabetes Remission Clinical Trial (DiRECT) and the Diabetes Intervention Accentuating Diet and Enhancing Metabolism (DIADEM-I) highlighted the potential for T2DM remission, defined as the maintenance of euglycemia (complete remission) or prediabetes level of glycemia (partial remission) with no diabetes medication for at least 1 year, with a weight loss of 15 kg or more within one year following a low or a very low-calorie diet, and also using meal replacements [125,126,127,128] in people undergoing weight loss treatment. In the Look AHEAD trial, when compared with the control group, the intensive lifestyle arm (more visits, 175 min/week unsupervised physical activity, dietary modification counseling, and a weight loss goal of 10%) resulted in at least partial diabetes remission in 12% of participants as compared with 2% in the control group [125]. The DiRECT trial showed that at 1 year, weight loss associated with the lifestyle intervention resulted in diabetes remission in 46% of participants [121]. Remission rates were related to the magnitude of weight loss, rising progressively from 7% to 86% as weight loss for 1 year increased from <5% to >15% [121]. However, there is a disagreement on whether a low-calorie diet (800 kcal/day) is sustainable or really wanted by all IGT and T2DM patients. Moreover, an early rapid weight loss is typically followed by weight plateau and progressive regain [129], linked to weight loss induced cell stress, altered adipokine secretion, reduced lipolysis and induced inflammatory response in adipose tissue [130]. Therefore, a low-calorie diet may be used to reach adequate weight loss rapidly; however, for maintenance of the lost weight a less restrictive diet is used, typically a more balanced, lower fat diet.

#### 2.2.2. Low(er)-Fat Diets

The typical dietary scheme used in almost all weight loss studies, either as the primary diet or as the control diet is a balanced, low-fat diet, and its beneficial effects on weight loss (of about 3.2 kg greater weight loss as a result of consuming a low-fat ad libitum diet) and maintenance have been well documented: compliance is easier in the long-term, and its reported effects are more pronounced in subjects with a higher body weight [131]. The strongest evidence for T2DM prevention using a low-fat diet approach comes from several studies, including the U.S. Diabetes Prevention Program (DPP) [115,132] and the Finnish Diabetes Prevention Study [133], demonstrating that a lifestyle intervention (combination of a low-fat diet combined with at least 150 min of moderate intensity weekly exercise) led to weight loss and decreased T2DM incidence for adults with overweight/obesity and IGT by 58% over 3 years [115]. Moreover, follow-up of 3 large studies of lifestyle intervention for diabetes prevention has shown sustained reduction in the rate of conversion to T2DM: 43% reduction at 20 years in the Da Qing Diabetes Prevention Study [134]; 43% reduction at 7 years in the Finnish Diabetes Prevention Study [135] and 34% reduction at 10 years [132], and 27% reduction at 15 years extended follow-up in the DPP [136] in the U.S. Diabetes Prevention Program Outcomes Study (DPPOS). The follow-up of the Da Qing study also demonstrated a reduction in CVD and all-cause mortality [137].

Results from one meta-analysis showed a greater, but not clinically significant, body fat change (by 16 g/day) favoring lower fat diets compared to lower carbohydrate diets [138]. A systematic review in people with T2DM [139], several studies [140,141,142,143], and a meta-analysis [144] suggested that lowering total fat intake did not consistently improve glycemia or CVD risk factors in T2DM, but the benefit from a low-fat eating pattern appeared to be mostly related to weight loss and not to the eating pattern itself [145,146].

In conclusion, weight loss can be achieved with low(er)-fat diets, but this seems to be inferior to low carbohydrate diets, and remission is expected to be lower than that achieved with low carbohydrate diets.

#### 2.2.3. Low(er)-Carbohydrate Diets

The beneficial effects of lower carbohydrate diets on IR and other cardiometabolic risk factors in obese individuals are independent of weight loss [77]. There are many types of low carbohydrate diets. Each diet has varying restrictions on the types and amounts of carbohydrates consumed. However, severe carbohydrate restriction should be carefully monitored. It has been reported that short-term adoption of a ketogenic diet induces more severe hepatic IR than an obesogenic high-fat diet [147].

**Table 1:** Effect of dietary schemes/patterns and lifestyle interventions on metabolic outcomes (glycemia, lipidemia, cardiovascular disease factors) in individuals at high risk for developing or with diagnosed type 2 diabetes.

Study	Health Status Age (Years) BMI (kg/m^2^)	Duration and Design of Dietary Intervention	Sample Size	Description of Groups	Dietary Intervention	Selected Clinical Outcomes
Effect of lifestyle interventions on glycemia and other major metabolic outcomes in T2DM individuals						
Look AHEAD Research Group, 2010 [123]	T2DM 58.6 ± 6.8 35.9 ± 6.0	11 Years Randomized, controlled trial	5145	Intensive lifestyle intervention (ILI) Diabetes support and education (DSE, control group)	ILI: usual medical care combined with an intensive 4-year program designed to increase physical activity and reduce initial weight by 7% or more. DSE: usual medical care, provided by their own primary care physicians, plus three group educational sessions per year for the first 4 years	After 4 years: BW (kg) −6.15% in ILI vs −0.88% in DSE HbA1c (mmol/L) −0.36% in ILI vs 0.09% in DSE systolic BP (mmHg) −5.33 in ILI vs −2.97 in DSE diastolic BP (mmHg) −2.92 in ILI vs −2.48 in DSE HDL (mg/dL) 3.67 in ILI vs 1.97 in DSE TG (mg/dL) −25.56 in ILI vs −19.75 in DSE
Johansen, et al., 2017 [148]	T2DM 54.6 25–40	12 months Randomized, assessor-blinded, single-center study	98	Lifestyle group (LG) Standard care (SC)	LG: 5–6 weekly aerobic sessions of 30–60 min, with 2–3 sessions of resistance training and an individual dietary plan with 45–60% CHO, 15–20% PRO, and 20–35% FAT (<7% saturated fat) SC: medical counseling, lifestyle advice	HbA1c (mmol/L) 6.65–6.34% in LG 6.74–6.66% in SC Reduction in Glu-lowering medications −73.5% in LG −26.4% in SC
Linmans, et al., 2011 [149]	IGT or T2DM 62.9 ± 11.8 30.4 ± 4.9	1 year Randomized trial	2818	Intervention group (IG) Control group (CG)	IG: with lifestyle coaches supervising the program, >30 min exercise for >5 days/wk CG: usual care according to a diabetes management program	IG group: HbA1c (mmol/L) −0.12% Fasting Glu (mmol/L) −0.17 NS changes in the CG
Chee, et al., 2017 [150]	Overweight/obese, T2DM, and HbA1c 7%–11% 30–65 >23	6 months Randomized controlled clinical trial	230	Usual care (UC) Τrans-cultural diabetes nutrition algorithm-conventional counseling (tDNA-CC) Τrans-cultural diabetes nutrition algorithm-motivational interviewing (tDNA-MI)	UC: clinical care according to Malaysian Clinical Practice Guidelines for T2DM (2009) and low-calorie diet (1200 or 1500 kcal/day) tDNA-CC low-calorie meal plan (1200 or 1500 kcal/day) and a physical activity prescription for >150 min/wk with conventional counseling tDNA-MI low-calorie meal plan (1200 or 1500 kcal/day) and a physical activity prescription for >150 min/wk with motivational interviewing	tDNA-MI BW (kg) −6.9 ± 1.3 in tDNA-MI −5.3 ± 1.2 in tDNA-CC −0.8 ± 0.5 NS in UC HbA1c (mmol/L) −1.1 ± 0.1% in tDNA-MI −0.5 ± 0.1% in tDNA-CC −0.2 ± 0.1%, NS in UC Fasting plasma Glu (mmol/L) −1.1 ± 0.3 in tDNA-MI −0.6 ± 0.3, NS in tDNA-CC 0.1 ± 0.3, NS in UC Systolic BP (mm Hg) −9 ± 2 in tDNA-MI −9 ± 2 in tDNA-CC −1 ± 2, NS in UC
Effect of lifestyle interventions on glycemia and other major metabolic outcomes in individuals with impaired glucose tolerance or at high risk for T2DM						
Lindström, et al., 2006 [135]	Overweight with IGT 55 31.1	7 years Randomized controlled trial	522	Intervention group (IG) Control group (CG)	IG: <30% FAT, <10% saturated FAT, >15 g per 1000 kcal Fibers, and moderately intense physical activity 30 min per day or more CG: general health information at baseline without specific individualized advice	Incidence of T2DM 4.3 per 100 person-years (IG) vs 7.4 per100 person-years (CG) ↓43% relative risk in IG
Diabetes Prevention Program Research Group 2015 [136]	At high risk for T2DM 50.6 ± 10.7 34.0 ± 6.7	15 years Randomized controlled clinical trial	3234	Intensive lifestyle intervention (ILS) Metformin (MET) Placebo (PLBO)	ILS: low calorie and low lipid diet, plus 150 min physical activity per week MET: 850 mg x2/day PLBO: x2/day	ILS: ↓18% diabetes incidence rate MET: ↓27% diabetes incidence rate ILS: ↓8.7% aggregate microvascular prevalence in women
Ujvari, et al., 2014 [151]	18–40 PCOS and BMI >27 kg/m^2^ Healthy overweight/obese women (OB-C) PCOS and normal weight BMI 18.5–25 Healthy women and BMI 18.5–25	3 months Randomized trial	49	Overweight/obese women with PCOS (OB-PCOS) Overweight/obese controls (OB-C) Normal-weight PCOS (NW-PCOS) Healthy normal-weight controls (NW-C)	OB: PCOS-dietary restriction diet high in PRO and low in CHO (40% CHO, 30% FAT, and 30% PRO), and activity for 45 min 2–3 times/wk	BW (kg) −4.7 Ins (uU/mL) −11.9 Relative mRNA levels IRS1 +0.28 GLUT1 +0.06
O’ Brien, et al., 2017 [152]	IGT 45.1 ± 12.5 33.3 ± 6.5	12 months Randomized, pilot study	96	Intensive lifestyle intervention (ILI) Metformin (MET) Standard care (SC)	ILI: weight loss (5–7% of initial body weight) by improving dietary patterns (decreasing fat and calories) and promoting moderate physical activity (≥150 min per week) MET: 850 mg of metformin x2/day SC: medical care and educational materials on diabetes prevention from the National Diabetes Education Program	BW (kg) −4.0 in ILI −0.9 in MET +0.8 in SC Waist circumference (cm) −4 in ILI −1.8 in MET −0.2 in SC
Slentz, et al., 2016 [153]	Overweight/obese) 45–75 25–35	6 months Randomized, parallel clinical trial	150	High amount/moderate intensity physical activity (1) High amount/vigorous intensity physical activity (2) Low amount/moderate intensity physical activity (3) Lifestyle intervention with low amount/moderate intensity physical activity + diet (4)	(1) High amount—(67 KKW)/moderate intensity: equivalent of expending 67 KKW (~22.3 km (13.8 miles) per week) with moderate-intensity exercise (2) High amount (67KKW)/vigorous intensity—equivalent to group 2, but with vigorous-intensity exercise (75% peak VO_2reserve_) (3) Low amount—(42 kJ kg body weight^−1^ week^−1^ (KKW)//moderate intensity: equivalent of expending 42 KKW (e.g., walking ~16 km (8.6 miles) per week) with moderate-intensity (50% peak VO_2reserve_) exercise (4) diet + 42 KKW moderate intensity same as group 1 but with diet and weight loss (7%) to mimic the first 6 months of the DPP.	BW (kg) −1.94 in (1) −1.67 in (2) NS in (3) −6.44 in (4) Fat mass (kg) −2.2 in (1) −2.3 in (2) NS in (3) −6.0 in (4) AUC_Glu_ (mmol/L × 120 min) −73 in (1) −22 in (2) NS in (3) −96 in (4) AUC_Ins_ (pmol/L × 2 h) −264 in (1) −246 in (2) −166 in (3) −348 in (4)
Mensink, et al., 2003 [154]	IGT 55.6 ± 0.9 29.8 ± 0.5	2 years Randomized trial	114	lifestyle intervention group (INT) Control group (CON)	INT <35% FAT, <10% saturated FAT, >3 g/MJ fibers, physical activity for at least 1 h/wk CON usual care according to a diabetes management program	INT: BW (kg) −2.4 ± 0.7 Body fat (%) −1.0 ± 0.3 Waist (cm) −1.9 ± 0.7 Fast Glu (Mm) +0.2 ± 0.1 2 h Glu (Mm) −0.6 ± 0.3 Fast Ins (Mm/L) −1.8 ± 1.7 HOMA −0.5 ± 0.5
Roumen, et al., 2008 [155]	IGT 54.2 ± 5.8 29.6 ± 3.8	3 years Randomized controlled lifestyle intervention	106	Intervention group (INT) Control group (CON)	INT: Dietary recommendations as per Dutch guidelines for a healthy diet, and physical activity of at least 30 min a day for at least 5 days a week CON: usual care according to a diabetes management program	After 3 years in INT: BW (kg) −1.08 ± 4.30 vs +0.16 ± 4.91 CON BMI (kg/m^2^) −0.36 ± 1.47 BFM (Kg) −1.16 ± 3.80 Fasting Glu (Mm) +0.2 ± 0.1 2 h Glu (Mm) −0.05 ± 2.02 Fasting Ins (Mu/L) −1.17 HOMA −0.19

Abbreviations: IGT: impaired glucose tolerance; T2DM: type 2 diabetes; Glu: Glucose; Ins: Insulin; AUC: Area Under the Curve; CHO: carbohydrates; PRO: proteins; FAT: fats; NS: no statistically significant difference; BW: body weight; HbA1c: glycated haemoglobin A1c; BP: blood pressure; HDL: high density lipoprotein cholesterol; TG: triglycerides; BMI: body mass. index. An arrow pointing downwards indicates a decrease.

One study reported that 93% of subjects with prediabetes attained a normal HbA1c with a lower carbohydrate diet [156]. Results from a meta-analysis of 38 studies assessing a total of 6499 adults aiming to address the debate between low-fat and low carbohydrate diets and compare their effects on adiposity and lipid profiles, reported that at 6–12 months, the low carbohydrate diets were effective in improving weight loss, HDL-cholesterol and triglycerides, whereas low-fat diets were effective in improving LDL-cholesterol and total cholesterol, raising concerns about long-term adoption of low carbohydrate on potentially increasing CVD risk [157]. Another meta-analysis of RCTs [158] compared a low carbohydrate eating pattern (defined as <40% of calories from carbohydrate) to a low-fat eating pattern (defined as <30% of calories from fat). In trials up to 6 months long, the low carbohydrate eating pattern improved HbA1c more, and in trials of varying lengths, lowered triglycerides, raised HDL cholesterol, lowered BP, and resulted in greater reductions in diabetes medications [158]. Another meta-analysis of RCTs comparing low carbohydrate eating patterns (defined as <45% of calories from carbohydrate) to high carbohydrate eating patterns (defined as >45% of calories from carbohydrate) found that HbA1c benefits were more pronounced in the very low carbohydrate interventions (where <26% of calories came from carbohydrate) at 3 and 6 months, but not at 12 and 24 months [159], suggesting that severe carbohydrate restriction may not be health effective in the long-term, possibly due to diet compliance difficulties after 6 months of adopting a very low carbohydrate diet. Studies investigating severe carbohydrate restricted diets in T2DM, such as the paleolithic diet, are small and few, ranging from 13–29 participants, lasting no longer than 3 months, and have reported mixed effects on HbA1c, body weight and blood lipids [160,161,162]. A recent meta-analysis of 23 trials with 1357 participants examining the efficacy and safety of low carbohydrate diets and very low carbohydrate diets in T2DM, reported that at 6 months, compared with control diets, low carbohydrates diets achieved higher rates of diabetes remission (57% vs 31%; defined as HbA1c <6.5%), but data on remission at 12 months were sparse, ranging from a small effect to a trivial increased risk of T2DM [163]. The authors of this meta-analysis reported large clinically important improvements for weight loss, triglycerides, and insulin sensitivity at 6 months, which was diminished at 12 months [163]. Very low carbohydrate diets were found to be less effective than less restrictive low carbohydrate diets for weight loss at 6 months, explained by low dietary adherence [163]. Moreover, participants were found to have deteriorated quality of life and LDL-cholesterol at 12 months [163]. Finally, in another meta-analysis comparing low carbohydrate to high carbohydrate eating patterns, the larger the carbohydrate restriction, the greater the reduction in HbA1c, though HbA1c was similar at duration of 1 year and longer for both eating patterns [164]. However, it is questionable whether a low carbohydrate diet is sustainable long-term. In addition, low carbohydrate diets need to be higher in plant protein sources than animal protein sources to avoid unwanted elevations of plasma lipids. Both low carbohydrate diets and low-calorie diets require supplemental micronutrients (as do regular vegetarian and vegan diets), although it is unclear to what extent micronutrients can be provisioned by animal protein sources without unwanted increases in plasma lipid levels.

Overall findings tend to support evidence from existing RCTs and observational studies showing that people with markers indicating higher risk for diabetes, prediabetes or IR have lower risk when they reduce calorie, carbohydrate, or saturated fat intake and/or increase fiber or protein intake (lean animal protein or plant protein) compared with their peers [114]. For purposes of weight loss, the ability to sustain and maintain an eating plan that results in an energy deficit, irrespective of macronutrient composition or eating pattern, is very important for success [165,166,167,168]. Studies investigating specific weight loss eating plans using a broad range of macronutrient composition in people with T2DM have produced mixed results regarding efficiency and efficacy on body weight, HbA1c, lipid profiles, and BP [140,141,144,169,170,171,172,173,174,175,176]. As a result, the evidence does not identify one eating plan or a certain macronutrient composition that is clearly superior to other [177] and can be generally recommended for weight loss for people with T2DM [178]. Thus, an individualized plan is warranted, taking into consideration dietary preferences along with food preferences, metabolic goals, and ability to comply to and maintain the eating plan [179]. In each case (low-calorie or low fat or low carbohydrate diets), a Mediterranean-style diet is likely one of the best diet options after reaching weight loss goals, with intermittent re-establishment of optimal weight by reverting to either the low-calorie diet or to the low carbohydrate diet [95]. A partial reason for suggesting the Mediterranean-style diet as the maintenance diet after reaching weight loss goals is that it is a low GL diet, which supports a lower body weight.

In conclusion, weight loss is the best remedy for ameliorating postprandial hyperglycemia and IR. However, it is unclear whether weight gain and loss per se are the primary or intermediate cause relevant to T2DM or its remission. Weight loss should be rapid or steady, and the choice of dietary pattern and/or dietary macronutrient composition, should be individualized, patient-centered, and depend entirely on the person’s lifestyle, habits, eating practices, preferences, and metabolic goals.

### 2.3. Effects of Nutrient and Meal Sequence on Postprandial Glycemia and IR 

**Figure 3:**
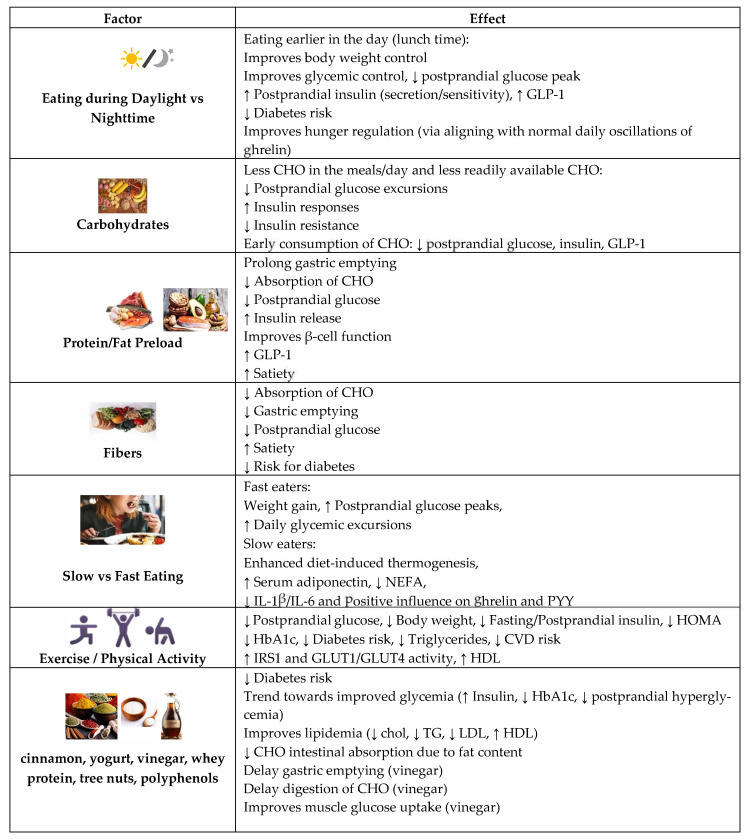
Overall metabolic effects of diet, nutrients, timing of eating occasions, speed of eating, physical activity, and alternative dietary interventions on postprandial hyperglycemia and insulin resistance. Abbreviations: Glu: glucose; GLP-1: glucagon-like peptide 1; CHO: carbohydrates; IL: interleukin; PYY: peptide YY; GLUT: glucose transporter; ppd: postprandial; BW: body weight; Ins: insulin; HOMA: homeostatic model assessment for insulin resistance; HbA1c: glycated hemoglobin A1c; IRS: insulin receptor substrate; TG: triglycerides; HDL: high density lipoprotein cholesterol; chol: cholesterol; LDL: low-density lipoprotein cholesterol. An arrow pointing upwards or downwards indicates an increase or decrease.

Diurnal regulation of glucose metabolism in the postprandial and postabsorptive state have brought into light the importance of food and meal sequence. The sequence of meals plays an important role in postprandial glycemic responses; preceding meals sensitize the metabolic and incretin system to the following ones, thereby improving glucose tolerance during the day (Figure 3) [31].

During sleep at night, the gradual development of IR, due to growth hormone and cortisol surges, ensures that blood glucose levels will be maintained within normal levels until awakening, by switching from glucose to NEFA oxidation in skeletal muscle [31]. The increase in lipolysis and supply of NEFA to the liver and kidneys will also ensure stimulation of gluconeogenesis and HGP [31]. Thus, in addition to meal composition and size, the timing of macronutrient consumption during a meal seems to be a key regulator of postprandial hyperglycemia [31,74]. There is some evidence suggesting that premeal consumption of nutrients such as water, fat, protein, or fiber as “preloads” delay the rate of postprandial glucose absorption from the small intestine and attenuate insulin secretion and glucose excursions [74,180,181,182,183,184,185]. Moreover, it has been shown that the timing of carbohydrate ingestion (i.e., carbohydrate-last meal patterns) can markedly reduce postprandial glycemia by delaying gastric emptying, enhancing glucose-stimulated insulin release, and affecting insulin clearance [31,74]. One study showed that ingestion of olive oil (fat) half-hour before the consumption of a carbohydrate meal (potato) slowed the gastric emptying rate, mitigated the postprandial rises in the levels of glucose and insulin, and GLP-1 secretion was stimulated after the meal [181]. Similarly, protein preload (in many studies whey protein) has been examined in multiple clinical trials, and favorable effects on postprandial glycemia have been reported [182,186,187]. Consumption of whey protein half-hour before a carbohydrate meal, resulted in reduced postprandial hyperglycemia and increased plasma insulin levels [182]. Reduced postprandial glucose levels was also a key finding in other trials as well when protein preloads were ingested 15 min or in one study 2 h before a meal by subjects with IGT [188,189,190]. In those studies, protein preloads before meals were also associated with increased satiety, reduced hunger, and decreases in HbA1c [189,190]. The magnitude of food and meal sequence is greater in T2DM than in healthy people, being comparable and additive to current antidiabetic medications and has been shown to sustain over time, offering a simple, effective, safe, and inexpensive tool for treating postprandial hyperglycemia and IR [31,74].

A recently proposed nutrient/meal sequence for reduced postprandial glycemic responses and improved insulin sensitivity as has been illustrated in a food pyramid paradigm from bottom (highest consumption) to top (minimum consumption) may be the following: (a) low-energy dense foods containing water, such as soups, vegetables (5 colors: fresh, cooked, sprouted, fermented) and fruits should be consumed first; followed by (b) light dairy foods (milk, yogurt, cheese), fish/seafood (lightly cooked and better cooked and consumed with vegetables), poultry and eggs (boiled); followed by (c) lean red meat or soy protein (tofu, tempeh, isolate); followed by (d) whole (legumes/pulses (sprouted, cooked), whole grains (cooked, pasta, bread, porridge, cereals), and starchy vegetables [1]. In contrast, the following foods were proposed to be consumed scarcely if at all and include processed/refined grain/flour products (breads, cakes, breakfast cereals, baked goods) fried/fatty/processed carbohydrate foods (chips, other snacks), processed/refined grain/flour foods (breads, cakes, breakfast cereals, baked goods), fried/fatty foods (chips, other snacks), sweets and sugary drinks [1]. These meal sequence options have been suggested to ameliorate postprandial glucose/insulin excursions and oxidative responses by enhancing incretin (i.e., GLP-1) secretion and delaying gastric emptying [1,191]. Moreover, carbohydrate sources containing slower absorbable carbohydrates, low in GI/GL, high in fiber and protein (i.e., whole grain cereals, beans, lentils, pasta, etc.) seem to be optimal for increased satiety, and reduced energy intake [1]. Therefore, it seems that meal and food sequence may play an important role in ameliorating postprandial hyperglycemia and IR and may be included in the dietary recommendations.

## 3. Effects of Intensive Lifestyle (Diet + Exercise + Behavior Modification) Interventions on Postprandial Hyperglycemia and IR: A Focus on Exercise

Intensive lifestyle interventions include: (a) An energy reduced (500–750 kcal/day deficit), typically a low-fat diet (≤30% total calories, 1200–1500 kcal/d for women, 1500–1800 kcal/d for men), although other dietary schemes have been also used, such as low carbohydrates, Mediterranean-style diet, etc., (b) Moderate to high intensity physical activity (≥150 min/week of aerobic physical activity, i.e., brisk walking). (c) ≥14 at least 45-min sessions (individually or in groups) supervised by specialists (doctors, dietitians, psychologists, trainers, etc.). (d) Daily monitoring of food intake and physical activity, facilitated by paper diaries or applications, weekly monitoring of weight. (e) Structured curriculum of behavior change (i.e., DPP, including goal setting, problem solving, and stimulus control), regular feedback and support from a trained interventionist, in 6 months, with (f) a goal to achieve 7–10% weight loss [151,152,192,193].

Individuals who achieve short-term weight loss should be advised to attend long-term (≥1 year) comprehensive weight management programs, with at least monthly contact and ongoing monitoring of body weight, follow a reduced calorie diet and engage in high levels of physical activity (200–300 min/week) [192,193]. To achieve long-term weight loss >5% there should be a short-term (3-month) high-intensity lifestyle interventions using very low-calorie diets (≤800 kcal/day) and long-term comprehensive weight management counseling to maintain weight loss [192,193].

Behavioral therapy (using cognitive behavior techniques, goal setting, nutrition education, monitoring, and feedback exchange) by trained dieticians and specialized health care professionals is an important component and may be achieved either by meeting in person or using technology in individual or group sessions [192,193].

Technological advances (phone applications, online appointments, phone calls, meeting online platforms and social media) can offer valid alternatives and may be equally effective [24,194,195]. One study demonstrated that adherence to self-monitoring via a technological application (mobile health, mHealth, supported by mobile devices) significantly improved weight loss [196]. Preventing negative emotions and distress can be beneficial for people with obesity, prediabetes or T2DM, and IR and can enhance adherence to the suggested dietary plan and better management of food choices [197].

Lifestyle modification programs typically prescribe 150–180 min per week of moderately vigorous aerobic activity, such as brisk walking or cycling [192]. People lacking time to exercise are encouraged to engage in multiple brief bouts (i.e., 10 min) of activity throughout the day and to increase their lifestyle activity, i.e., by using stairs vs taking the elevator [192]. The combination of diet and exercise is the most effective for reducing body weight and achieving better metabolic control compared to diet or exercise alone [148,153,193]. This combination has a dual effect both on mediating energy intake and on increasing energy expenditure to achieve a negative energy balance [148,153,193]. It has been shown that weight loss alone is effective in improving insulin sensitivity, but it is more likely to improve fasting fat oxidation and mitochondrial function if combined with exercise training [111]. It has been shown that for each kg of mean weight loss, there is a mean HbA1c reduction of 0.1% in obese and overweight subjects with T2DM; and that HbA1c lowering is greater in poor glycemic controlled vs well controlled populations with the same degree of weight loss [198]. It has also been reported that chronic exercise training improves the capacity of skeletal muscle to utilize fatty acids for fuel during exercise, and may also improve fasting fat oxidation, indicating increased metabolic flexibility, thus improving fine-tuning of the balance between fatty acid uptake, oxidation and intramyocellular triacylglycerol turnover in skeletal muscle, reducing lipid intermediates, and improving insulin sensitivity [111]. The increased exercise-induced insulin sensitivity is accompanied by a reduction in the mRNA expression of acetyl-CoA carboxylase-2 and an increase in the protein levels of hydroxyacyl-CoA dehydrogenase in muscle [199]. It has been shown that the mRNA levels of both insulin receptor substrate-1 (IRS-1) and glucose transporter-1 (GLUT-1) were increased in overweight/obese women with polycystic ovary syndrome following a lifestyle intervention that included increased physical activity (prescribed aerobic activity for 45 min two or three times/week) [151], suggesting that the changes occurring at a molecular level after exercise directly affect the mechanisms involved in insulin sensitivity and glucose metabolism in muscle. One study showed that afternoon exercise (i.e., high intensity interval training/HITT; individuals cycled at 75 rpm with a load of forced rest after 1 min, repeated six times with 1 min rest in between, three sessions/week, using continuous glucose monitoring, and recorded daily food intake) was found superior to morning exercise in improving glucose levels in T2DM [200]. A recent systematic review of 20 studies with a total of 352 participants (15 studies with T2DM and 5 with healthy subjects) concluded that exercise (moderate-intensity aerobic exercise in 18 studies, HIIT in 3 studies, and resistance exercise in one) performed post-meal instead of pre-meal regardless of time of day, improved postprandial hyperglycemia [201]. Finally, the combination of weight loss and exercise improved the aerobic capacity of skeletal muscle, increased the number of mitochondria per muscle cell and improved electron chain transport activity in obese subjects [202], having a favorable impact on postprandial glucose excursions.

Results of a meta-analysis of 16 RCTs with ≥24 weeks of follow-up using lifestyle interventions including diet and exercise, showed a four-fold increased probability of metabolic syndrome remission compared to pharmaceutical interventions [203]. Another meta-analysis of 20 RCTs (9 with people with IGT, and 11 with T2DM) with diagnosis of 6–48 months duration showed that lifestyle interventions lowered T2DM risk by 20% in people with IGT after 10 years of follow-up but did not reduce all-cause mortality in T2DM [204]. A more recent meta-analysis of 28 RCTs showed that lifestyle interventions were more effective than the standard care regarding the glycemic control of subjects with T2DM, particularly when there was weight loss [29]. These results have also been reported in other systematic reviews and meta-analyses all supporting the beneficial effects of lifestyle interventions, regardless of study design, on weight loss and weight maintenance, delay of T2DM onset and progression and decreased risk for CVD [24,27,28,205,206,207,208,209,210], as well as reduced or eliminated need for anti-diabetic medications [27,127,128,148]. Both shorter-term (6–12 months) and longer-term (>12 months) studies showed that more intensive lifestyle interventions (i.e., medications alone vs medications plus diet plus 5–6 aerobic training sessions/week, duration 30–60 min, of which 2 to 3 sessions were combined aerobic and resistance training) led to greater HbA1c reduction (6.5% compared to 1% with less intensive interventions) [148,149,150] and significant reduction in antidiabetic medications (up to 73% with lifestyle interventions compared to 23% in the control group) [148]. Studies have also shown that the beneficial metabolic changes (body weight loss of 6%, decreased HbA1c by ~0.4%, decreased insulin resistance/HOMA-IR index, ameliorated postprandial hyperglycemia, decreased BP, increased HDL cholesterol, and decreased plasma triglycerides) may last in the long-term [123,154,155]. One study showed that after lifestyle-induced weight loss, improvements in insulin secretion in older, obese, nondiabetic subjects seemed to be largely dependent on improved insulin sensitivity. However, in older, obese diabetic subjects, improved insulin secretion was a consequence of better β-cell function, demonstrating that changes in insulin secretion after lifestyle interventions (including fully supervised aerobic treadmill-walking exercise conducted for 1 h/day, 5 days/week, with increased intensity at week 4 maintaining 80–85% maximum heart rate) may be mediated via alterations in the secretion of incretins, such as GIP [211].

In conclusion, the combination of a reduced energy diet and regular moderate to high intensity physical activity aiming at moderate body weight loss and maintenance, and nutritional behavior modification may ameliorate postprandial hyperglycemia and IR.

### 3.1. Metabolic Effects of Different Types of Physical Activity

In general, exercise can be classified as aerobic (long duration exercise involving a large number of muscles, such as brisk walking or jogging, swimming, cycling) or anaerobic (shorter duration exercise such as sprinting or weightlifting using free weights/weight machines/elastic resistance bands), depending on the predominant mechanisms used by muscle to provide energy [212]. HIIT is also a popular form of aerobic training consisting of short periods of intense exercise interrupted by short periods of active recovery or rest (e.g., such as using a stationary cycle ergometer) [213]. For the working muscle, a constant supply of ATP is required; this is accomplished via either the anaerobic or the aerobic metabolic pathways, which do not function independently but rather synergistically by interactions between the exercising muscles and distant organs/tissues (liver/adipose tissue/cardiovascular system/brain) to provide energy and maintain blood glucose levels within the normal euglycemic range (reviewed in detail in reference [12]).

#### 3.1.1. Aerobic Exercise

At rest (typically during sleeping at night), skeletal muscle derives most of its energy from the oxidation of NEFA than that of glucose [214]. James et al., first reported in rats that aerobic exercise increased the sensitivity of skeletal muscle and adipose tissue but not liver to insulin using euglycemic-hyperinsulinemic clamps with infusions of radiolabeled glucose; the rates of glucose oxidation in muscle were increased [215].

During exercise, the rapid increase in energy demands in the working muscles requires a substantial change in the mobilization and oxidation of carbohydrates and lipids depending on the intensity and duration of exercise. In a systematic study, Romijn et al. [216] described the effects of aerobic exercise intensity (low/25% VO_2max_, moderate/65% VO_2max_, or high/85% VO_2max_) and duration (30 or 120 min) on the utilization of glucose and NEFA in endurance-trained cyclists in the fasting state on 3 consecutive days using a stationary cycle ergometer; indirect calorimetry and infusions of stable isotopes were used to estimate energy turnovers and substrate mobilization; the mechanisms are as follows [216]: (a) At the onset of exercise, insulin secretion is rapidly decreased and that of glucagon increased. The sympathetic nervous system is activated to mediate increases in catecholamines (their levels in plasma increase from ~2-fold to ~4-fold and ~300-fold at low, moderate, or high intensity exercise, respectively) to maintain BP, increase blood flow rates in muscle for the effective delivery of hormones and substrates, and facilitate increases in endogenous glucose production and lipolysis in adipose tissue depots; plasma levels of growth hormone and cortisol also increase with exercise intensity to supplement catecholamine effects [216,217,218,219]. The decrease in plasma insulin levels and the increase in anti-insulin hormones stimulate hepatic glucose production (~1.5-fold, ~4-fold, or ~6-fold during low, moderate, or high intensity exercise, respectively) via glycogenolysis/gluconeogenesis to maintain plasma glucose levels within the normal range and increase the rates of lipolysis in the adipose tissue to release NEFA and glycerol [216,217,219]. The rates of glucose uptake in the contracting muscle increase independently of insulin due to the translocation of GLUT4 transporters from intracellular pools to the surface membrane; hexokinase activity and the rates of glucose phosphorylation also increase [217,220,221,222]. (b) At low intensity exercise, muscle derives energy mainly from the utilization of circulating NEFA produced by lipolysis in the peripheral adipose tissue (~90%) rather than glucose (~10%); the subtle increase in glucose oxidation is met solely by glucose uptake from the circulation and therefore, muscle glycogen is not utilized. Lipolysis from intramuscular triglycerides is not increased [216]. (c) When the intensity of exercise increases from low to moderate, there is a progressive shift from the utilization of NEFA (~50%; derived from the circulation or from muscle triglycerides with equal contribution) to that of glucose (~50%, mostly from muscle glycogen stores, for anaerobic metabolism and production of lactate) [216,223,224]. (d) When the intensity of exercise increases further from moderate to high, energy consumption relies almost exclusively to glucose (derived ~10% from the circulation, and ~60% from muscle glycogen) than to NEFA (~30%; from the circulation or from muscle triglyceride stores) [216], (e) Increase in the duration of exercise from 30 to 120 min does not modify substrate contribution at low intensity; however, at moderate/high intensity there is a progressive increase in the reliance on circulating NEFA and glucose, leading to a decrease in muscle glycogen stores. (f) In healthy subjects (age ~64 years, BMI ~34 Kg/m^2^) the combination of diet intervention with aerobic exercise (treadmill walking or cycle ergometer at ~80% maximum heart rate, 5 days/week, 60 min/day for 12 weeks) improved insulin sensitivity in muscle (euglycemic-hyperinsulinemic clamps), and increased the expression of genes regulating enzyme activities and oxidative capacity in the mitochondria (muscle biopsies) improving NEFA transport and oxidation in this tissue; interestingly, these effects were independent of weight loss (8–10%) and the glycemic index of the diets [225].

During exercise, the rapid increase in energy demands in the working muscles requires a substantial change in the mobilization and oxidation of carbohydrates and lipids depending on the intensity and duration of exercise. In a systematic study, Romijn et al. [216] described the effects of aerobic exercise intensity (low/25% VO_2max_, moderate/65% VO_2max_, or high/85% VO_2max_) and duration (30 or 120 min) on the utilization of glucose and NEFA in endurance-trained cyclists in the fasting state on 3 consecutive days using a stationary cycle ergometer; indirect calorimetry and infusions of stable isotopes were used to estimate energy turnovers and substrate mobilization; the mechanisms are as follows [216]: (a) At the onset of exercise, insulin secretion is rapidly decreased and that of glucagon increased. The sympathetic nervous system is activated to mediate increases in catecholamines (their levels in plasma increase from ~2-fold to ~4-fold and ~300-fold at low, moderate, or high intensity exercise, respectively) to maintain BP, increase blood flow rates in muscle for the effective delivery of hormones and substrates, and facilitate increases in endogenous glucose production and lipolysis in adipose tissue depots; plasma levels of growth hormone and cortisol also increase with exercise intensity to supplement catecholamine effects [216,217,218,219]. The decrease in plasma insulin levels and the increase in anti-insulin hormones stimulate hepatic glucose production (~1.5-fold, ~4-fold, or ~6-fold during low, moderate, or high intensity exercise, respectively) via glycogenolysis/gluconeogenesis to maintain plasma glucose levels within the normal range and increase the rates of lipolysis in the adipose tissue to release NEFA and glycerol [216,217,219]. The rates of glucose uptake in the contracting muscle increase independently of insulin due to the translocation of GLUT4 transporters from intracellular pools to the surface membrane; hexokinase activity and the rates of glucose phosphorylation also increase [217,220,221,222]. (b) At low intensity exercise, muscle derives energy mainly from the utilization of circulating NEFA produced by lipolysis in the peripheral adipose tissue (~90%) rather than glucose (~10%); the subtle increase in glucose oxidation is met solely by glucose uptake from the circulation and therefore, muscle glycogen is not utilized. Lipolysis from intramuscular triglycerides is not increased [216]. (c) When the intensity of exercise increases from low to moderate, there is a progressive shift from the utilization of NEFA (~50%; derived from the circulation or from muscle triglycerides with equal contribution) to that of glucose (~50%, mostly from muscle glycogen stores, for anaerobic metabolism and production of lactate) [216,223,224]. (d) When the intensity of exercise increases further from moderate to high, energy consumption relies almost exclusively to glucose (derived ~10% from the circulation, and ~60% from muscle glycogen) than to NEFA (~30%; from the circulation or from muscle triglyceride stores) [216], (e) Increase in the duration of exercise from 30 to 120 min does not modify substrate contribution at low intensity; however, at moderate/high intensity there is a progressive increase in the reliance on circulating NEFA and glucose, leading to a decrease in muscle glycogen stores. (f) In healthy subjects (age ~64 years, BMI ~34 Kg/m^2^) the combination of diet intervention with aerobic exercise (treadmill walking or cycle ergometer at ~80% maximum heart rate, 5 days/week, 60 min/day for 12 weeks) improved insulin sensitivity in muscle (euglycemic-hyperinsulinemic clamps), and increased the expression of genes regulating enzyme activities and oxidative capacity in the mitochondria (muscle biopsies) improving NEFA transport and oxidation in this tissue; interestingly, these effects were independent of weight loss (8–10%) and the glycemic index of the diets [225].

#### 3.1.2. Anaerobic Exercise

Exercising against resistance (strength training) has gained popularity since it can improve body composition by increasing lean body mass, an effect aerobic exercise practically does not have. Although the mechanisms increasing glucose uptake into muscle cells are generally the same as those described earlier in this section, in contrast to aerobic exercise that requires a complex interplay between glucose and lipids, anaerobic exercise (such as weightlifting or sprinting) requires only glucose derived from muscle glycogen for ATP production [12]. The anaerobic metabolism of glucose in the glycolytic pathway will produce lactate which will be turned back into glucose in the liver. The recycling of glucose between muscle and liver constructs a “substrate cycle” (Cori cycle). The role of substrate cycles in metabolic pathways is important since they can improve the sensitivity of the pathway to external signals, such as hormones (e.g., catecholamines through increases in the sympathetic nervous system activity during exercise) [57]; they also produce heat, an aspect of substrate cycling that is involved in weight control and, therefore, obesity [226]. In anaerobic exercise the “Cori cycle” is of major importance since it is responsible for providing most of the ATP during muscle contractions [12].

An important effect of resistance exercise is that in can increase muscle mass quantity but also quality through the IGF-1/phosphatidylinositol-3 kinase/protein kinase-B pathways [227]. Resistance exercise but not aerobic exercise in rats has been shown to increase IGF-1 expression and subsequent GLUT-4 translocation and increase of glucose uptake in skeletal muscle preparations in-vitro [228]; increases in plasma IGF-1 concentrations have also been reported in humans during high-intensity resistance training [229]. Earlier studies in rats showed that IGF-1, with its insulin-like effects, increases the apparent sensitivity of skeletal muscle to insulin either in-vitro [230] or after acute or chronic (10 days) administration in-vivo [231].

Muscle hypertrophy with resistance training is popular and has attracted attention regarding its impact on glucose homeostasis and the mechanisms involved. It has been suggested that the increase in insulin-stimulated glucose disposal with resistance exercise may be due, at least in part, to the increase in muscle mass, whereas aerobic training may enhance insulin sensitivity by changes in the intrinsic metabolic pathways in muscle cells [232]. In a comprehensive recent narrative review, Paquin et al, summarized all the available literature on this subject aiming to provide a mechanistic explanation for this type of exercise in improving insulin sensitivity and health outcomes [233]. As these authors [233] conclude, regular resistance exercise improves insulin sensitivity by a number of mechanisms, including greater muscle vascularization and increases in blood flow rates. However, the question if the beneficial effects of resistance exercise are due mostly to muscle hypertrophy or to a better quality of muscle cells needs further investigation [233].

The mechanisms described in this section regarding the crosstalk between tissues for the mobilization of substrates during exercise depend on how much glycogen is stored in the liver or muscle at the beginning of exercise, which in turn depends on the amount of carbohydrates in the previous meal or on any performance of physical activity prior to exercise, as well as on the intensity/duration of exercise, and the physical fitness of the subjects. These factors should be considered since they may explain, at least in part, the discrepancies between studies in the literature.

In subjects with diabetes or obesity, exercise is the cornerstone of therapeutic interventions. It increases insulin sensitivity independently of changes in body fat with nutritional interventions, improves metabolic control, contributes to the decrease in body weight, decreases the risk for cardiovascular/kidney complications and hypertension, and improves physical fitness and well-being [234,235,236,237,238,239,240,241,242].

## 4. Chrononutrition

Most living organisms exhibit circadian (diurnal) rhythms, which essentially control rhythmicity in physiological activities, such as rest/active, and feeding/fasting cycles [243,244]. In mammals, the functions of nearly all organs and systems, such as the pancreas, the gastrointestinal system (including the intestinal microbiome), the adipose tissue, the immune system, the endocrine system, the cardiovascular system, thermoregulation, brain activity, etc. are regulated by circadian rhythms, and may exhibit daily oscillations [243,244]. Endogenous molecular circadian clocks display 24-h oscillations and govern the circadian rhythms [243,244]. The center of these clocks is in the hypothalamus, within the suprachiasmatic nucleus (SCN), which contains neurons oscillating periodically approximately every 24 h, and acts as a “master clock” for the peripheral clock systems present in all other tissues and cells in the body [243,244].

The molecular clocks regulate the transcription of a myriad of clock-controlled genes either directly by the two master heterodimeric transcription factors CLOCK and BMAL1 and other clock regulated transcription factors, or indirectly through other clock output proteins [245,246], to drive rhythmic gene expression and regulate biological functions under circadian control [247]. Also, the heterodimer CLOCK and BMAL1 rhythmically activates the expression of their transcriptional repressors, Period (Per1 and Per2) and Cryptochrome (Cry1 and Cry2) [247]. Several factors involved in glucose metabolism, such as insulin and cortisol are expressed and secreted following circadian stimuli similarly to the main organs involved in glucose uptake and metabolism do (i.e., liver, pancreas, adipose tissue, muscles) [248,249]. Glucose tolerance peaks during daylight and is lower during the night/dark cycle. Insulin follows a temporal control of production, and its release is controlled by both feeding-fasting patterns and circadian rhythms [250]. The levels of nutrients in the blood are a physical signal for insulin production, and the circadian and SCN systems modulate both insulin and glucagon by controlling their synthesis at the cellular level [249,251,252,253]. Animal and in vitro studies with BMAL1−/− and Per1−/− show a disruption on glucose homeostasis and insulin release despite displaying normal activity and feeding-fasting rhythms [251,254]. This demonstrates how the molecular clock at the cellular and tissue levels can have significant effects on physiology [249]. Furthermore, the pancreatic β-cells receive parasympathetic input, which is under circadian control by gamma-aminobutyric acid/GABA-ergic projections from the SCN [252]. Similarly, the SCN has both glutamatergic and GABAergic projections to modulate the liver and influence glucose production [252]. The time of day that the function of the pancreatic β-cells peaks is still unknown, but some data suggest that it may peak during lunch hours; indicating that carbohydrate intake is better metabolized during these hours resulting in ameliorated postprandial hyperglycemia [83]. Finally, cortisol, a steroid hormone involved in metabolism and stress responses, also follows a daily rhythmicity. Adrenal gland cells have cellular clocks to temporally influence production and release of cortisol [249,255]. Peak levels of cortisol synchronize with the beginning of the active phase to aid in arousal (early morning in diurnal animals and early night in nocturnal animals) [255].

The circadian clocks can respond to environmental variables which may act as circadian time cues also known as zeitgebers or time givers or time cues [243]. While light (light/dark cycles in the day) is the most potent time signal and it caters to the SCN clock system, food availability (feeding/fasting cycles) and activity-rest pattern are other important zeitgebers that entertain the peripheral clocks, which dominate local physiological processes such as glucose and lipid homeostasis, hormonal secretion, the immune responses, and the digestive system [243]. Circadian rhythms may also influence energy balance [243]. Synchronization of peripheral clocks is essential to ensure temporally coordinated physiology [243]. Significant variations from this circadian rhythmicity have been shown to disrupt metabolism and homeostasis in many organs and can manifest in different ways ranging from irritability and fatigue to several chronic diseases, including obesity, T2DM, CVD, and inflammation [243]. Unlike the changes in daylight, which are fixed depending on the geographical location, changes in food intake and feeding time in a day can affect nutrient sensing pathways that act to maintain homeostasis [243]. Synchronizing food consumption, food quality, and metabolic rhythms in a day may provide optimal metabolism and positively impact health. In the past years we have valuable information regarding our organism synchronization including the following approximations: melatonin drops at 7:00, cortisol rises at 8:00, at 8:30 we have bowel movements, at 10:00 insulin sensitivity peaks, at 11:30 we have high alertness, at 14:30 muscle performance peaks, at 20:00 melatonin rises, at 22:00 body cools down, at 1:00 sleep deepens, at 3:00 body temperature rises, and so on [256].

The term “chrononutrition” describes the direct relationship between time of day (eating during light time vs evening vs night), eating occasion (consuming or skipping meals, macronutrients’ amount and type, and time of day consumed, main course eaten at early lunch hour before 14:00–15:00 vs late lunch hour after 15:00 vs evening dinner, etc.), the body’s daily circadian rhythms and their effects in metabolic health. This concept reflects the basic idea that, in addition to the amount and content of food ingested, the time of ingestion itself is also critical for the well-being of an organism. As such, one can envision an ‘‘optimal’’ feeding schedule synchronous to the body’s metabolism that may provide benefits for the overall health.

### 4.1. Feeding and Circadian Solidarity 

A study tracking the participants’ eating behaviors via a smartphone app, revealed erratic diurnal feeding patterns [257]. This may be expected considering the modern way of life where late eating (in many cases with over 30% of the daily calories being consumed after 18:00), lack of stable eating patterns, and skipping meals are indeed very common, particularly in western societies (Figure 3). This type of lifestyle can lead to circadian misalignment and negatively impact metabolism and glucose control. Circadian misalignment has been observed in chronic jet lag and night shift workers, who exhibit a lowered glucose and lipid tolerance, increased liver lipogenesis, higher body mass index (BMI), and are prone to various metabolic diseases such as obesity, CVD, gastrointestinal disorders, and T2DM [258,259,260]. In nocturnal rodents, feeding during the daytime hours for a week completely altered the phase of the circadian expression of clock and clock-controlled genes in the peripheral tissues [261,262]. In humans, a circadian misalignment protocol with a 12-h shift of all meals resulted in elevated blood glucose and reduced insulin levels after the evening dinner [263]. Finally, individuals with an inherent later chronotype also tend to have a higher BMI presumably because they consume their meals later in the day [264]. All the above, underline the importance of timing of food consumption.

Timing of food intake according to the body’s circadian rhythms can align the zeitgeber with the metabolic responses from different organs resulting in a healthier postprandial state. Lipogenesis in the liver can also be reset by changes in nutrition modulating fat usage by muscle via the circulating lipids [265,266]. The liver clock is affected by the balance between food intake and starvation intervals [267,268,269]. In this context, because breakfast is consumed after the longest fasting period of the day, it is usually the most important meal to modulate the phase of the liver clock [270]. On the other hand, late evening dinners or midnight snacks change the length of the fasting period and thus alter the phase of peripheral clocks. Regarding the effect of nutrients on the liver clock, a combination of carbohydrate and protein is essential to reset it and cause a phase-shift, whereas protein, carbohydrates, or lipids, when consumed alone, are insufficient [270].

In conclusion, the feeding time (day vs night), the hours of daily fasting until the first meal is consumed and irregular meal patterns may play a significant role in postprandial hyperglycemia and IR, and personalized advice to patients should be provided, taking these factors into consideration.

### 4.2. Effects of Meal Timing on Postprandial Glycemia and IR 

There is intense scientific interest in examining the validity or not of the famous quote “Eat breakfast like a king, lunch like a prince, and dinner like a pauper” (Figure 3). In modern times a tendency for late-eating patterns has been observed and in many cases, it is accompanied by skipping breakfast [257]. One study showed that the unfavorable effects of evening eating on metabolic risk in healthy volunteers were due to lower concentrations of epinephrine/norepinephrine and higher concentrations of acylated ghrelin after the evening dinner (at 20:00), with the opposite behavior exhibited after the morning meal (at 8:00) [271]. Another study in healthy adults showed that eating in the evening (at 17:00) vs morning (at 9:00) resulted in higher postprandial glucose concentrations and GIP, and lower levels of five glycolysis/tricarboxylic acid cycle/nucleotide-related metabolites, and eighteen amino acid-related metabolites, all involved in an exacerbation of postprandial hyperglycemia [272]. Another study showed that transferring 100 kcal of fat intake from night (20:30–05:00) to earlier periods was associated with lower low-density lipoprotein (LDL) cholesterol levels, especially when transferred to lunch time (11:30–13:30) or evening (17:30–20:30) [273]. In a large cross-sectional study with 11.594 subjects with T2DM, evening vs morning chronotype was independently associated with T2DM, and people had lower odds of having HbA1c at the recommended levels of <7% [274].

Table 2 describes the effects of the first meal of the day on indices of glycemic control. Several studies have suggested that skipping breakfast may be associated with increased BMI (change in BMI from baseline over time), altered lipid and glucose homeostasis, increased LDL, metabolic syndrome and T2DM [260,275,276,277,278], although reverse causality cannot be ruled out, i.e., people may feel fuller when obese and so more likely to skip breakfast. Regular breakfast consumption has also been shown to increase satiety and thermogenesis and improve the quality of the diet by inclusion of fibers and nutrient-rich foods [279,280]. The effect of breakfast on weight loss is controversial with some short-term studies showing a modest effect with a slightly greater weight loss in individuals who consumed breakfast [281], longer term prospective studies showing no clinically significant effect [276], and others in healthy individuals showing minor differences in nutritional and perceived characteristics of breakfast regarding medium-term [282].

Similarly, the effects of breakfast consumption on postprandial glycemia and IR is also controversial. Results from few RCTs in lean and obese subjects suggest that skipping breakfast regardless of body weight may adversely affect insulin sensitivity [280,283]. A recent meta-analysis of prospective cohort studies reported that breakfast omission was associated with 55% greater risk of T2DM compared with breakfast consumption [277]. In a 16-year follow up cohort, men who skipped breakfast had higher risk for developing IR and T2DM [278]. Additionally, individuals with later chronotypes and a pre-existing history of T2DM were reported to have worse glycemic control when breakfast was skipped [284]. However, reverse causality cannot be ruled out, i.e., the increased risk for IR and T2DM may be due to the higher BMI and not to skipping breakfast.

A few clinical trials have been conducted in individuals with prediabetes or T2DM (summarized in Table 2). Breakfast omission, and thus continued fast until lunch in T2DM, overweight or obese individuals was shown to result in reduced insulin and GLP-1 after lunch (the first meal of the day in one study), and lower glycemic and insulinemic responses, leading to postprandial hyperglycemia after lunch consumption [72,283,285]. In contrast, healthy individuals skipping breakfast had better glycemic control after lunch, with blood glucose levels slightly elevated or unchanged and insulin secretion and sensitivity slightly increased [286,287]; only glucose variability during the day was reported to be higher in the fasting group [280]. A Japanese study in healthy individuals showed that breakfast omission together with late-evening dinner consumption, but not breakfast skipping alone, resulted in hyperglycemia [288]. Another study examining the acute effects of breakfast consumption or omission in both healthy individuals and T2DM, reported that breakfast consumption vs breakfast skipping affected positively clock and clock-controlled gene expression leading to normal oscillation patterns, while skipping breakfast resulted in increased postprandial glycemic responses [285]. Few available RCTs suggest that the reduced insulin sensitivity observed later in breakfast skippers may be due to the anti-insulin hormones peaking in the morning hours (i.e., cortisol), the prolonged elevation of plasma NEFA levels due to the extension of overnight fast, and the low recruitment of GLUT-4 transporters and insulin-stimulated glucose uptake in muscle (post-fasting) [289].

#### 4.2.1. Effects of Meal Macronutrient Composition on Postprandial Glucose and IR

The carbohydrate type and content of a meal are the main determinants of postprandial hyperglycemia and insulin response (Figure 3). Although the amount of carbohydrate intake required for optimal health is unknown, an intake of >130 g/day seems to be required for brain’s demand for glucose, and of body’s metabolic processes, including glucose metabolism [114]. Breakfast is the most studied meal (summarized in Table 2). Possibly breakfast hours may be the worst time of the day to consume high amounts of carbohydrates, while small amounts would initiate an increase in insulin sensitivity. Thus, limiting the readily available carbohydrates in breakfast and replacing them with other energy and nutrient sources may be beneficial for achieving better glucose regulation and lower glycemic excursions [84].

Results of a recent meta-analysis of prospective cohort studies with 4–26 years follow-up showed a role for GI and GL as causal factors contributing to incident T2DM and recommended considering the inclusion of GI and GL in food and nutrient-based recommendations [290]. Another recent meta-analysis reported that consuming low GI and GL at breakfast attenuated acute postprandial hyperglycemia [291]. Consumption of fibers at breakfast (i.e., beta glucans, whole grain cereals) may slow the rates of gastric emptying and intestinal glucose absorption, and contribute to increased satiety, thus reducing postprandial glucose responses in a dose-dependent manner [292]. We have shown in a series of studies that foods (such as Ceratonia siliqua, carob) and characteristics (such as large bran size or the sucrose to oligosaccharide ratio in honey varieties of similar botanical origin and characterization explaining 30% of the postprandial glucose differences) are able to reduce glucose excursions and overall postprandial glycemic responses [293,294,295], indicating the need for more clinical trials investigating the effects of functional foods on postprandial glycemia and IR.

Accordingly, protein and fat consumption at breakfast have been shown to increase satiety and better regulate post-meal glucose responses [275,276]. Long term substitution of available carbohydrates at breakfast with proteins also decreased blood triglycerides and regulated BP [275]. Short- and long-term clinical trials in subjects with T2DM [84,296,297,298,299,300] (Table 2 and Table 3) suggested that consuming more lower fat containing proteins than carbohydrates at breakfast help to reduce postprandial hyperglycemia, insulin responses and blood lipids. These studies had varying protocols and meal compositions; nevertheless, it seems that including proteins and fats and not only carbohydrates at breakfast may be beneficial for glycemic regulation.

**Table 2:** Effect of First meal of the Day on Glycemia in healthy individuals and subjects at high risk for developing or with type 2 diabetes.

Study	Health Status Age (Years) BMI (kg/m^2^)	Duration & Design of Dietary Intervention	Sample Size	Description of Groups	Dietary Intervention	Selected Clinical Outcomes
Effect of first meal of the day on glycemia.						
Breakfast skipping or consuming						
Morris, et al., 2015 [263]	Healthy 28 ± 9 25.4 ± 2.6	2 weeks Within-participant cross-over	14	Circadian alignment Circadian misalignment (12 h shift)	Alignment protocol had B at 8:00 AM Misalignment protocol had “B” at 8:00 PM Isocaloric diets of 15–20% PRO, 45–50% CHO, 30–35% FAT	+8% and +14% ppd AUC_Glu_ and ppd AUC_Ins_ at Dinner time +3% ISR at Dinner time +12% and −27% ppd AUC_Glu_ and ppd AUC_Ins_ in biological evening +8% ISR in biological evening −21% Fasting Ins in biological evening +14% and +9% late phase Ins/ISR and 24 h Ins at circadian misalignment
Betts, et al., 2014 [280]	Healthy lean 36 ± 11 22.4 ± 2.2	6 weeks Randomized controlled trial	33	B group Fasting group	B: ≥700 kcal before 11:00, Fasting group: extend o/n fast until 12:00, ad libitum intake for the rest of the day	Glu (mg/dL) +1.3 (fast) vs +1.1 (B) Ins (μIU/mL) +0.32 (fast) vs +0.35 (B) HOMA-IR +0.10 (fast) vs +0.10 (B) C-ISI Matsuda index +0.38 (fast) vs −0.97 (B) Index of adipose insulin sensitivity (%) +3.3 (fast) vs +9.9 (B) Peak Glu until 12:00 + 1.1 mmol/L in B vs fasting Mean morning Glu +0.3 mmol/L in B vs fasting Greater Glu variability in fasting group
Chowdhury, et al., 2016 [283]	Obese 44 ± 10 33.7 ± 4.9	6 weeks Randomized controlled trial	23	B group Fasting group	B: ≥700 kcal before 11:00, Fasting group: extend o/n fast until 12:00, ad libitum intake for the rest of day	Fasting Glu (mg/dL) +1.7 (fast) vs +1.4 (B) Fasting Ins (μIU/mL) −0.62 (fast) vs +0.39 (B) HOMA-IR −0.13 (fast) vs +0.18 (B) C-ISI Matsuda index −0.05 (fast) vs +0.05 (B) Ins AUC Glu, mg·120 min/dL +171 (fast) vs −231 (B)
Jakubowicz, et al., 2017 [285]	Healthy: 44.3 ± 2.9 23.1 ± 0.4 T2D: 66.8 ± 1.9 30.7 ± 1.1	2 test days Randomized open-label crossover-within-subject clinical trial	32	YesB NoB	Each test meal: 572 ± 8 kcal 32% PRO 49% CHO 19.4% FAT	+15–18% AUC_Glu_ after lunch w/o B −25% AUC_Ins_ after L for T2DM grp w/o B −35% AUC_iGLP-1_ after L on NoB
Nas, et al., 2017 [286]	Healthy adults 24.6 ± 3.3 23.7 ± 4.6	3 test days Randomized crossover nutritional intervention	17	Control (C) (3 meals) BSD (B skipping) DSD (D skipping)	Isocaloric diets 55% CHO, 30% FAT, 15% PRO BSD-washout-C-DSD or DSD-washout-C-BSD	HOMA-IR 1.96 ± 0.82 (C), 2.07 ± 0.91 (BSD), 1.96 ± 1.05 (DSD) Glycemia_tAUC_ (mg/dLx24 h): 2360 ± 111 (C), 2425 ± 131 (BSD), 2374 ± 165 (DSD) MAGE 3.90 ± 1.32 (C), 3.65 ± 1.52 (BSD), 3.28 ± 1.75 (DSD) C-peptide (μg/day) 74 ± 38 (C), 86 ± 40 (BSD), 75 ± 42 (DSD) iAUC_Ins_ (μU/mLx 2 h) after L: 211 ± 74 (BSD) 144 ± 74 (DSD) iAUC_Glu_ (mg/dLx 2 h) after L: 114 ± 41 (BSD), 62 ± 40 (DSD) HOMA-IR pp after L: 59 ± 44 (BSD), 27 ± 23 (DSD)
Kobayashi, et al., 2014 [287]	Healthy 25.3 ± 1.2 BW 74.5 ± 4.3 kg (noB), 73.9 ± 4.2 kg (B)	2 test days Randomized crossover	8	B (3 meals) noB (2 meals)	PRO 364 ± 16 kcal CHO 1310 ± 77 kcal FAT 462 ± 33 kcal Individually adjusted meals of 2190 ± 124 kcal/day B at 8:00 h, L at 12:00 h, D at 19:00	+9 mg/dL Blood Glu after L in noB vs B group (*p* < 0.05) +10 mg/dL sleep Blood Glu in noB vs B group (*p* < 0.05) AUC_Glu_ after L 409 ± 99 mg/dL min in B group vs 811 ± 101 mg/dL min in noB group AUC_Glu_ after D 1049 ± 144 mg/dL min in B group vs 1196 ± 204 mg/dL min in noB group
Jakubowicz, et al., 2015 [301]	T2DM 56.9 ± 1.0 28.2 ± 0.6	2 test days Randomized, open-label, crossover- within-subject clinical trial	22	YesB (B, L, D) NoB (L, D)	Each test meal: 701 ± 8 kcal; 26% PRO, 54% CHO, 20% FAT, 7% fiber B at 8:00 h, L at 13:30 h, D at 19:00 h	NoB vs YesB after B: Glu (mg/dL·min) −43%, Ins (μIU/mL·min) −72.1%, Glucagon (pg/mL·min) −20.6% C-peptide (ng/mL·min) −63.3%, iGLP−1 (pmol/L·min) −60.5% NoB vs YesB after L: Glu (mg/dL·min) +39.8%, Ins (μIU/mL·min) −24.7%, Glucagon (pg/mL·min) +9.7% C-peptide (ng/mL·min) −13.6%, iGLP−1 (pmol/L·min) −21.5% NoB vs YesB after D: Glu (mg/dL·min) +24.9%, Ins (μIU/mL·min) −10.8%, Glucagon (pg/mL·min) +8.5% C-peptide (ng/mL·min) −14.5%, iGLP-1 (pmol/L·min) −14.5%
Breakfast composition						
Kang, at al., 2013 [84]	Subjects with prediabetes and normal (NGR) Glu regulation 46.4 ± 13.8 18.5–24.9	3 days Cross-sectional study	133	LC (low CHO) MC (medium CHO) HC (high CHO)	Diet of 30 kcal/kg/day calorie intake from three daily meals According to CHO in B: Low carbohydrate (LC) meal with <45%CHO Medium-carbohydrate (MC) meal with CHO between 45% and 65% High-carbohydrate (HC) meal with >65% CHO	In subjects with impaired Glu regulation: Significantly ↑ ppd Glu, Glu peak, Glu excursion, and iAUC_GLU_ in subjects with impaired Glu regulation after B with >50% CHO
Rosi, et al., 2018 [282]	Healthy 24 ± 2 23.4 ± 1.6	7 weeks Randomized, crossover, and controlled trial	15	F-CTRL BR-BREAD BR-MUESLI BR-RICE	Energy-free meal with a cup of decaf coffee (~fasting) 3 isoenergetic meals with similar PRO: with a cup of semi-skimmed milk, an apple, and cereal foods as indicated below: White bread with chocolate hazelnut spread, GI <55, GL~22 Muesli with dark chocolate chips and nuts,↑fiber, GI <55, GL~23 Chocolate-flavored puffed rice, ↓FAT, ↑CHO, GI >55, GL~38	The RICE group had significantly higher: -AUC_Ins_ 120 min after B -AUC_Glu_ 120 min after B -Plasma Glu after B
Jakubowicz, et al., 2017 [296]	T2DM 59.0 ± 0.7 32.11 ± 0.1	12 weeks Randomized, open-label, parallel-arm clinical trial	48	42 g total PRO: WBdiet (whey, 28 g) PBdiet (42 g various PRO sources) CBdiet (high CHO B, 17 g PRO from various sources)	At B: WBdiet: 25% PRO (mainly whey), 50% CHO, 25% FAT PBdiet: 25% PRO (mainly from eggs, tuna, soy), 50% CHO, 25% FAT CBdiet: 11% PRO (soy), 64% CHO, 29% FAT Hypocaloric diets: B 660 ± 25 kcal L 560 ± 20 kcal (23% PRO, 48% CHO, 29% FAT) D 280 ± 15 kcal (31% PRO, 31% CHO, 38% FAT) B at 6:00–8:30 h, L at 12:30–14:30 h, D at 18:30–20:30 h	HbA_1C_ (%) WB −0.89 ± 0.05 PB −0.6 ± 0.04 CB −0.36 ± 0.04 Fasting Glu (mmol/L) WB −0.73 ± 0.06 PB −0.43 ± 0.06 CB −0.12 ± 0.04 Overall glycemia was −12% in PB and −19% in WB Glu peak was −18% in PB and −31% in WB Rapid Glu levels decrease after B in PB and WB Overall AUC_Ins_ was +37% in PB and 62% in WB (same for after L and D) AUC_C-pept_ was +53% in PB and 96% in WB (same for after L and D) AUC_iGLP_1_ was +70% in WB and +33% in PB after B, L, and D HbA_1C_ reduced in all grps
Jakubowicz, et al., 2012 [297]	20–65 32.3 ± 1.8	32 weeks Randomized, treatment controlled, openclinical trial	144	LCb HCPb	Low kcal and low CHO diet (LCb) with low kcal and low HCO B High CHO and high PRO diet (HCPb) with daily dessert for B Similar L & D composition, differences for B	BW (kg) 70.6 ± 8.7 (HCPb) vs 86.9 ± 9.7 (LCb) Fasting Glu (mg/dL) 84.2 ± 4.6 (HCPb) vs 95.5 ± 4.9 (LCb) Fasting Ins (μU/mL) 8.9 ± 3.9 (HCPb) vs 23.69 ± 3.8 (LCb) HOMA-IR 1.6 ± 0.4 (HCPb) vs 5.9 ± 0.9 (LCb) Total Chol (mg/dL) 179.2 ± 11.1 (HCPb) vs 190.8 ± 18.2 (LCb) TG (mg/dL) 122.6 ± 9.7 (HCPb) vs 174.5 ± 20.9 (LCb) ↑ hunger in LCb
Neumann, et al., 2016 [298]	Healthy 24.1 ± 2 n/a	8 days Randomized, controlled study	24	SKP (Skipping breakfast) CHO PRO	SKP group CHO group: 351 kcal; 59 g CHO, 10 g PRO, 8 g fat PRO group: 350 kcal; 39 g CHO, 30 g PRO, 8 g Fat B consumed as first meal of the day, before 10:00 am	No difference in fasting blood Glu CHO and PRO groups lead to greater ppd Glu vs SKP ↓10% Glu in PRO vs CHO at 30 min ppd Ffter B
Pedersen, et al., 2016 [299]	Obese/T2DM 63.9 ± 2.15 33 ± 1.25	4 exp. days Randomized crossover study	28	CHO-B noCHO-B	Fast ≥8 h before the test diets The diets were consumed on 2 sequential days on separate weeks 3 identical meals with CHO 3 meals, no CHO breakfast, lunch and dinner with CHO –similar to other group	Peak blood Glu (mmol/L) 11.3 ± 0.5 after CHO-B 9.4 ± 0.4 after noCHO-B Mean blood Glu (mmol/L)—5 h after B 8.4 ± 0.5 after CHO-B 7.5 ± 0.4 after noCHO-B no sig. differences in Glu measurements after lunch and dinner no sig. differences in gastric emptying
Rabinovitz, et al., 2014 [300]	Overweight/obese with T2DM 60.7 ± 6.35 32.37 ± 3.7	3 months Randomized, treatment-controlled, open clinical trial	46	SB (small breakfast) BB (big breakfast)	At B: 12–18% PRO, 14–22% FAT, 60–70% CHO, 13% of total E was recommended in the SM, Lunch and Dinner had 33% of total daily E, 2–3 snacks same as in BB At B: 23–30% PRO, 29–37% FAT, 37–48% CHO 33% of total E was recommended in the BB, Lunch and Dinner had 25% of total daily E, 2–3 snacks same as in SM	BW no sign. difference HbA1c (%) −0.58 ± 0.18 in BB −0.13 ± 0.08 in SB Estimated average glucose (mg/dL) −16.6 ± 5.2 in BB −3.43 ± 2.4 in SB no sign. changes in Glu, Ins, C-peptide, total Chol, TG, CRP, IL-6, TNF-α

Abbreviations: B: Breakfast; L: Lunch; D: Dinner; YesB: had breakfast; NoB: skipped breakfast; T2DM: type 2 diabetes; ppd: postprandial; GI: Glycemic Index, GL: Glycemic Load; BSD: breakfast skipping day; DSD: dinner skipping day; PRO: protein; CHO: carbohydrates; FAT: fat/lipids; HOMA-IR: homeostatic model assessment for insulin resistance; AUC: area under the curve; iAUC: incremental area under the curve; Ins: insulin concentrations; Glu: glucose concentrations; HbA1c: glycated hemoglobin A1c; ISI: insulin sensitivity index; WB: body weight; E: total energy intake; SKP: skipping breakfast. An arrow pointing upwards or downwards indicates an increase or decrease.

Two studies from our group, an acute [302] and a short-term (8 weeks duration) [143] cross-over RCT, and a 2013 meta-analysis of studies ranging from 4–24 weeks, reported that high-protein eating plans (25–32% of total energy vs 15–20%) resulted in 2 kg greater weight loss, better maintenance of muscle mass, and 0.5% greater improvements in HbA1c, without significant decreases in fasting glucose [303]. However, it should be noted that the protein sources that seem to have beneficial effects on postprandial hyperglycemia and IR are possibly either lean animal proteins or plant proteins, although this remains to be elucidated. Similar contradictory results on postprandial hyperglycemia and IR have been reported in respect to fats, with some supporting monounsaturated fats, such as extra-virgin olive oil and nuts [103], others supporting saturated fat from dairy products, coconut oil and palm kernel oil [304] and others reporting that saturated fat intake was associated with higher risk of T2DM [305].

#### 4.2.2. The Effects of Consuming Most Food and Calories Earlier in the Day on Glycemia 

Due to the daily oscillations of various hormones and the two-way interaction between food consumption and metabolism, a lot of attention has been given on what the energy and food intake distribution in a day should be, the number of meals consumed, the consistency of meal timing, and the time of day that these meals should be consumed, in order to ameliorate postprandial hyperglycemia and IR. Meal timing has been reported to synchronize and boost the peripheral circadian clocks that control downstream metabolic pathways (Figure 3) [306]. Earlier efforts using mixed meals or glucose infusion demonstrated circadian responses of reduced glucose tolerance and insulin sensitivity in healthy participants for the evening hours rather than in the morning [260]. So, the same foods distributed differently throughout the day, appear to have different effects on glycemic control in subjects with prediabetes and/or T2DM. Recently more studies have been conducted to address this issue. Table 3 describes the effects of lunch and dinner on indices of glycemic control. Having a caloric rich breakfast was found to result in increased postprandial insulin secretion and GLP-1 responses, and smaller plasma glucose peaks after breakfast consumption [301]; these effects were not present when the majority of calories was consumed at the evening dinner. The effects of the earlier increases in plasma insulin levels persisted after lunch aiding the glycemic management of the subsequent meals (the “second meal effect”). Results from a 7-days/randomized/open-label/crossover trial with 18 subjects with T2DM, performed in two separate testing days, each over the course of 14 h, showed that eating the majority of calories at breakfast (at 08:00) vs dinner (at 19:00) at home for 6 days before each testing day (30–50% of daily calories at breakfast, i.e., 704 kcal breakfast, 605 kcal lunch and 205 kcal dinner vs 205 kcal breakfast, 605 kcal lunch and 704 kcal dinner), with the “large” meal (breakfast or dinner respectively, containing 22% fat, 47% protein), or the “small” meal (dinner or breakfast, respectively, with 30% fat, 27% carbohydrates, 43% protein), led to reduction in overall postprandial hyperglycemia [301]. Results from another study with 12 healthy young females examining the effects of a late suppertime (18:00 vs 23:00) on gastrointestinal activity the following morning (efficiency of digestion/absorption of dietary carbohydrates ingested at the usual suppertime), showed that a late supper was associated with a worse effect on postprandial glucose profiles the following morning [307]. A pertinent question arising is how the body recognizes calories. Maybe it doesn’t in the short term in which case a high calorie breakfast is a meaningless way to describe a meal being investigated for acute metabolic effects.

On the other hand, skipping the evening dinner (Table 3) may improve glucose intolerance, and lower IR [286,308]. In contrast, eating a rich or a late dinner may aggravate IR and hyperglycemia and deteriorate blood glucose levels the following morning [307,309]. It has also been shown that moving the evening dinner to an earlier time may improve glucose tolerance due to a causal role of endogenous melatonin in the impairment of glucose tolerance, particularly in MTNR18 carriers [310]. Unfavorable effects on postprandial glycemia and overall glycemic control have been reported during Ramadan fasting, where people abstain from eating/drinking during daylight and consume all energy at night [311].

Likewise, consuming lunch later rather than early (after 15:00–16:00 h) in the day, has also been associated negatively with glycemic control (Table 2). Eating lunch late increased IR (higher HOMA-IR) in overweight and obese subjects [312] and increased postprandial blood glucose in healthy individuals [309]. Also, one acute study showed that consuming a snack in the afternoon, but not right after lunch, improved the mean amplitude of glycemic excursions [313]. Furthermore, consuming more carbohydrates at lunch time (Table 4) and more fats later rather than earlier, reduced fasting blood glucose, insulin, and GLP-1 responses. Whole day blood glucose fluctuations were also maintained lower when carbohydrates were consumed earlier than later in the day [314]. Additionally, a short-term trial in healthy individuals demonstrated that consumption of high GI foods was better managed during daytime hours, whereas high GI food intake at dark hours (late afternoon-evening hours) resulted in greater blood glucose peaks and total blood glucose concentrations after meals [315]. Together, these data suggest that carbohydrates should be consumed primarily at lunch and early afternoon hours. Early in the day is a fasting state in which the glycemic responses to carbohydrates are high (poor due to IR), whereas later in the day glycemic responses are better (lower) since preceding meals may sensitize the metabolic and incretin systems to the following ones, thereby improving glucose tolerance [31]. In this regard, although some carbohydrates must be consumed at breakfast to improve glucose tolerance in the following meals, placing the majority of carbohydrates at breakfast may not be a good idea; actually, breakfast and evening dinner should have the minority of carbohydrates with lunch taking the most. Such effect is consistent with the circadian biology of glucose metabolism and does support that following dietary patterns in accordance with the diurnal bodily rhythms can be a useful approach in the management of glycemia. Moreover, consuming most of the food and calories at lunch and early afternoon hours, and not during the night hours may be beneficial for satiety and hunger regulation. Hunger is intrinsically modulated and has circadian periodicity with peaks in the evening, meaning that there is higher tendency for food later in the day. Ghrelin, an orexigenic hormone, increases just before a meal and especially at night [316], but is being suppressed by increases in insulin. Therefore, having a large, rich breakfast may also contribute to hunger, cravings, and postprandial ghrelin reduction [266].

**Table 3:** Effect of Lunch and Dinner on glycemia in healthy and individuals at high risk for developing or with type 2 diabetes.

Study	Health Status Age (Years) BMI (kg/m^2^)	Duration and Design of Dietary Intervention	Sample Size	Description of Groups	Dietary Intervention	Selected Clinical Outcomes
Effect of Lunch on Glycemia						
Lunch timing & Glycemia						
Bandin, et al., 2015 [309]	Healthy 26 ± 4 22.54 ± 2.05	2 weeks Randomized and crossover (Protocol 1: metabolic study)	10 (subjects on protocol 1)	EE group (Early Eating) LE group (Late Eating)	L at 13:00 h L at 16:30 h Same B (at 8:00 h), D (at 20:00 h), and L as indicated 1868 ± 234 Kcal/day 15% PRO, 50% CHO, 35% FAT	+46% AUC_Glu_ after L in LE vs EE +1 mmol/L Glu 90 min after L in LE vs EE +0.6 mmol/L Glu 120 min after L in LE vs EE
Garaulet, et al., 2013 [312]	Overweight/obese 42 ± 11 31.4 ± 5.4	20 weeks	420	Early Lunch Eaters (EL) Late Lunch Eaters (LL)	Early eaters: Lunch before 15:00 h Late eaters: Lunch after 15:00 h Weight loss diet of similar composition Total E ~ 1400 Kcal/day 19% PRO 48% CHO 33% FAT	Fasting Glu (mg/dL) 81.28 ± 15.97 (EL) vs 83.65 ± 16.27 (LL)—non-sign Fasting Ins (mU/L) 5.72 ± 4.71 (EL) vs 6.98 ± 11.66 (LL)—non-sign HOMA 1.17 ± 0.14 (EL) vs 1.57 ± 0.13 (LL)—significant
Effect of Dinner on Glycaemia						
Dinner timing & composition						
Nas, et al., 2017 [286]	Healthy 24.6 ± 3.3 23.7 ± 4.6	3 test days Randomized crossover nutritional intervention	17	Control (C) (3 meals) BSD (B skipping) DSD (D skipping)	Isocaloric diets 55% CHO, 30% FAT, 15% PRO BSD-washout-C-DSD or DSD-washout-C-BSD	HOMA_IR 1.96 ± 0.82 (C), 2.07 ± 0.91 (BSD), 1.96 ± 1.05 (DSD) Glycemia_tAUC_ (mg/dLx24 h) 2360 ± 111 (C), 2425 ± 131 (BSD), 2374 ± 165 (DSD) MAGE 3.90 ± 1.32 (C), 3.65 ± 1.52 (BSD), 3.28 ± 1.75 (DSD) C-peptide (μg/day) 74 ± 38 (C), 86 ± 40 (BSD), 75 ± 42 (DSD) iAUC_Ins_ (μU/mLx 2 h) after L 211 ± 74 (BSD), 144 ± 74 (DSD) iAUC_Glu_ (mg/dLx 2 h) after L 114 ± 41 (BSD), 62 ± 40 (DSD) HOMApp after L 59 ± 44 (BSD), 27 ± 23 (DSD)
Jakubowicz, et al., 2015 [296]	T2DM 57.8 ± 4.7 28.1 ± 2.9	5 mo, 14 exp. days Randomized open-label crossover-within-subject clinical trial	18	Bdiet (HE Breakfast) Ddiet (HE Dinner)	Bdiet: B—2946 kJ, 31% PRO, 47% CHO, 22% FAT L—2523 kJ, 27% PRO, 50% CHO, 23% FAT D—858 kJ, 43% PRO, 50% CHO, 23% FAT Ddiet: B—858 kJ, 43% PRO, 50% CHO, 23% FAT L—2523 kJ, 27% PRO, 50% CHO, 23% FAT D—2946 kJ, 31% PRO, 47% CHO, 22% FAT Composition of diets: 6276 ± 105 kJ 31% PRO 46% CHO 23% FAT B at ~08:00 h L at ~13:30 h D at ~19:30 h	−20% total day AUC_Glu_ for Bdiet vs Ddiet +20% total day AUC_Ins_ for Bdiet vs Ddiet +10% total day integrated AUC_Ins_ for Bdiet vs Ddiet −24% peak Glu (mmol/L × min) at 180 min after HE B vs HE D +10–19% peak Ins (pmol/L × min) at 30–180 min after HE B vs HE D Faster Ins peak (60 min) after B in Bdiet +17% C-peptide (nmol/L × min) after HE B vs HE D +35% tGLP-1 (pmol/L × min) at 30 min after HE B vs HE D 27% iGLP-1 (pmol/L × min) at 30 min after HE B vs HE D −13–25% Glu (mmol/L × min) after L in Bdiet vs Ddiet 50% higher, and more rapid early prandial Ins in Bdiet after L
Grant, et al., 2017 [308]	Healthy 25 ± 5.4 22.2 ± 1.6	4 days Controlled, parallel study	11	Eating at night group/condition (EN) Not eating at night group/condition (NoEN)	Meals at 19:00 h and 01:30 h Meals at 09:30 h, 14:10 h, and 19:00 h B was the tolerance test: ↑CHO (↑GI) at 06:30–07:00 h Tolerance test and measurement were done on 4 different days: PRE—day before the start of the protocol, SW4—days of stimulated nigh work (sleep between 10:00–16:00 h for all subjects), RTDS—return to daytime schedule	EN group: +27% AUC_Glu_ on SW4 +69% AUC_Glu_ on RTDS +11% AUC_Ins_ on SW4 +35% AUC_Ins_ on RTDS NoEN group: +12% AUC_Glu_ on SW4 +2% AUC_Glu_ on RTDS +18% AUC_Ins_ on SW4 +16% AUC_Ins_ on RTDS Fasting Glu: No significant effects of condition (*p* = 0.522), day (*p* = 0.539) or condition × day (*p* = 0.228) Fasting Ins: No significant effects of condition (*p* = 0.380), day (*p* = 0.056) or condition × day (*p* = 0.958) for fasting glucose and insulin, respectively
Lopez-Minguez, et al., 2018 [310]	Overweight/obese 42 ± 10 28.42 ± 4.04	2 exp. days, 1 week Randomized, cross-over trial	40	LE group (Delayed dinner or Late Eating) EE group (Advanced dinner or Early Eating) Also divided groups in MTNR1B risk carriers (GG) and non-risk carriers (CC)	LE: D at 23:00 h, L at 15:00 h, B at 8:00 h EE: D at 20:00 h, L at 12:00 h, B at 8:00 h Fixed menu for all meals on exp. days L was 8 h before D, with energy content of 650 Kcal D was 30–35% of total energy intake, composed of: 15–17% PRO 58–60% CHO/ 25–27% FAT	In total population: AUC_Glu_ (mmol/L x h) = 284.74 ± 32.67 (LE) vs 269.61 ± 34.8 (EE) Among GG group: AUC_Glu_ (mmol/L x h) = 292.2 ± 33.8 (LE) vs 270.9 ± 30.4 (EE) Among CC group: No significant effect AUC_Glu_ (mmol/L x h) = 277.3 ± 30.5 (LE) vs 268.2 ± 38.2 (EE) Significant interaction between meal timing (EE vs LE) and genotype (GG vs CC) for AUC_Glu_
Jakubowicz, et al., 2013 [317]	Overweight/obese 45.8 ± 7.1 32.4 ± 1.8	12 weeks Randomized open-label parallel-arm trial	74	B group D group	B group (700 kcal B, 500 kcal L, 200 kcal D) D group (200 kcal B, 500 kcal L, 700 kcal D) ~1400 kcal weight loss diets, same macronutrient content and composition B at 6:00–9:00 h, L at 12:00–15:00 h, D at 18:00–21:00 h	Fasting Glu −11.5% (B) vs −4.2% (D) Fasting Ins −51% (B) vs −29% (D) HOMA-IR −57% (B) vs −32.5% (D) HOMA-B −25% (B) vs −17% (D) ISI +163% (B) vs +56% (D) AUC_Glu_ −22% (B) vs −15% (D) AUC_Ins_ −58% (B) vs −30% (D)

Abbreviations: T2DM: type 2 diabetes; B: Breakfast; L: Lunch; D: Dinner; E: total energy intake; ppd: postprandial; GI: Glycemic Index; Glu: Glucose; Ins: Insulin; AUC: Area Under the Curve; iAUC: incremental area under the curve; CHO: carbohydrates; PRO: proteins; FAT: fats; HOMA: homeostatic model assessment for insulin resistance; ISI: insulin sensitivity index. An arrow pointing upwards indicates high carbohydrates and high GI.

In conclusion, the recommendations for persons wanting to lose weight may be that the fasting interval should be prolonged to 16 h, which prolongs the release of NEFAs and fat oxidation. A late large evening dinner shortens the fasting internal increasing the possibility of morning hyperglycemia. This is relevant also to a potential role of ketones produced during fasting in suppressing hunger and decrease food intake. The oxidation of fat and ketones may spare glucose to maintain a reasonable fasting level in blood to avoid metabolic alarm. More RCTs are needed in this field to identify the optimal meal and snack timing, whether this changes according to health status, such as in diabetes, and which are the exact mechanisms for amelioration of postprandial hyperglycemia and IR.

#### 4.2.3. Effects of Meal Frequency on Postprandial Glycemia and IR 

Several studies suggest that more frequent meals increase weight gain due to postprandial fat deposition [318,319], thereby worsening hyperglycemia, IR, hyperlipidemia, and appetite [319,320]. In contrast, others support the idea that frequent meals may reduce body weight and produce lower postprandial glycemic/insulinemic responses, lower blood lipids, improve metabolic control, and reduce appetite [321,322]. Those favoring many (5–6) regular smaller meals/day support that this dietary behavior diminishes glucose fluctuations/swings and provides a steadier delivery of nutrients throughout the day, thereby inducing lower glycemic loads and delayed gastric emptying resulting in less insulin requirements for glucose control, decrease in postprandial glucose levels, and in some cases reduction of hunger and the desire to eat [322,323,324,325]. Those favoring few (2–3) regular larger meals per day support that this dietary habit is more in line with human’s natural inclination to eat more in the morning and fast in the evening and during the night, and that it improves the expression of biological clocks regulating glucose metabolism and body weight [320,326]. In addition, some claim that having more meals per day can increase the consumption of unhealthier foods and added sugars leading to adverse effects in body weight, glycemia, and lipidemia [327]. Table 4 and Figure 3 describe the effects of meal frequency and energy and macronutrient distribution on postprandial hyperglycemia.

Clinical trials, both short-term (14–28 days) [321,325,328,329,330,331,332] and long-term [323,324] with no caloric restriction in non-obese and obese women with PCO syndrome at an early or late stage IGT or overt early-stage T2DM, and long-term studies with caloric restriction in T2DM without [333] or with antidiabetic treatment [320,326], have produced contradictory results regarding the association of meal frequency with body weight, postprandial glycemia, IR and overall glycemic control. Out of the only four long-term clinical trials investigating the effects of meal frequency in T2DM, the three with caloric restrictive diets provided contradictory results: (a) in T2DM subjects receiving anti-diabetic medications, 2 large vs 6 smaller meals decreased body weight, fasting plasma glucose/C-peptide/glucagon levels, with no differences in HbA1c, insulin, insulin sensitivity and blood lipids [320]. (b) In contrast, another study in T2DM reported that 5 vs 3 meals per day resulted in decreased BMI and HbA1c, without significant differences in fasting plasma glucose, insulin, and lipids [333]. (c) In uncontrolled subjects with diabetes treated with insulin, 3 vs 6 meals per day resulted in decreased body weight, HbA1c, total daily insulin requirements and higher expression of clock genes [326]. However, it is well known that both weight loss and antidiabetic medications have a significant impact on glucose/lipid metabolism [334], making it difficult to determine whether the beneficial effects were attributed to the medications, energy deficit or meal frequency.

**Table 4:** Effect of Meal, energy, and nutrient distribution in a day on glycemic control in individuals at high risk for developing or with type 2 diabetes.

Study	Age (Years) BMI (kg/m^2^) Health Status	Duration and Design of Dietary Intervention	Sample Size	Description of Groups	Dietary Intervention	Selected Clinical Outcomes
Effect of the Number of meals per day on Glycemia						
Kahleova, et al., 2014 [320]	T2DM 59.4 ± 7.0 32.6 ± 4.9	24 weeks each regiment Randomized, open, crossover study	54	A6 regiment (6 meals/day) B2 regiment (2 meals/day)	B, L, D, and 3 smaller snacks in between B and L 12 weeks per regiment and then crossover to other regiment for 12 weeks Caloric restriction of 500 kcal/day 50–55% CHO, 20–25% PRO, <30% FAT (≤7% SFAs, <200 mg/day of Chol), and 30–40 g/day of fibers	BW (signif.) −2.3 kg in A6 −3.7 kg in B2 HbA1c −0.23% in A6 −0.25% in B2 Fasting plasma Glu (mmol/L) −0.47 in A4 −0.78 in B2 Fasting immunoreactive Ins (pmol/L) −0.69 in A6 −0.75 in B2 Ins secretion at reference level (pmol min^−1^ m^−2^) +22.9 in A6 +20 in B2 Glu sensitivity (pmol min^−1^ m^−2^ mmol^−1^ L^−1^) +5.8 in A6 +5.9 in B2 TG (mmol/L) −0.28 in A6 −0.17 in B2 (all above changes per group were significant)
Papakonstantinou, et al., 2018 [324]	2 IGT groups (early and advanced stage) and T2DM 49.3 ± 1.8 32.4 ± 0.8	24 weeks Randomized, crossover study	47	IGT-A (PG 140–199 mg/dL at 120 min post OGTT) IGT-B (PG levels 140–199 mg/dL at 120 min and >200 mg/dL at 30, 60 or 90 min post-OGTT) T2DM (newly diagnosed treatment-naive T2DM)	Weight maintenance diet: 1900 kcal/day, 45% CHO, 20% PRO, 35% FAT 6 meals/day (B, L, D, and 3 snacks; with CHO: 20% at B, 10% morning snack, 30% at L, 10% at afternoon snack, 20% at D and 10% at bedtime snack) Or 3 meals/day (B, L, and D; with CHO: 20% at B, 50% at L and 30% at D) 12 weeks per regiment and then crossover to other regiment for 12 weeks	T2D group: ↓↓ post-OGTT Glu and ↓↓ HbA1c with 6 meals IGT-A group: ↓ 30-min and ↓↓ 60-min post-OGTT plasma Ins with 6 meals IGT-B group: ↓ peak Glu with 3 meals ↓↓ peak Glu with 6 meals In all groups: ↓ subjective hunger with 6 meals no differences in satiety or lipids no differences in FPG, Glu or Ins iAUC, fasting Ins, HOMA-IR with 3 vs 6 meals
Jakubowicz et al., 2019 [326]	T2DM ≥25 years 32.4 ± 5.2 HbA1c: 8.1 ± 1.1% T2DM for ≥5 yrs, treated with insulin ≥1 yr with >25 units for at least 3 months	3 months Randomized, parallel, treated with insulin, continuous glucose monitoring	28	6 meals 3 meals	Isoenergetic diets consisting of 3 or 6 meals/day: 3M 700 kcal breakfast, 600 kcal lunch, 200 kcal dinner; 6M same as 3M and addition of 150 kcal snacks	12 weeks with 3 meals/day vs 6 meals/day −5.4 kg weight loss, −1.2% total insulin dose—26 units, higher clock gene expression
Arnold, et al., 1997 [331]	T2DM or IGT 46–70 29.9±4.2	8 weeks Randomized, crossover study	13	3 meal regimes 9 meal regimes	Isoenergetic diets consisting of 3 or 9 meals/day 4 weeks with 3 meals/day; Daily E needs: 25% at B, 25% at L, ~50% at D, and ~150 kcal at a snack 4 weeks with 9 meals/day; Daily E needs: 8.3% at early morning, 8.3% at B, 8.3% at mid-morning, 8.3% at L, 8.3% at mid-afternoon, 8.3% at late-afternoon, 8.3% at D, 16.6% at mid-evening, and 16.6% at late evening; Meals were 1–2 h apart	Glu (mmol/L) +2% with 3 meals +4% with 9 meals Ins (μU/mL) +1% with 3 meals −2%with 9 meals Total Chol (mmol/L) +3.5% with 3 and with 9 meals TG (mmol/L) −13.5% with 3 and with 9 meals ApoB (mg/dL) +12% with 3 meals 17% with 9 meals
Salehi, et al., 2014 [333]	T2DM 35–65 n/a	3 months RCT	66	6-meal group (6 M) Control group (5 meals—usual pattern)	Weight loss diets (−300 kcal/day) 6 isocaloric meals 3 large meals and 2 small snacks (Ctrl) 56% CHO, 16% PRO and 28% FAT	↓↓ BMI in 6 M ↓ BMI in usual pattern ↓↓ HbA1c in 6 M ↓↓ Ins and 2 h-ppd Glu in 6 M ↓↓ Ins in usual pattern no sign. differences in fasting Glu, fasting Ins, and 2 h-ppd serum Glu in both groups
Effect of the Distribution of Energy and Macronutrients in a day on Glycaemia						
Pearce, et al., 2008 [82]	T2DM 61.3 ± 10 34.7 ± 9	3 days Randomized crossover study	23	CARB-E CARB-B CARB-L CARB-D	Even CHO distribution is all meals/day ~70 g CHO CHO mainly in B (~125 g) CHO mainly in L (~125 g) CHO mainly in D (~125 g) 40% CHO, 34% PRO, 26% FAT	Glucose max (mmol/L): 14.2 ± 1.0 CARB-L 14.5 ± 0.9 CARB-E 14.6 ± 0.8 CARB-D 16.5 ± 0.8 CARB-B Glu AUC_20_ (mmol/L·20 h): 10,049 ± 718 CARB-L 10,493 ± 706 CARB-E 10,717 ± 638 CARB-D 10,603 ± 642 CARB-B small but no sig. difference in fasting blood Glu
Jakubowicz, et al., 2015 [301]	T2DM 57.8 ± 4.7 28.1 ± 2.9	5 mo, 14 exp. days Randomized open-label crossover-within-subject clinical trial	18	Bdiet (HE breakfast) Ddiet (HE diner)	Bdiet: B—2946 kJ, 31% PRO, 47% CHO, 22% FAT L—2523 kJ, 27% PRO, 50% CHO, 23% FAT D—858 kJ, 43% PRO, 50% CHO, 23% FAT Ddiet: B—858 kJ, 43% PRO, 50% CHO, 23% FAT L—2523 kJ, 27% PRO, 50% CHO, 23% FAT D—2946 kJ, 31% PRO, 47% CHO, 22% FAT 6276 ± 105 kJ 31% PRO 46% CHO 23% FAT B at 08:00 h L at 13:00 h D at 19:00 h	−20% daily AUC_Glu_ for Bdiet −24% AUC_Glu_ in Bdiet after B Faster plasma Glu level decrease after B +11% AUC_Ins_ in Bdiet after B +12% Ins peak after B in Bdiet Faster Ins peak (60 min) after B in Bdiet −21–25% AUC_Glu_ in Bdiet after L +23% AUC_Ins_ in Bdiet after L 50% higher, and more rapid early prandial Ins in Bdiet after L
Imai, et al., 2018 [313]	T2DM 67.4 ± 9.4 23.5 ± 3.1	4 days Randomized, crossover clinical trial	17	Group 1: Day 2 snack at 12:30 h and day 3 snack at 15:30 h Group 2: Day 2 snack at 15:30 h and day 3 snack at 12:30	Diet: Same meals in the 2 groups with B at 07:00 h, L at 12:00 h, D at 19:00 h and snack at either 12:30 h (just after lunch—early) or at 15:30 h (mid-afternoon-late)	MAGE (mmol/L) 6.90 ± 0.69 with early snack 5.19 ± 0.48 with late snack iAUC (mmol/L per min^−1^) 1030 ± 180 with early snack 701 ± 97 with late snack Time of snack did not affect the mean Glu level
Kessler, et al., 2017 [314]	NGT and IGT 45.9 ± 2.5 27.1 ± 0.8	8 weeks Crossover trial	29	HC/HF HF/HC	Isocaloric diets, with energy intake equally distributed in the day HC/HF for 4 weeks: CHO-rich meals until 13:30 h, and FAT-rich meals 16:30 h–22:00 h HF/HC for 4 weeks: FAT-rich meals until 13:30 h, and CHO-rich meals 16:30 h–22:00 h	In subjects with impaired Glu tolerance and fasting Glu: Fasting Glucose −11.4% in HC/HF −9.6% in HF/HC Fasting Insulin −21.9% in HC/HF −27.1% in HF/HC Fasting C-peptide −42.6% in HC/HF −50.6% in HF/HC HOMA-IR −33.8% in HC/HF −34.7% in HF/HC Fasting GLP-1 −45% in HC/HF −13.3% in HF/HC Whole day Glu +7.9% in HF/HC vs HC/HF
Gibbs, et al., 2014 [315]	Healthy 25.5 ± 8.8 21.9 ± 1.7	4 exp. days Randomized, crossover study	10	Low GI meals (LG) High GI meals (HG)	LG: GI~37 HG: GI~73 LG and HG were consumed at (am) 8:00 h and (pm) 20:00 h	Glu peak (mmol/L) 7.8 ± 0.4 with LG & HG am 8.3 ± 0.2 with LG pm 9.54 ± 0.4 with HG pm Glu 2 h-ppd (mmol/L) Glu peak (mmol/L) 4.85 ± 0.2 with LG & HG am 6.42 ± 0.4 with LG & HG am no sig. differences in iAUC_Glu_ but a trend for ↓ iAUC_Glu_ with am meals no sig. differences in iAUC_Ins_

Abbreviations: IGT: impaired glucose tolerance; T2DM: type 2 diabetes; NGT: normal glucose tolerance; B: Breakfast; L: Lunch; D: Dinner; E: total energy intake; ppd: postprandial; GI: Glycemic Index, Glu: Glucose, Ins: Insulin, AUC: Area Under the Curve, iAUC: incremental area under the curve; CHO: carbohydrates; PRO: proteins; FAT: fats; M: meals; FPG: fasting plasma glucose; BW: body weight; RCT: randomized controlled clinical trial; HC: high carbohydrate; HF: high fat; HOMA: homeostatic model assessment for insulin resistance. Arrows pointing downward indicate a decrease. Two arrows pointing downward indicate a large decrease in the variables under investigation.

To address these issues, we conducted a long-term crossover study in subjects with early and late-stage IGT and naïve T2DM, who received weight maintenance diets as 6 and 3 meals per day [324]. We demonstrated that 6 vs 3 meals per day improved glycemic control in obese naïve T2DM subjects, resulting in significant reductions in HbA1c, peak glucose, postprandial glycemia and hunger, and stabilized or at least improved glycemic excursions reducing hunger and desire to eat in the subjects with IGT [324].

In conclusion, although there is evidence suggesting benefit from many regular smaller meals on glucose metabolism, it remains a controversial issue and more long-term studies are needed to make an evidence-based recommendation to people with IGT or T2DM.

### 4.3. Effects of Intermittent Fasting on Postprandial Glycemia

Intermittent fasting (IF) is characterized by interchanging between periods of fasting and feeding (Figure 3). Fasting periods last longer than the typical whole night fasting of 8–12 h. The exact hours of the eating window can vary with eating occurring in the morning hours (i.e., 09:00–15:00, 09:00–13:00, etc.), in the middle of the day (i.e., 12:00–17:00, etc.), or extending later to night hours (i.e., 12:00–21:00, 13:00–18:00, etc.). Studies in mice have reported beneficial effects of IF against diabetes and obesity with improvements in glucose tolerance and insulin sensitivity, maintenance of insulinemia within normal levels, and improvements in the phosphorylated cyclic AMP response element-binding protein (CREB), rapamycin complex (mTOR), and AMP-activated protein kinase (AMPK) pathways, even in mice fed high-fat diets [256,335,336,337]. The concept behind IF is that long-term fasting diets can also lead to ketosis, during which glucose reserves run out and glycogen stores are insufficient to provide energy to the brain and the CNS, using ketone bodies and acetone as alternative fuels [338]. Ketosis has been proposed by some to positively affect metabolism with a reduction in ROS, lipolysis, autophagy, and increases in stress resistance, among others [339], further extending the effects of caloric restriction. Table 5 describes the effects of eating windows in a day on postprandial hyperglycemia.

There are various types of IF protocols all of which divide the day or week in periods of feeding and fasting achieving energy restriction. In most cases there is no guidance for which foods to consume, and when to consume them. IF can be grouped into alternate-day fasting (ADF) (up to 75% energy restriction), whole-day fasting and time-restricted feeding (TRF), whereas in the weekly restricted feeding protocols energy intake is restricted from 1–3 days in a week up to 5 days in a month with ad libitum intake in the remaining days (i.e., 5:2 diet) [338,340,341].

Few TRF trials have been conducted in humans, and a small number of those focused on the effect of TRF patterns in people at risk for developing or with diagnosed T2DM (Table 5). Two short-term (2-week) trials compared the effects of a 9–10 h early or late eating window and reported that early-eating improved BP and glucose tolerance (decreased blood glucose areas under the curve, mean fasting and postprandial glucose levels), while late-eating increased plasma glucose, insulin, and triglyceride levels during the night [342,343]. A study of 11 weeks duration in which overweight participants followed a 6-h early TRF plan (08:00–14:00) resulted in a decrease in night glycemia, 24-h blood glucose fluctuations, fasting plasma glucose/insulin levels, HOMA-IR, and increased evening plasma insulin levels, without changes in day plasma glucose concentrations [344]. Patients with metabolic syndrome following a 10-h TRF also demonstrated reductions in body weight and improvements in glycemia (reduced HbA1c, plasma glucose/insulin levels and HOMA-IR) [345].

**Table 5:** Effect of the eating window in a day on Glycemia in healthy individuals and subjects at high risk for developing or with type 2 diabetes.

Study	Health Status Age (Years) BMI (kg/m^2^)	Duration and Design of Dietary Intervention	Sample Size	Description of Groups	Dietary Intervention	Selected Clinical Outcomes
Effect of Time of Feeding on Glycemia						
Feeding/Fasting						
Carter, et al., 2018 [346]	T2DM, overweight/obese 60.5 ± 9.2 36.0 ± 5.8	12 months Parallel randomized clinical trial	97	Intermittent energy restriction group (IER) Continuous energy restriction group (CER)	IER: 500–600 kcal/day for 2 non-consecutive days of the week, usual diet for the other 5 days CER: 1200–1500 kcal/day Similar weekly energy restrictions	BW −5 kg in CER −6.8 kg in IER HbA1c −0.5% in CER −0.2% in IER Ins −0.3% in CER −1.2% in IER
Carter, et al., 2019 [347]	T2DM, overweight/obese 62 ± 9 35 ± 4.8	12 months—24-month follow-upParallel randomized clinical trial	84	Intermittent energy restriction group (IER) Continuous energy restriction group (CER)	IER: 500–600kcal/day for 2 non-consecutive days of the week, usual diet for the other 5 days CER: 1200–1500kcal/day 24 months Follow-up	At 24 mo.: BW −3.9 kg in CER −3.9 kg in IER HbA1c +0.4% in CER +0.1% in IER TG −0.2 ± 0.3 mmol/L in CER −0.02 ± 0.2 mmol/L in IER
Varady, et al., 2013 [348]	Normal BW/overweight) 47.5 ± 2.5 2 ± 1	12 weeks Randomized, controlled, parallel-arm feeding trial	30	ADF Control (ctrl)	ADF: 25% of E needs on fast day (~400–600 kcal at 12:00 h–14:00 h), and ad libitum eating on each alternating feed day ad libitum eating every day	↓ BW in ADF −13% total Chol in ADF −20% TG in ADF −5% BP (systolic) in ADF −50% CRP in ADF
Catenacci, et al., 2016 [349]	Obese 41.15 ± 8.7 37.65 ± 4.85	8-week intervention & 24 weeks unsupervised follow-up Randomized trial	26	CR (caloric restriction) ADF (alternate day fasting)	CR: −400 kcal/day less than E needs ADF: ad libitum food in fed days, only water, calorie-free beverages and bouillon/stock cube soup on fast (0 kcal) days E distribution in both groups: 20% B, 30% L, 40% D and 10% snack	BW −7.1 kg in CR at week 8 −8.2 kg in ADF at week 8 −5 kg in CR at week 12 −5.7 kg in ADF at week 12 Glu (mg/dL) +3.3 in CR at week 8 +6 in ADF at week 8 +1.7 in CR at week 12 +2.6 in ADF at week 12 Ins (μU/mL) −0.2 in CR at week 8 +3 in ADF at week 8 −2 in CR at week 12 +0.4 in ADF at week 12 TG (mg/dL) −2.8 in CR at week 8 −25 in ADF at week 8 +12 in CR at week 12 +5.1 in ADF at week 12
Trepanowski, et al., 2017 [350]	Obese 44 ± 11 34 ± 4	12 months (6 mo. weight loss & 6 mo. maintenance) RCT	69	Alternate day fasting group (ADF) Daily calorie restriction group (DCR) Control—No intervention group (ctrl)	ADF: 25% of energy needs on fast days; 125% of energy needs on alternating “feast days” DCR: calorie restriction to 75% of energy needs every day Ctrl: no-intervention control	ADF vs ctrl: At 6 months −6.8% BW −6.3 mg/dL Glu −7.5 μIU/mL Ins −2.49 HOMA-IR At 12 months −6% BW −3.9 mg/dL Glu −5.9 μIU/mL Ins −1.86 HOMA-IR DCR vs ctrl: At 6 months −6.8% BW −4.9 mg/dL Glu −7.0 μIU/mL Ins −2.56 HOMA-IR At 12 months −5.3% BW −9.6 mg/dL Glu −4.6 μIU/mL Ins −1.88 HOMA-IR
Gabel, et al., 2019 [351]	Insulin resistant subjects 42 ± 3 y.o. 35 ± 1	12 months (6 mo. weight loss & 6 mo. maintenance) RCT	100 (43 completed)	Alternate-day fasting group (ADF) Daily calorie Restriction group (CR) Control group (ctrl)	6-months reduced net E intake by 25% Fasting days: 25% of E needs at lunch (12:00 h–14:00 h) Alternating feast days: 125% of E needs over 3 meals/day 6-mo. reduced net E intake by 25% per day over 3 meals every day 6-mo. weight maintenance phase: Both for ADF & CR groups → ADF consumed 50% of E needs on fast days and 150% on feast days → CR consumed 100% of E needs/day Instructed to maintain body weight	↓ weight (~18% for ADF, and ~14% for CR) Glu (mg/dL): no significant changes Ins (μIU/mL): −44% ADF 6 mo, −52% ADF 12 mo, −23% CR 6 mo, −14% CR 12 mo HOMA-IR: −48% ADF 6 mo, −54% ADF 12 mo, −19% CR 6 mo, −17% CR 12 mo
Hoddy, et al., 2014 [352]	Obese 45.5 ± 2.5 34.5 ± 1	8 weeks Randomized, parallel-arm feeding trial	59	ADF-L Alternate day fasting—intake at lunch ADF-D Alternate day fasting—intake at dinner ADF-SM Alternate day fasting—intake in small meals	25% of E needs on fast day and ad libitum eating on feed day ADF-L: One meal (L) at 12.00 h–14.00 h on each fast day ADF-D: One meal (D) at 18.00 h–20.00 h on each fast day ADF-SM: divided their fast day meal in 3 mini meals → 100 kcal at 6:00 h–8:00, 300 kcal at 12:00 h–14:00 h and 100 kcal at 18:00 h–20:00 on each fast day	BW −3.5 kg in ADF-L −4.1 kg in ADF-D −4.0 kg in ADF-SM Glu −2% in ADF-L −1% in ADF-D −1% in ADF-SM Ins no change in ADF-L −18% in ADF-D −12% in ADF-SM HOMA-IR −10% in ADF-L −27% in ADF-D −19% in ADF-SM TG −6% in ADF-L −8% in ADF-D −1% in ADF-SM
Harvie, et al., 2011 [353]	Overweight or obese 40 ± 4 30.6 ± 5.1	6 months Randomized trial	89	Continuous energy restriction group (CER) Intermittent energy restriction group (IER)	CER: 25% restriction below estimated requirements for 7 days per week IER: 75% restriction for 2 days per week, with no restriction on the other 5 days per week 25% PRO, 45% low GL CHO, 30% FAT (15% MUFAs, 7% SFAs, 7% PUFAs)	BW −5% in CER −7% in IER Ins −15% in CER −29% in IER HOMA −19% in CER −27% in IER Glu −2% in CER −2% in IER Adiponectin no change in CER +10% in IER Ghrelin +11% in CER +13% in IER TG −23% in CER −17% in IER BP (systolic) −6% in CER −3% in IER
Harvie, et al., 2013 [354]	Overweight 47.4 ± 7.7 30.9 ± 5.1	4 months (3 months weight loss and 1 month maintenance) Parallel randomized clinical trial	115	IECR: energy and CHO restriction IECR + PF: allowed ad libitum PRO and FAT DER: daily energy restriction	IECR: 25% overall E restriction, And for 2 d/week restricted CHO (<40 g/day), On restricted days: 20% CHO, 45% PRO and 35% FAT IECR + PF: 25% overall E restriction, And for 2 d/week restricted CHO (<40 g/day) and ad libitum PRO and FAT (MUFA and PUFA), On restricted days: 15% CHO, 35% PRO and 50% FAT DER: 25% overall E restriction, 45–50% CHO, 20–25% PRO and 30% FAT	BW −5.5 kg in IECR −5.1 kg in IECR + PF −3.8 kg in DER Ins −21% in IECR −11% in IECR + PF −10% in DER HOMA-IR −25% in IECR −16% in IECR + PF −11% in DER HbA1c no change in HbA1c in all groups Glu no change in Glu in all groups TG −9% in IECR −14% in IECR + PF −7% in DER BP (systolic) −3% in IECR −13% in IECR + PF −9% in DER
Time restricted Feeding (TRF)						
Hutchison, et al., 2019 [342]	Overweight/Obese 55 ± 3 33.9 ± 0.8	14 exp. days Randomized crossover trial	15	TRFe TRFd	2 × 7 days separated by a 2-week washout Eating window: 8:00 h–17:00 h Eating window: 12:00 h–21:00 h Ad libidum water and very low-calorie (<4 kcal/serving) drinks and foods	↓ BW on day 7 iAUC_Glu_ −36% in TRFe −21% in TRFd No effect on fasting Glu, Ins, (but a ↓ trend) ↓ mean fasting Glu for TRFe vs baseline ↓ 3 h ppd Glu for TRFe vs baseline
Wehrens, et al., 2017 [343]	Normal BW/Overweight 18–30 20–30	13 exp. Days Human laboratory trial	10	One group	Sleeping schedule: ~ 23:00–6:30 h Day 1–3: Wake up at 6:30 h, B at 7:00 h, L at 12:00 h, D at 17:00 h Day 4–5: No treatment, measurements Day 6–11: Wake up at 6:30 h, B at 12:00 h, L at 17:00 h, D at 22:00 h Day 12–13: No treatment, measurements Isocaloric meals: 55% CHO, 15% PRO, 30% FAT	Late meals resulted in: ↑ 5.69 h Glu acrophase (into sleeping time) ↑ 1.5 h Ins acrophase ↑ 1 h TG acrophase
Jamshed, et al., 2019 [344]	Overweight 32 ± 7 30.1 ± 2.7	8 days Randomized controlled crossover study	11	eTRF Control (ctrl)	6 h feeding/18 h fasting Feeding: 8:00 h to 14:00 h B at 8:00 h, L at 11:00 h, and D at 14:00 h 12 h feeding/12 h fasting Feeding: 8:00 h to 20:00 h B at 8:00 h, L at 14:00 h, and D at 20:00 h 3 daily meals were matched: 15% PRO, 50% CHO, 35% FAT 4 days in each condition—3.5–5 weeks washout—4 days in other condition	No significant changes in day Glu −7 ± 2 mg/dL night/sleep Glu in eTRF −4 ± 1 mg/dL 24-h blood Glu in eTRF −12 ± 3 mg/dL MAGE in eTRF −2 ± 1 mg/dL morning fasting Glu in eTRF −2.9 ± 0.4 mU/L morning fasting Ins in eTRF −0.73 ± 0.11 HOMA-IR in eTRF +25 ± 9% IRS2 gene expression in eTRF +4.5 ± 1.6 mU/L evening fasting Ins in eTRF +1.09 ± 0.43 evening HOMA-IR in eTRF No changes in GLUT1, GLUT4, or IRS1 gene expression at either time of day
Wilkinson, et al., 2020 [345]	Metabolic syndrome patients 59 ± 11.14 33.06 ± 4.76	12 weeks Single-arm, paired-sample trial	19	TRF	10 h feeding/14 h nightly fasting Eating at will, subjects reported foods consumed via smartphone app (food and time logs)	TRF vs baseline: −3% BW −5% Blood Glu (CGM) −5% Fasting blood Glu −2% HbA1c −21% Fasting Ins −30% HOMA-IR
Sutton, et al., 2018 [355]	Overweight with IGT 56 ± 9 32.2 ± 4.4	5 weeks Randomized, crossover trial (controlled feeding)	8	eTRF Control (ctrl)	eTRF: 6 h eating window/day, 3 meals, dinner before 15:00 h, ~18 h fasting Ctrl: 12 h eating period/day, 3 meals, ~12 h fasting between 20:00 h–6:30 h Each plan for 5 weeks, with a 7-week washout period Isocaloric and eucaloric controlled feeding diets (BW maintenance) 15% PRO, 50% CHO, and 35% FAT	No weight loss Fasting Glu no significant changes Mean Glu no significant changes Fasting Ins −3.4 ± 1.6 mU/L Mean Ins −26 ± 9 mU/L Peak Ins −35 ± 13 mU/L Insulinogenic index +14 ± 7 mu/mg IR (iAUC) −36 ± 10 mU/mg
Parr, et al., 2020 [356]	T2DM 50 ± 8.9 34 ± 4.8	6 weeks Pre-post non-randomized	19	Habitual diet (2 weeks) TRF (2 weeks)	Habitual diet: ~8400 kJ/day; 35% CHO, 20% PRO, 41% FAT, 1% alcohol 9 h feeding/15 h fasting TRE diet: ~8500 kJ/day; 35% CHO, 19% PRO, 42% FAT, 1% alcohol Eating at will	No weight loss −3% HbA1c −3.6% Glu +18% Ins
Moro, et al., 2016 [357]	Healthy athletes 29.21 ± 3.8 BW 84.6 ± 6.2 Kg	8 weeks Randomized, single blind trial	34	TRF Normal Diet group (ND)	8 h feeding/16 h fasting Meals: 13:00 h (40% of total cal), 16:00 h (25% of total cal), 20:00 h (35% of total cal) Meals: 8:00 h (25% of total cal), 13:00 h (40% of total cal), 20:00 h (35% of total cal)	↓ in fat mass −16.4% in TRF −2.8% in ND Glu −11% in TRF no change in ND Ins −36.3% in TRF −13% in ND

Abbreviations: B:Breakfast; L: Lunch; D: Dinner; E: energy; ppd: postprandial, Abbreviations: T2DM: type 2 diabetes; IGT: impaired glucose tolerance; B:Breakfast; L: Lunch; D: Dinner; E: energy; ppd: postprandial, GI: Glycemic Index; GL: Glycemic Load; Glu: Glucose; Ins: Insulin; AUC: Area Under the Curve; iAUC: incremental area under the curve; CHO: carbohydrates; PRO: proteins; FAT: fats; TRF: time-restricted feeding; CGM: continuous glucose monitoring; BW: body weight; RCT: randomized controlled clinical trial; HOMA: homeostatic model assessment for insulin resistance; TG: triglycerides; BP: blood pressure. Arrows pointing upwards or downwards indicate an increase or decrease.

Improved insulinemia was observed in overweight subjects with IGT who followed a 6-h TRF, but in this study significant changes in fasting or mean glycemia were not observed [355]. T2DM subjects, who followed a 2-week TRF plan with a 9-h eating window per day demonstrated reductions in hyperglycemia and HbA1c, and increases in plasma insulin levels, even though there was no weight loss observed [356]. TRF’s favorable glycemic and insulinemic effects were reported in healthy athletes performing resistance training [357]. An isocaloric study showed that more body fat was lost with 6-h vs 12-h feeding with significant differences in weight loss (1.5 kg), plasma adiponectin, testosterone, interleukin-1β, and IGF-1 levels, but no changes in fat free mass, resting energy expenditure, and plasma thyroid hormone/glucose levels [357]. A study demonstrated that after one year of following IF (2/5 days per week consuming 500–600 kcal) the weight loss achieved was accompanied by reduction in insulinemia and HbA1c [346]. Weight loss and decreased insulin responses were also observed in a group that received a caloric restricted diet, but these reductions were smaller than those of the IF group; two years after that intervention, both groups had regained some of the weight, HbA1c was lower in the IF group, and both groups now presented a reduction in plasma TG levels [347].

Smaller duration studies from 8 to 12 weeks examining ADF have shown that it is a safe and tolerable approach to weight loss, although it provided conflicting evidence regarding markers for CVD protection [348,349]. In a 12-month study, ADF (25% of daily energy needs on fasting days: one day on, one day off) was compared with a calorie-restricted (75% of energy needs every day) and a control diet [350]. It was demonstrated that although the two intervention groups lost weight, insulin sensitivity, blood lipid levels and blood glucose were not improved. Analysis of insulin-resistant participants from that study, revealed similar weight loss between IF and calorie-restricted interventions after 12 months [351]. Both diet interventions had similar effects on reductions in fat mass and BMI. However, ADF resulted in greater decreases in fasting insulin and insulin sensitivity despite a similar decrease in body weight with the other diet plans [351]. In contrast, another study showed that allowing people to consume the fast day meal at the evening dinner (or dividing it into smaller meals) produced similar weight loss and CVD protection as consuming the meal at lunch, thus allowing for more flexibility in meal timing and possible between long-term adherence to ADF protocols [352].

A 5-week crossover study in men with prediabetes showed the early-time IF (6 h feeding period, with dinner before 15:00) to be in alignment with circadian rhythms in metabolism compared to a control schedule (12 h feeding period), improved insulin sensitivity, β-cell responsiveness, BP, oxidative stress, and appetite, independently of weight loss [355]. Early vs delayed TRF improved glucose tolerance in men at risk for T2DM [342]. Greater weight loss was observed in early eaters (before 15:00) vs later eaters (after 15:00) [312]. Three isocaloric studies showed that front loading (high caloric intake at breakfast) vs evening dinner improved weight loss [317,320,358]. A TRF pattern, with a long fast (>12–14 h) and a limited eating window not extending late in the night, with ~4–10 h in which meals were distributed and consumed, seemed to have several health benefits independent of energy restriction [359]. It has been reported that ADF studies in normal weight, overweight, and obese subjects with a duration of 3–12 weeks were effective in achieving an approximately 3–7% weight loss, 3–5.5 kg body fat loss, lowering blood lipids (approximately 10–21% for total cholesterol and 14–42% for triglycerides) [360]. Whole day fasting studies with a duration of 12–24 weeks also achieved a 3–9% body weight loss, body fat loss and lowering of blood lipids (approximately 5–20% for total cholesterol and 17–50% for triglycerides) [360].

Many people have time-restricted windows, they do not eat their first meal before 14:00 and stop eating at 02:00. Feeding periods after 16:00 had either no effect or unfavorable effects on postprandial glycemia, β-cell responsiveness, BP, and blood lipids [360,361,362]. It has been shown that fasting until 12:00 results in increased postprandial hyperglycemia and impaired insulin responses after lunch and evening dinner in healthy and T2DM subjects [72], due to effects directly on clock genes and time-controlled genetic expression [285]. To examine whole week feeding restriction regimens, one study [363] randomized 54 obese individuals with T2DM into 3 groups: (a) a very low-calorie diet (VLCD) for 5 days and then 1 day/week VLCD for 15 weeks; (b) a VLCD for 5 days and then 5 days VLCD every 5 weeks consuming 1500–1800 kcal per day without fasting; and (c) a control group consuming 1500–1800 kcal per day for the duration of the study. This study showed that both VLCD groups lost significantly more body weight with the second group losing >5 kg [363]. Another study with 63 overweight and obese T2DM subjects randomized to consume 400–600 kcal/day 2 days/week and then, typical energy intake for 5 days/week vs whole day energy restriction to 1200–1550 kcal/day showed that after 12 weeks there were similar reductions in HbA1c, body weight, antidiabetic medications, body composition, and appetite in both groups [364]. The issues arising with IF is that there is a variety of definitions making hard to interpret the scientific results in a systematic manner: the number of fasting days and the energy intake vary in each study, the management of hunger during fasting days and the days of regular feeding are in most cases unknown, the probability of overconsumption in days of typical feeding is high and social life is affected, particularly in countries of Southern Europe (i.e., Italy, Greece, Spain, etc.) where eating late dinner after 19:00 is very common. Two studies showed that intermittent continuous energy restriction (IER) was a similarly effective alternative to continuous energy restriction (CER) regarding weight loss [353,354].

In conclusion, some practical strategies from IF trials that seem to be effective in body weight and postprandial glycemia support consuming the evening dinner earlier in the day, consuming most of the energy at breakfast and lunch, avoiding snacks late at night, and reducing feeding time by 1–2 h with the last meal before 18:00 or the latest at 20:00. Due to the variable IF protocols, the choice of IF pattern, the short duration of trials, the varied window of energy intake and the heterogeneous populations studied, more well-designed longer duration studies are needed to draw safe conclusions and recommendations.

## 5. Alternative Dietary Interventions and Postprandial Glycemia

Various plant-derived foods, plant compounds and functional ingredients/foods have been linked with ameliorated postprandial hyperglycemia, and they may be considered “alternative” dietary interventions for diabetes management.

### 5.1. Vitamins–Minerals 

Without underlying deficiency, the benefits of multivitamins or mineral supplements on glycemia for prediabetes or T2DM have not been supported by evidence, and therefore routine use is not recommended [114]. Accordingly, the routine use of chromium, vitamin D, micronutrient supplements for improving glycemia in people with diabetes is not supported by evidence and is also not recommended [114].

### 5.2. Herbs and Spices

Use of herbs and spices during cooking is encouraged as it is a safe method for flavoring and preservation of food, and for providing antioxidant ingredients, such as phenolic compounds. Cinnamon, one of the most used culinary spices, may be considered an alternative food intervention. It belongs to the *Cinnamomun* family and is found commercially in many forms including sticks of bark, powder, and powder-derived extracts. Cinnamon contains various bioactive compounds such as cinnamaldehyde, eugenol, trans-cinnamic acid, phenolic compounds, tannins, catechins, terpenes, proanthocyanidins, and coumarin. The content of these compounds differs according to the form in which cinnamon is used. Both in vitro and in vivo animal studies have shown insulin-enhancing or insulin-like effects after cinnamon administration [365,366,367]. Several clinical trials have been conducted since the 2000s that administered cinnamon as extracts, capsules or supplements at varying amounts ranging from 0.5 g to 6 g per day, in subjects with T2DM. Most of the studies have reported a generally favorable effect of cinnamon on postprandial/fasting hyperglycemia and diurnal glucose fluctuations [368,369,370,371,372,373,374,375]. It has been shown that even low amounts (1 g/day) of cinnamon powder was enough to reduce fasting glucose and improve blood lipid profiles of T2DM patients [375]. Higher doses also confirmed these effects in some studies [369,370,371,372,373,374]. Other studies reported additional improvements on insulin [369] and HbA1c [369,370,372,373] levels after cinnamon addition in the diet (as extracts or supplements). Glucose and insulin responses to oral glucose tolerance test (OGTT) were also found to be improved in healthy subjects after cinnamon supplementation (3 gr/day) [376]. However, not all studies agree with the favorable glycemic effects of cinnamon. Results from 6 RCTs did not find a difference in blood glucose or insulin levels after cinnamon consumption [368,377,378,379,380,381,382]. Decreased serum triglycerides, total cholesterol, and LDL-cholesterol were also demonstrated in a few reports [370,372,375]. Interestingly, one study reported that the positive effects of cinnamon consumption on glycemia are acute and do not persist after cessation [376]. Cinnamon intake tended to be beneficial for controlling glycemia in T2DM patients with poor glycemic control, but more research is needed to establish these effects. However, use of any herbal supplements, including cinnamon, curcumin, or aloe vera, for improving glycemia in people with diabetes is not supported by sufficient scientific evidence and is therefore not recommended [114].

### 5.3. Fermented Foods

Fermented foods and beverages, defined as “foods made through desired microbial growth and enzymatic conversions of food components”, have been used for thousands of years, and are proposed to offer several health benefits due to their bacteria strains, and the presence of certain organic acids in foods generated through fermentation or added, and are responsible for texture, flavour, and better preservation of fermented foods [383,384]. Lactic acid may lead to slower starch absorption due to inhibition of amylolytic enzymes, thus, reducing its bioavailability due to the interaction between starch and gluten, and the delay of gastric emptying, leading to lower postprandial glycemic responses [385,386]. Inclusion of the sodium salt of propionic acid to whole-meal barley bread has also been reported to lower postprandial hyperglycemia [383]. Sourdough fermentation of wheat dough has been repeatedly reported to lower the GI of bread and postprandial glucose excursions, due to the formation of organic acids, leading to reduced rate of starch digestion and delayed gastric emptying [383,385,386,387,388]. Results from one study reported that consumption of meals containing beta-glucan, fermented in the colon, resulted in decreased postprandial glucose concentrations due to a delayed and somewhat reduced carbohydrate absorption from the gut not from the effects of fermentation in the colon [389]. However, another study showed that sourdough fermentation is a method able to lower the postprandial glycemic responses to bread, but this did not relate to the starch accessibility or general bioavailability, but rather to bacteria-induced delayed gastric emptying [390]. The latter was also supported by another study reporting that the lactic acid in fermented milk products did not lower the postprandial glycemic responses and concluded that the presence of organic acids may counteract the insulinotropic effect of milk in mixed meals [391]. Additionally, it has also been reported that fermentation of carbohydrates may regulate postprandial glucose excursions to a second meal by reducing NEFA competition for glucose disposal and to a minor extent by affecting intestinal motility [392].

Vinegar (acetic acid) is an example of fermented food. Vinegar consumption dates back to ancient times with reports of its use by Hippocrates for wound care [393]. In folk medicine, vinegar is considered a natural remedy and is used as such, whereas now it has been included in the “super foods” for its properties and claims of effects on weight loss, digestion, and even skin quality. Most of trial data support beneficial effects of vinegar on postprandial glycemia and overall glycemic control. In healthy subjects, vinegar consumption with a meal (either as dressing on salad or as a drink) resulted in generally lower postprandial glycemia [386,394,395,396] and in some studies reduced insulinemia [386] as well. In one study, a 55% reduction in post-meal glycemia was reported when a high GL meal was consumed [397]. Another study suggested that the effects of vinegar consumption are prominent when ingested with carbohydrate-rich meals rather than with low GL meals [397]. A few clinical trials have also been conducted in individuals with prediabetes and/or T2DM. Vinegar intake before or together with a meal was associated with improved glycemia, reduced post-meal area under the curve for glucose, reduced fasting blood glucose, insulin and triglycerides, increased muscle glucose uptake, and reduced the need for fast-acting insulin in subjects with T1DM [398,399,400,401,402,403]. However, in another study, postprandial plasma glucose or insulin were unaffected by vinegar co-ingestion [404]. Currently, there is also some data explaining the possible mechanisms by which vinegar affects glycemia. Acetic acid consumption has been shown to delay gastric emptying in healthy subjects and in people with diabetes [386,405]. Additionally, digestion of complex carbohydrates is also delayed by inhibition of relevant enzymes such as sucrase and disaccharidase [406,407]. All these effects have been shown to reduce postprandial glucose responses. Vinegar intake at bedtime was associated with lower fasting glycemia by decreasing the rates of hepatic gluconeogenesis and improving insulin secretion in subjects with T2DM [401].

Yoghurt and cultured milk products are other examples of fermented foods and have been shown to reduce postprandial glycemic responses in both healthy individuals, and subjects with prediabetes or overt T2DM [384], beyond the milk from which these products are made [408]. Similar results have been reported in at least one RCT with milk kefir, kimchi, sauerkraut and natto [384]. A recent consensus statement on fermented foods from the International Scientific Association for Probiotics and Prebiotics reported that although the family of fermented foods is large (variable food categories including fermented dairy products and other fermented foods with living versus dead microorganisms; food types including fermented vegetables, fermented soy and yoghurt; and individual fermented food products with well-characterized strains and nutrient compositions), and not all foods are examined or proven for their health benefits, and the mechanisms by which they may lead to lower postprandial glycemic responses have not been fully elucidated, consumption of some of these food products seems promising and their beneficial health effects remain to be established by more and well-designed RCTs and may also be obtained from harvesting information from existing population-based diet and health databases [384]. In conclusion, consumption of fermented foods should be encouraged and some of these foods may lead to amelioration of postprandial hyperglycemia and IR.

### 5.4. Probiotic Dairy Foods

Another alternative dietary intervention includes probiotic dairy foods. A recent mini-review suggested that matured products (i.e., ripened cheeses), fermented dairy products (kefir), and whey-based products (mainly milk beverages), and the addition of prebiotics and/or plant-derived products have a higher ability to regulate postprandial glycemia due to their probiotic strains with higher proteolytic and exopolysaccharides-forming abilities leading to inhibition of digestive enzymes, such as the a-amylase (1,4-alpha-D-glucan-glucanohydrolase), the enzyme that hydrolyses polysaccharides to glucose and maltose oligosaccharides, and the α-glucosidases, membrane bound enzymes located in the epithelium of the brush borders of the small intestine, that hydrolyze the oligosaccharides at the non-reducing links releasing the bound α-glucose, thus increasing the blood glucose levels [409]. However, a recent systematic review with 27 probiotic interventions (Lactobacillus, Bifidobacterium, Clostridium and Akkermansia) reported contradictory results regarding the effects of certain probiotics on amelioration of IR, suggesting the need for long-term RCTs in people with obesity and cardiometabolic risk [410].

### 5.5. Other Alternative Dietary Interventions: i.e., Inulin, Polyphenols, Chia Seeds, Nuts and Whey Protein

Results from two systematic reviews and meta-analyses of 33 RCTs in healthy, overweight/obese, prediabetes, T2DM, and hyperlipidemic subjects examining the metabolic effects of inulin-type fructan intake, reported a reduction in blood glucose, total cholesterol, and triglyceride concentrations in people with diabetes, although the mechanisms were inconclusive without differences from controls on body weight and blood insulin and with data showing large heterogeneity [411,412].

A western diet is able to deliver between 109 and 313 mg of polyphenol per day, Mediterranean diet between 820 mg and 1.3 g per day, whereas red wine, the food with the highest resveratrol content, contains around 3 mg/100 mL [413]. Typical foods containing polyphenols include tea, coffee, chocolate, cocoa, cinnamon, grape, pomegranate, red wine, berries, and olive oil [414]. A systematic review and meta-analysis of 36 RCTs examining the effects of polyphenols (extracts, supplements, and foods), supplemented in doses of 28 mg to 1.5 g, for 0.7 to 12 months on HbA1c levels in healthy subjects and individuals with prediabetes or T2DM, reported significant reduction in HbA1c (~0.5%), in those with T2DM, without significant effects in the healthy subjects and those with prediabetes [413]. Polyphenol rich extracts had a more marked effect in reducing HbA1c among the trials; with 125 mg/day isoflavonoids from soy products, 1–3 f of cinnamon (27 mg/day of coumarin), 250 mg/day resveratrol from extracts, reported to have the highest efficiency in reducing HbA1c [413]. Dietary polyphenols’ beneficial reported effects on postprandial hyperglycemia and IR may be due to inhibition of α-amylase and α-glucosidases, inhibition of intestinal glucose absorption by sodium-dependent glucose transporter-1, stimulation of insulin secretion, and reduction of hepatic glucose output [414].

Chia (*Salvia hispanica L.*) seeds and oil, contain α-linolenic acid, vegetable protein and dietary fiber [415]. A systematic review and meta-analysis of 12 trials with healthy individuals, athletes, and subjects with T2DM or metabolic syndrome, showed reductions in postprandial blood glucose and HDL cholesterol levels, and BP, only at the high doses, and only in subgroup analysis with the effects being modest and probably not clinically significant, and with the quality of evidence of the studies being low or very low [415].

Although there is increased scientific interest in the metabolic effects of nuts, the family of nuts is heterogeneous, and not all nuts are reported to be beneficial. A systemic review and meta-analysis of 6 RCTs examining the effects of pistachio nuts on glycemic control and insulin sensitivity in people with T2DM, prediabetes, or metabolic syndrome, reported a significant reduction in fasting glucose and IR, without differences from controls on HbA1c and fasting plasma insulin; the reported beneficial effects were possibly related to their high content of antioxidants (beta-carotene, lutein, proanthocyanidins, and vitamin E), anti-inflammatory compounds, fiber, and healthy fats, monounsaturated fatty acids [416]. Pistachio nuts’ high content of antioxidant components has been proposed to be involved in their beneficial effects on insulin sensitivity [417]. A positive impact of a very high daily dose (85 g/day) of pistachio nuts on postprandial insulinemia has been reported, particularly when consumed with a high carbohydrate diet [418,419]. Also, their monounsaturated fatty acids have been suggested to reduce oxidative stress and improve the insulin-signaling pathway and IR, by maintaining membrane translocation of glucose transporters along with buffering β-cell hyperactivity [420,421]. Moreover, it has been suggested that consumption of high daily doses of pistachio nuts (>57 g/day) may have an up-regulatory effect on GLP-1 secretion in healthy subjects [418], thus explaining the improvement in postprandial insulin secretion. One study conducted on almond nuts reported improvements in glycemic control and lipid profiles in T2DM, without differences in IR vs controls [422]. A recent systematic review and meta-analysis of 9 RCTs examining the effects of almonds on gut microbiota, glucose metabolism and inflammatory parameters in T2DM reported that almond-based diets have significant effects in promoting the growth of short-chain fatty acid-producing gut microbiota, and lower HbA1c and body weight, but with no observed differences on the levels of fasting or 2-h postprandial blood glucose, inflammatory markers (C-reactive protein and tumour necrosis factor- α), GLP-1, fasting blood insulin, and IR [423]. Another systematic review and meta-analysis of 40 RCTs examining the effects of tree nuts on indices of glycemic control, reported that consumption of peanuts or tree nuts significantly decreased IR and fasting insulin, without effects on HbA1c or fasting glucose levels [424]. Walnuts have been shown to improve IR in another study [425]. A recent systematic review and meta-analysis of 16 RCTs examining the effects of walnuts on markers of blood glucose control, reported that consumption of walnuts did not result in significant changes in fasting blood glucose levels or IR, with available studies having either “some concern” or be “at high risk” of bias [426]. Although the study results regarding peanuts and tree-nuts on indices of glycemic control seem promising, high quality RCTs with larger population sizes and longer duration are needed to determine the exact efficacy, mechanism of action, precise daily dose, duration, and possible adverse effects of an effective intervention with these types of nuts in prediabetes and T2DM.

A recent systematic review and meta-analysis of 22 RCTs examining the effects of whey protein on glycemic status in patients with metabolic syndrome, reported that consumption of whey protein decreased significantly HbA1c, insulin/triglyceride/total cholesterol levels, and IR without effects on HDL cholesterol and fasting blood glucose levels [427]. Whey protein intake has been suggested to improve metabolic parameters due to bioactive substances, including immunoglobulins, glutamine, lactoferrin, and lactalbumin, which have been shown to activate the release of incretin hormones including GIP and GLP-1, whilst peptides from whey hydrolyzation have also been shown to inhibit dipeptidyl peptidase-IV and inhibit degradation of GIP and GLP-1 [427,428], all of which may have an important role in the improvement of IR [427]. Whey protein is an excellent source of branched-chain amino acids (BCAAs) and it has been shown that after its digestion, a rapid increase in amino acids, particularly BCAAs leads to insulin release, which may improve postprandial hyperglycemia [427].

In conclusion, until further well-designed of long duration studies are performed, it is safe and inexpensive to suggest that consumption of herbs, spices, such as cinnamon, fermented foods, such as vinegar, whey protein, peanuts, and tree nuts may offer some beneficial effects on postprandial glycemia and IR, additional to the overall diet implemented. Figure 2 and Figure 3 describe the key available study findings.

## 6. Conclusions 

Postprandial hyperglycemia and IR are complex issues influenced by many factors and causes. Their management is critical for the prevention of T2DM and amelioration of cardiometabolic risk factors. Diet is the cornerstone of glucose metabolism and weight loss is the remedy for IR. The extent and pace of weight loss, the dietary pattern of choice, and the macronutrient composition of the proposed diet remain to be elucidated with large, well-designed, long-term RCTs. Individualization and patient-centered approach should be the primary method of conduct. However, a balanced diet such as a Mediterranean-style, low GL diet may be a suitable approach to achieve both weight loss and maintenance and ameliorate postprandial hyperglycemia and IR. Lifestyle counseling using moderate energy restriction, regular physical activity and dietary behavior modification techniques has proven to be effective in optimally managing glycemic and insulinemic responses. However, dietary plans, foods and ingredients seem to have many differences when other variables are considered, such as glycemic or insulinemic responses or HbA1c. Although much more research is needed, some key points from the available scientific data for amelioration of postprandial hyperglycemia and IR may include the following: (a) lowering the total amount of carbohydrates consumed during the day to 40–50% of daily energy intake, such as in the case of Mediterranean-style diets, (b) consuming the majority of carbohydrates at lunch time, (c) adding lean proteins, plant proteins, and “good” fats (such as olive oil, peanuts and tree nuts, etc.) in meals, (d) following a meal sequence consuming vegetables first, then proteins and fats and then carbohydrates, particularly unprocessed ones, (e) choosing foods that do not lead to augmented glucose excursions and peaks to nadirs, (f) avoiding eating occasions late at night, and (h) consuming meals at consistent/regular times.

## Data Availability

Not applicable.

## Abbreviations

IR	Insulin resistance
T2DM	Type 2 diabetes
HDL	High density lipoprotein cholesterol
IGT	Impaired glucose tolerance
GI	Glycemic index
GL	Glycemic load
NEFA	Non-esterified free fatty acids
CVD	Cardiovascular disease
NAFLD	Non-alcoholic fatty liver disease
CNS	Central nervous system
HGP	Hepatic glucose production
GLP-1	Glucagon-like peptide 1
GIP	Glucose-dependent insulinotropic polypeptide
HbA1c	Glycated hemoglobin A1c
NGT	Normal glucose tolerance
BP	Blood pressure
RCT	Randomized Controlled Clinical Trial
ADA	American Diabetes Association
VLC	Very low carbohydrate
IRS	Insulin receptor substrate
GLUT	Glucose transporter
HOMA-IR	Homeostatic model assessment for insulin resistance
SCN	Suprachiasmatic nucleus
VLCD	Very low calorie diet
BMI	Body mass index
Per	Period
LDL	Low density lipoprotein cholesterol
PCO	Polycystic Ovary Syndrome
OGTT	Oral glucose tolerance test
IF	Intermittent fasting
ADF	Alternate day fasting
TRF	Time restricted feeding
IGF-1	Insulin-like growth factor-1
ROS	Reactive Oxygen Species

## References

[1] Shapira N. (2019). The Metabolic Concept of Meal Sequence vs. Satiety: Glycemic and Oxidative Responses with Reference to Inflammation Risk, Protective Principles and Mediterranean Diet. Nutrients.

[2] Nolan C.J., Ruderman N.B., Kahn S.E., Pedersen O., Prentki M. (2015). Insulin resistance as a physiological defense against metabolic stress: Implications for the management of subsets of type 2 diabetes. Diabetes.

[3] Melmer A., Laimer M. (2016). Treatment Goals in Diabetes. Endocr. Dev..

[4] Klein B.E., Klein R., Moss S.E., Cruickshanks K.J. (1996). Parental history of diabetes in a population-based study. Diabetes Care.

[5] Sigal R.J., El-Hashimy M., Martin B.C., Soeldner J.S., Krolewski A.S., Warram J.H. (1997). Acute postchallenge hyperinsulinemia predicts weight gain: A prospective study. Diabetes.

[6] Nathan D.M., Davidson M.B., DeFronzo R.A., Heine R.J., Henry R.R., Pratley R., Zinman B., American Diabetes Association (2007). Impaired fasting glucose and impaired glucose tolerance: Implications for care. Diabetes Care.

[7] Schwartz M.W., Seeley R.J., Zeltser L.M., Drewnowski A., Ravussin E., Redman L.M., Leibel R.L. (2017). Obesity Pathogenesis: An Endocrine Society Scientific Statement. Endocr. Rev..

[8] Lemieux S. (2001). Contribution of visceral obesity to the insulin resistance syndrome. Can. J. Appl. Physiol..

[9] Page M.M., Johnson J.D. (2018). Mild Suppression of Hyperinsulinemia to Treat Obesity and Insulin Resistance. Trends Endocrinol. Metab..

[10] Despres J.P. (2001). Health consequences of visceral obesity. Ann. Med..

[11] Bloomgarden Z.T. (2007). Insulin resistance, dyslipidemia, and cardiovascular disease. Diabetes Care.

[12] Philippou A., Chryssanthopoulos C., Maridaki M., Dimitriadis G., Koutsilieris M., Kokkinos P., Narayan P. (2019). Exercise metabolism in health and disease. Cardiorespiratory Fitness in Cardiometabolic Diseases.

[13] Lordan R., Tsoupras A., Mitra B., Zabetakis I. (2018). Dairy Fats and Cardiovascular Disease: Do We Really Need to be Concerned?. Foods.

[14] Unger A.L., Torres-Gonzalez M., Kraft J. (2019). Dairy Fat Consumption and the Risk of Metabolic Syndrome: An Examination of the Saturated Fatty Acids in Dairy. Nutrients.

[15] Dehghan M., Mente A., Rangarajan S., Sheridan P., Mohan V., Iqbal R., Gupta R., Lear S., Wentzel-Viljoen E., Avezum A. (2018). Association of dairy intake with cardiovascular disease and mortality in 21 countries from five continents (PURE): A prospective cohort study. Lancet.

[16] Rubin R. (2018). Whole-Fat or Nonfat Dairy? The Debate Continues. JAMA.

[17] McKeown N.M., Meigs J.B., Liu S., Saltzman E., Wilson P.W., Jacques P.F. (2004). Carbohydrate nutrition, insulin resistance, and the prevalence of the metabolic syndrome in the Framingham Offspring Cohort. Diabetes Care.

[18] Skyler J.S., Bakris G.L., Bonifacio E., Darsow T., Eckel R.H., Groop L., Groop P.H., Handelsman Y., Insel R.A., Mathieu C. (2017). Differentiation of Diabetes by Pathophysiology, Natural History, and Prognosis. Diabetes.

[19] Defronzo R.A. (2009). Banting Lecture. From the triumvirate to the ominous octet: A new paradigm for the treatment of type 2 diabetes mellitus. Diabetes.

[20] Trico D., Natali A., Arslanian S., Mari A., Ferrannini E. (2018). Identification, pathophysiology, and clinical implications of primary insulin hypersecretion in nondiabetic adults and adolescents. JCI Insight.

[21] Newsholme P., Cruzat V., Arfuso F., Keane K. (2014). Nutrient regulation of insulin secretion and action. J. Endocrinol..

[22] Natali A., Baldi S., Bonnet F., Petrie J., Trifiro S., Trico D., Mari A., Investigators R. (2017). Plasma HDL-cholesterol and triglycerides, but not LDL-cholesterol, are associated with insulin secretion in non-diabetic subjects. Metabolism.

[23] Dankner R., Chetrit A., Shanik M.H., Raz I., Roth J. (2009). Basal-state hyperinsulinemia in healthy normoglycemic adults is predictive of type 2 diabetes over a 24-year follow-up: A preliminary report. Diabetes Care.

[24] Sun Y., You W., Almeida F., Estabrooks P., Davy B. (2017). The Effectiveness and Cost of Lifestyle Interventions Including Nutrition Education for Diabetes Prevention: A Systematic Review and Meta-Analysis. J. Acad. Nutr. Diet.

[25] Cho N.H., Shaw J.E., Karuranga S., Huang Y., da Rocha Fernandes J.D., Ohlrogge A.W., Malanda B. (2018). IDF Diabetes Atlas: Global estimates of diabetes prevalence for 2017 and projections for 2045. Diabetes Res. Clin. Pr..

[26] Gregg E.W., Zhuo X., Cheng Y.J., Albright A.L., Narayan K.M., Thompson T.J. (2014). Trends in lifetime risk and years of life lost due to diabetes in the USA, 1985–2011: A modelling study. Lancet Diabetes Endocrinol..

[27] Sheng Z., Cao J.Y., Pang Y.C., Xu H.C., Chen J.W., Yuan J.H., Wang R., Zhang C.S., Wang L.X., Dong J. (2019). Effects of Lifestyle Modification and Anti-diabetic Medicine on Prediabetes Progress: A Systematic Review and Meta-Analysis. Front Endocrinol..

[28] Uusitupa M., Khan T.A., Viguiliouk E., Kahleova H., Rivellese A.A., Hermansen K., Pfeiffer A., Thanopoulou A., Salas-Salvado J., Schwab U. (2019). Prevention of Type 2 Diabetes by Lifestyle Changes: A Systematic Review and Meta-Analysis. Nutrients.

[29] Garcia-Molina L., Lewis-Mikhael A.M., Riquelme-Gallego B., Cano-Ibanez N., Oliveras-Lopez M.J., Bueno-Cavanillas A. (2020). Improving type 2 diabetes mellitus glycaemic control through lifestyle modification implementing diet intervention: A systematic review and meta-analysis. Eur. J. Nutr..

[30] Murphy B., Benson T., McCloat A., Mooney E., Elliott C., Dean M., Lavelle F. (2020). Changes in Consumers’ Food Practices during the COVID-19 Lockdown, Implications for Diet Quality and the Food System: A Cross-Continental Comparison. Nutrients.

[31] Dimitriadis G.D., Maratou E., Kountouri A., Board M., Lambadiari V. (2021). Regulation of Postabsorptive and Postprandial Glucose Metabolism by Insulin-Dependent and Insulin-Independent Mechanisms: An Integrative Approach. Nutrients.

[32] Dimitriadis G., Mitrou P., Lambadiari V., Maratou E., Raptis S.A. (2011). Insulin effects in muscle and adipose tissue. Diabetes Res. Clin. Pr..

[33] Gerich J.E. (2000). Physiology of glucose homeostasis. Diabetes Obes. Metab..

[34] Woerle H.J., Meyer C., Dostou J.M., Gosmanov N.R., Islam N., Popa E., Wittlin S.D., Welle S.L., Gerich J.E. (2003). Pathways for glucose disposal after meal ingestion in humans. Am. J. Physiol. Endocrinol. Metab..

[35] Tesfamariam B., Cohen R.A. (1992). Free radicals mediate endothelial cell dysfunction caused by elevated glucose. Am. J. Physiol..

[36] Nishikawa T., Edelstein D., Du X.L., Yamagishi S., Matsumura T., Kaneda Y., Yorek M.A., Beebe D., Oates P.J., Hammes H.P. (2000). Normalizing mitochondrial superoxide production blocks three pathways of hyperglycaemic damage. Nature.

[37] Ceriello A., Motz E. (2004). Is oxidative stress the pathogenic mechanism underlying insulin resistance, diabetes, and cardiovascular disease? The common soil hypothesis revisited. Arter. Thromb. Vasc. Biol..

[38] Stegenga M.E., van der Crabben S.N., Levi M., de Vos A.F., Tanck M.W., Sauerwein H.P., van der Poll T. (2006). Hyperglycemia stimulates coagulation, whereas hyperinsulinemia impairs fibrinolysis in healthy humans. Diabetes.

[39] Lemkes B.A., Hermanides J., Devries J.H., Holleman F., Meijers J.C., Hoekstra J.B. (2010). Hyperglycemia: A prothrombotic factor?. J. Thromb. Haemost..

[40] King G.L., Goodman A.D., Buzney S., Moses A., Kahn C.R. (1985). Receptors and growth-promoting effects of insulin and insulinlike growth factors on cells from bovine retinal capillaries and aorta. J. Clin. Investig..

[41] Monnier L., Colette C. (2008). Glycemic variability: Should we and can we prevent it?. Diabetes Care.

[42] Stout R.W. (1968). Insulin-stimulated lipogenesis in arterial tissue in relation to diabetes and atheroma. Lancet.

[43] Gamble J.M., Chibrikov E., Twells L.K., Midodzi W.K., Young S.W., MacDonald D., Majumdar S.R. (2017). Association of insulin dosage with mortality or major adverse cardiovascular events: A retrospective cohort study. Lancet Diabetes Endocrinol..

[44] Ferrannini E., DeFronzo R., DeFronzo R., Ferrannini E., Zimmet P., Alberti G. (2015). Insulin actions in vivo. International Textbook of Diabetes Mellitus.

[45] Jenkins D.J., Wolever T.M., Collier G.R., Ocana A., Rao A.V., Buckley G., Lam Y., Mayer A., Thompson L.U. (1987). Metabolic effects of a low-glycemic-index diet. Am. J. Clin. Nutr..

[46] Brynes A.E., Adamson J., Dornhorst A., Frost G.S. (2005). The beneficial effect of a diet with low glycaemic index on 24 h glucose profiles in healthy young people as assessed by continuous glucose monitoring. Br. J. Nutr..

[47] Agius L. (2013). High-carbohydrate diets induce hepatic insulin resistance to protect the liver from substrate overload. Biochem. Pharm..

[48] Meyer C., Dostou J.M., Welle S.L., Gerich J.E. (2002). Role of human liver, kidney, and skeletal muscle in postprandial glucose homeostasis. Am. J. Physiol. Endocrinol. Metab..

[49] Taylor R., Price T.B., Katz L.D., Shulman R.G., Shulman G.I. (1993). Direct measurement of change in muscle glycogen concentration after a mixed meal in normal subjects. Am. J. Physiol..

[50] Ferrannini E., Wahren J., Felig P., DeFronzo R.A. (1980). The role of fractional glucose extraction in the regulation of splanchnic glucose metabolism in normal and diabetic man. Metabolism.

[51] Kowalski G.M., Moore S.M., Hamley S., Selathurai A., Bruce C.R. (2017). The Effect of Ingested Glucose Dose on the Suppression of Endogenous Glucose Production in Humans. Diabetes.

[52] Baron A.D., Steinberg H., Brechtel G., Johnson A. (1994). Skeletal muscle blood flow independently modulates insulin-mediated glucose uptake. Am. J. Physiol..

[53] Karpe F., Fielding B.A., Ilic V., Macdonald I.A., Summers L.K., Frayn K.N. (2002). Impaired postprandial adipose tissue blood flow response is related to aspects of insulin sensitivity. Diabetes.

[54] Lambadiari V., Mitrou P., Maratou E., Raptis A., Raptis S.A., Dimitriadis G. (2012). Increases in muscle blood flow after a mixed meal are impaired at all stages of type 2 diabetes. Clin. Endocrinol..

[55] Dimitriadis G., Lambadiari V., Mitrou P., Maratou E., Boutati E., Panagiotakos D.B., Economopoulos T., Raptis S.A. (2007). Impaired postprandial blood flow in adipose tissue may be an early marker of insulin resistance in type 2 diabetes. Diabetes Care.

[56] Mitrou P., Boutati E., Lambadiari V., Maratou E., Papakonstantinou A., Komesidou V., Sidossis L., Tountas N., Katsilambros N., Economopoulos T. (2009). Rates of glucose uptake in adipose tissue and muscle in vivo after a mixed meal in women with morbid obesity. J. Clin. Endocrinol. Metab..

[57] Dimitriadis G., Newsholme E.A., LeRoith D., Taylor S., Olefsky J. (2003). Integration of Biochemical and Physiologic Effects of Insulin on the Control of Blood Glucose Concentrations. Diabetes Mellitus a Fundamental and Clinical Text.

[58] Kahn S.E., Prigeon R.L., McCulloch D.K., Boyko E.J., Bergman R.N., Schwartz M.W., Neifing J.L., Ward W.K., Beard J.C., Palmer J.P. (1993). Quantification of the relationship between insulin sensitivity and beta-cell function in human subjects. Evidence for a hyperbolic function. Diabetes.

[59] Basu A., Rizza R.A. (2001). Glucose effectiveness: Measurement in diabetic and nondiabetic humans. Exp. Clin. Endocrinol. Diabetes.

[60] Petersen K.F., Laurent D., Rothman D.L., Cline G.W., Shulman G.I. (1998). Mechanism by which glucose and insulin inhibit net hepatic glycogenolysis in humans. J. Clin. Investig..

[61] Mandarino L.J., Consoli A., Jain A., Kelley D.E. (1993). Differential regulation of intracellular glucose metabolism by glucose and insulin in human muscle. Am. J. Physiol..

[62] Marathe C.S., Rayner C.K., Jones K.L., Horowitz M. (2013). Relationships between gastric emptying, postprandial glycemia, and incretin hormones. Diabetes Care.

[63] O’Donovan D.G., Doran S., Feinle-Bisset C., Jones K.L., Meyer J.H., Wishart J.M., Morris H.A., Horowitz M. (2004). Effect of variations in small intestinal glucose delivery on plasma glucose, insulin, and incretin hormones in healthy subjects and type 2 diabetes. J. Clin. Endocrinol. Metab..

[64] Jenkins D.J., Wolever T.M., Ocana A.M., Vuksan V., Cunnane S.C., Jenkins M., Wong G.S., Singer W., Bloom S.R., Blendis L.M. (1990). Metabolic effects of reducing rate of glucose ingestion by single bolus versus continuous sipping. Diabetes.

[65] Holst J.J. (2013). Incretin hormones and the satiation signal. Int. J. Obes..

[66] Nauck M.A., Meier J.J. (2018). Incretin hormones: Their role in health and disease. Diabetes Obes. Metab..

[67] Gutniak M., Orskov C., Holst J.J., Ahren B., Efendic S. (1992). Antidiabetogenic effect of glucagon-like peptide-1 (7-36)amide in normal subjects and patients with diabetes mellitus. N. Engl. J. Med..

[68] Rowlands J., Heng J., Newsholme P., Carlessi R. (2018). Pleiotropic Effects of GLP-1 and Analogs on Cell Signaling, Metabolism, and Function. Front Endocrinol..

[69] Magnusson I., Chandramouli V., Schumann W.C., Kumaran K., Wahren J., Landau B.R. (1989). Pathways of hepatic glycogen formation in humans following ingestion of a glucose load in the fed state. Metabolism.

[70] Shulman G.I., Cline G., Schumann W.C., Chandramouli V., Kumaran K., Landau B.R. (1990). Quantitative comparison of pathways of hepatic glycogen repletion in fed and fasted humans. Am. J. Physiol..

[71] Bonuccelli S., Muscelli E., Gastaldelli A., Barsotti E., Astiarraga B.D., Holst J.J., Mari A., Ferrannini E. (2009). Improved tolerance to sequential glucose loading (Staub-Traugott effect): Size and mechanisms. Am. J. Physiol. Endocrinol. Metab..

[72] Jakubowicz D., Wainstein J., Ahren B., Landau Z., Bar-Dayan Y., Froy O. (2015). Fasting until noon triggers increased postprandial hyperglycemia and impaired insulin response after lunch and dinner in individuals with type 2 diabetes: A randomized clinical trial. Diabetes Care.

[73] Moore M.C., Smith M.S., Farmer B., Coate K.C., Kraft G., Shiota M., Williams P.E., Cherrington A.D. (2018). Morning Hyperinsulinemia Primes the Liver for Glucose Uptake and Glycogen Storage Later in the Day. Diabetes.

[74] Nesti L., Mengozzi A., Trico D. (2019). Impact of Nutrient Type and Sequence on Glucose Tolerance: Physiological Insights and Therapeutic Implications. Front Endocrinol..

[75] Vlachos D., Malisova S., Lindberg F.A., Karaniki G. (2020). Glycemic Index (GI) or Glycemic Load (GL) and Dietary Interventions for Optimizing Postprandial Hyperglycemia in Patients with T2 Diabetes: A Review. Nutrients.

[76] Korakas E., Dimitriadis G., Raptis A., Lambadiari V. (2018). Dietary Composition and Cardiovascular Risk: A Mediator or a Bystander?. Nutrients.

[77] Hyde P.N., Sapper T.N., Crabtree C.D., LaFountain R.A., Bowling M.L., Buga A., Fell B., McSwiney F.T., Dickerson R.M., Miller V.J. (2019). Dietary carbohydrate restriction improves metabolic syndrome independent of weight loss. JCI Insight.

[78] Meng H., Matthan N.R., Ausman L.M., Lichtenstein A.H. (2017). Effect of macronutrients and fiber on postprandial glycemic responses and meal glycemic index and glycemic load value determinations. Am. J. Clin. Nutr..

[79] Trico D., Natali A. (2017). Modulation of postprandial glycemic responses by noncarbohydrate nutrients provides novel approaches to the prevention and treatment of type 2 diabetes. Am. J. Clin. Nutr..

[80] Ceriello A., Esposito K., Piconi L., Ihnat M.A., Thorpe J.E., Testa R., Boemi M., Giugliano D. (2008). Oscillating glucose is more deleterious to endothelial function and oxidative stress than mean glucose in normal and type 2 diabetic patients. Diabetes.

[81] Monnier L., Colette C., Owens D.R. (2009). Integrating glycaemic variability in the glycaemic disorders of type 2 diabetes: A move towards a unified glucose tetrad concept. Diabetes Metab. Res. Rev..

[82] Saito Y., Kajiyama S., Nitta A., Miyawaki T., Matsumoto S., Ozasa N., Kajiyama S., Hashimoto Y., Fukui M., Imai S. (2020). Eating Fast Has a Significant Impact on Glycemic Excursion in Healthy Women: Randomized Controlled Cross-Over Trial. Nutrients.

[83] Pearce K.L., Noakes M., Keogh J., Clifton P.M. (2008). Effect of carbohydrate distribution on postprandial glucose peaks with the use of continuous glucose monitoring in type 2 diabetes. Am. J. Clin. Nutr..

[84] Kang X., Wang C., Lifang L., Chen D., Yang Y., Liu G., Wen H., Chen L., He L., Li X. (2013). Effects of different proportion of carbohydrate in breakfast on postprandial glucose excursion in normal glucose tolerance and impaired glucose regulation subjects. Diabetes Technol..

[85] Hafiz M.S., Campbell M.D., O’Mahoney L.L., Holmes M., Orfila C., Boesch C. (2021). Pulse consumption improves indices of glycemic control in adults with and without type 2 diabetes: A systematic review and meta-analysis of acute and long-term randomized controlled trials. Eur. J. Nutr..

[86] Ferreira H., Vasconcelos M., Gil A.M., Pinto E. (2021). Benefits of pulse consumption on metabolism and health: A systematic review of randomized controlled trials. Crit. Rev. Food Sci. Nutr..

[87] Bielefeld D., Grafenauer S., Rangan A. (2020). The Effects of Legume Consumption on Markers of Glycaemic Control in Individuals with and without Diabetes Mellitus: A Systematic Literature Review of Randomised Controlled Trials. Nutrients.

[88] Musa-Veloso K., Poon T., Harkness L.S., O’Shea M., Chu Y. (2018). The effects of whole-grain compared with refined wheat, rice, and rye on the postprandial blood glucose response: A systematic review and meta-analysis of randomized controlled trials. Am. J. Clin. Nutr..

[89] Reynolds A.N., Akerman A.P., Mann J. (2020). Dietary fibre and whole grains in diabetes management: Systematic review and meta-analyses. PLoS Med..

[90] Xu D., Fu L., Pan D., Lu Y., Yang C., Wang Y., Wang S., Sun G. (2021). Role of Whole Grain Consumption in Glycaemic Control of Diabetic Patients: A Systematic Review and Meta-Analysis of Randomized Controlled Trials. Nutrients.

[91] Sanders L.M., Zhu Y., Wilcox M.L., Koecher K., Maki K.C. (2021). Whole grain intake, compared to refined grain, improves postprandial glycemia and insulinemia: A systematic review and meta-analysis of randomized controlled trials. Crit. Rev. Food Sci. Nutr..

[92] Huang M., Li J., Ha M.A., Riccardi G., Liu S. (2017). A systematic review on the relations between pasta consumption and cardio-metabolic risk factors. Nutr. Metab. Cardiovasc. Dis..

[93] American Diabetes Association (2017). 4. Lifestyle Management. Diabetes Care.

[94] Esposito K., Chiodini P., Maiorino M.I., Bellastella G., Panagiotakos D., Giugliano D. (2014). Which diet for prevention of type 2 diabetes? A meta-analysis of prospective studies. Endocrine.

[95] Mirabelli M., Brunetti A. (2022). The Rise and Fall of the Mediterranean Diet and Related Nutrients in Preventing Diabetes. Nutrients.

[96] Mirabelli M., Mirabelli M., Chiefari E., Arcidiacono B., Corigliano D.M., Brunetti F.S., Maggisano V., Russo D., Foti D.P., Brunetti A. (2022). Mediterranean Diet Nutrients to Turn the Tide against Insulin Resistance and Related Diseases. Nutrients.

[97] Chiu T.H.T., Pan W.H., Lin M.N., Lin C.L. (2018). Vegetarian diet, change in dietary patterns, and diabetes risk: A prospective study. Nutr. Diabetes.

[98] Becerra-Tomas N., Diaz-Lopez A., Rosique-Esteban N., Ros E., Buil-Cosiales P., Corella D., Estruch R., Fito M., Serra-Majem L., Aros F. (2018). Legume consumption is inversely associated with type 2 diabetes incidence in adults: A prospective assessment from the PREDIMED study. Clin. Nutr..

[99] Lee Y., Park K. (2017). Adherence to a Vegetarian Diet and Diabetes Risk: A Systematic Review and Meta-Analysis of Observational Studies. Nutrients.

[100] Malik V.S., Li Y., Tobias D.K., Pan A., Hu F.B. (2016). Dietary Protein Intake and Risk of Type 2 Diabetes in US Men and Women. Am. J. Epidemiol..

[101] Lacoppidan S.A., Kyro C., Loft S., Helnaes A., Christensen J., Hansen C.P., Dahm C.C., Overvad K., Tjonneland A., Olsen A. (2015). Adherence to a Healthy Nordic Food Index Is Associated with a Lower Risk of Type-2 Diabetes--The Danish Diet, Cancer and Health Cohort Study. Nutrients.

[102] Schwingshackl L., Bogensberger B., Hoffmann G. (2018). Diet Quality as Assessed by the Healthy Eating Index, Alternate Healthy Eating Index, Dietary Approaches to Stop Hypertension Score, and Health Outcomes: An Updated Systematic Review and Meta-Analysis of Cohort Studies. J. Acad. Nutr. Diet..

[103] Salas-Salvado J., Bullo M., Estruch R., Ros E., Covas M.I., Ibarrola-Jurado N., Corella D., Aros F., Gomez-Gracia E., Ruiz-Gutierrez V. (2014). Prevention of diabetes with Mediterranean diets: A subgroup analysis of a randomized trial. Ann. Intern. Med..

[104] Salas-Salvado J., Bullo M., Babio N., Martinez-Gonzalez M.A., Ibarrola-Jurado N., Basora J., Estruch R., Covas M.I., Corella D., Aros F. (2011). Reduction in the incidence of type 2 diabetes with the Mediterranean diet: Results of the PREDIMED-Reus nutrition intervention randomized trial. Diabetes Care.

[105] Shai I., Schwarzfuchs D., Henkin Y., Shahar D.R., Witkow S., Greenberg I., Golan R., Fraser D., Bolotin A., Vardi H. (2008). Weight loss with a low-carbohydrate, Mediterranean, or low-fat diet. N. Engl. J. Med..

[106] Esposito K., Maiorino M.I., Petrizzo M., Bellastella G., Giugliano D. (2014). The effects of a Mediterranean diet on the need for diabetes drugs and remission of newly diagnosed type 2 diabetes: Follow-up of a randomized trial. Diabetes Care.

[107] Schwingshackl L., Chaimani A., Hoffmann G., Schwedhelm C., Boeing H. (2018). A network meta-analysis on the comparative efficacy of different dietary approaches on glycaemic control in patients with type 2 diabetes mellitus. Eur. J. Epidemiol..

[108] Ferrannini E., Mingrone G. (2009). Impact of different bariatric surgical procedures on insulin action and beta-cell function in type 2 diabetes. Diabetes Care.

[109] Kushner R.F. (2014). Weight loss strategies for treatment of obesity. Prog. Cardiovasc. Dis..

[110] Goodpaster B.H., Theriault R., Watkins S.C., Kelley D.E. (2000). Intramuscular lipid content is increased in obesity and decreased by weight loss. Metabolism.

[111] Corpeleijn E., Saris W.H., Blaak E.E. (2009). Metabolic flexibility in the development of insulin resistance and type 2 diabetes: Effects of lifestyle. Obes. Rev..

[112] Goodpaster B.H., Katsiaras A., Kelley D.E. (2003). Enhanced fat oxidation through physical activity is associated with improvements in insulin sensitivity in obesity. Diabetes.

[113] Corpeleijn E., Mensink M., Kooi M.E., Roekaerts P.M., Saris W.H., Blaak E.E. (2008). Impaired skeletal muscle substrate oxidation in glucose-intolerant men improves after weight loss. Obesity.

[114] Evert A.B., Dennison M., Gardner C.D., Garvey W.T., Lau K.H.K., MacLeod J., Mitri J., Pereira R.F., Rawlings K., Robinson S. (2019). Nutrition Therapy for Adults With Diabetes or Prediabetes: A Consensus Report. Diabetes Care.

[115] Knowler W.C., Barrett-Connor E., Fowler S.E., Hamman R.F., Lachin J.M., Walker E.A., Nathan D.M., Diabetes Prevention Program Research G. (2002). Reduction in the incidence of type 2 diabetes with lifestyle intervention or metformin. N. Engl. J. Med..

[116] Tuomilehto J., Lindstrom J., Eriksson J.G., Valle T.T., Hamalainen H., Ilanne-Parikka P., Keinanen-Kiukaanniemi S., Laakso M., Louheranta A., Rastas M. (2001). Prevention of type 2 diabetes mellitus by changes in lifestyle among subjects with impaired glucose tolerance. N. Engl. J. Med..

[117] Pan X.R., Li G.W., Hu Y.H., Wang J.X., Yang W.Y., An Z.X., Hu Z.X., Lin J., Xiao J.Z., Cao H.B. (1997). Effects of diet and exercise in preventing NIDDM in people with impaired glucose tolerance. The Da Qing IGT and Diabetes Study. Diabetes Care.

[118] Boule N.G., Haddad E., Kenny G.P., Wells G.A., Sigal R.J. (2001). Effects of exercise on glycemic control and body mass in type 2 diabetes mellitus: A meta-analysis of controlled clinical trials. JAMA.

[119] Franz M.J., Boucher J.L., Rutten-Ramos S., VanWormer J.J. (2015). Lifestyle weight-loss intervention outcomes in overweight and obese adults with type 2 diabetes: A systematic review and meta-analysis of randomized clinical trials. J. Acad. Nutr. Diet..

[120] Wadden T.A., Neiberg R.H., Wing R.R., Clark J.M., Delahanty L.M., Hill J.O., Krakoff J., Otto A., Ryan D.H., Vitolins M.Z. (2011). Four-year weight losses in the Look AHEAD study: Factors associated with long-term success. Obesity.

[121] Lean M.E., Leslie W.S., Barnes A.C., Brosnahan N., Thom G., McCombie L., Peters C., Zhyzhneuskaya S., Al-Mrabeh A., Hollingsworth K.G. (2018). Primary care-led weight management for remission of type 2 diabetes (DiRECT): An open-label, cluster-randomised trial. Lancet.

[122] Hamdy O., Mottalib A., Morsi A., El-Sayed N., Goebel-Fabbri A., Arathuzik G., Shahar J., Kirpitch A., Zrebiec J. (2017). Long-term effect of intensive lifestyle intervention on cardiovascular risk factors in patients with diabetes in real-world clinical practice: A 5-year longitudinal study. BMJ Open Diabetes Res. Care.

[123] Wing R.R., Look AHEAD Research Group (2010). Long-term effects of a lifestyle intervention on weight and cardiovascular risk factors in individuals with type 2 diabetes mellitus: Four-year results of the Look AHEAD trial. Arch. Intern. Med..

[124] Norris S.L., Zhang X., Avenell A., Gregg E., Brown T.J., Schmid C.H., Lau J. (2005). Long-term non-pharmacologic weight loss interventions for adults with type 2 diabetes. Cochrane Database Syst. Rev..

[125] Gregg E.W., Chen H., Wagenknecht L.E., Clark J.M., Delahanty L.M., Bantle J., Pownall H.J., Johnson K.C., Safford M.M., Kitabchi A.E. (2012). Association of an intensive lifestyle intervention with remission of type 2 diabetes. JAMA.

[126] Buse J.B., Caprio S., Cefalu W.T., Ceriello A., Del Prato S., Inzucchi S.E., McLaughlin S., Phillips G.L., Robertson R.P., Rubino F. (2009). How do we define cure of diabetes?. Diabetes Care.

[127] Zaghloul H., Chagoury O., Elhadad S., Hayder Ahmed S., Suleiman N., Al Naama A., El Nahas K., Al Hamaq A., Charlson M., Wells M.T. (2020). Clinical and metabolic characteristics of the Diabetes Intervention Accentuating Diet and Enhancing Metabolism (DIADEM-I) randomised clinical trial cohort. BMJ Open.

[128] Taheri S., Zaghloul H., Chagoury O., Elhadad S., Ahmed S.H., El Khatib N., Amona R.A., El Nahas K., Suleiman N., Alnaama A. (2020). Effect of intensive lifestyle intervention on bodyweight and glycaemia in early type 2 diabetes (DIADEM-I): An open-label, parallel-group, randomised controlled trial. Lancet Diabetes Endocrinol..

[129] Hall K.D., Kahan S. (2018). Maintenance of Lost Weight and Long-Term Management of Obesity. Med. Clin. North Am..

[130] Van Baak M.A., Mariman E.C.M. (2019). Mechanisms of weight regain after weight loss—The role of adipose tissue. Nat. Rev. Endocrinol..

[131] Astrup A., Grunwald G.K., Melanson E.L., Saris W.H., Hill J.O. (2000). The role of low-fat diets in body weight control: A meta-analysis of ad libitum dietary intervention studies. Int. J. Obes. Relat. Metab. Disord..

[132] Diabetes Prevention Program Research G., Knowler W.C., Fowler S.E., Hamman R.F., Christophi C.A., Hoffman H.J., Brenneman A.T., Brown-Friday J.O., Goldberg R., Venditti E. (2009). 10-year follow-up of diabetes incidence and weight loss in the Diabetes Prevention Program Outcomes Study. Lancet.

[133] Lindstrom J., Louheranta A., Mannelin M., Rastas M., Salminen V., Eriksson J., Uusitupa M., Tuomilehto J., Finnish Diabetes Prevention Study G. (2003). The Finnish Diabetes Prevention Study (DPS): Lifestyle intervention and 3-year results on diet and physical activity. Diabetes Care.

[134] Li G., Zhang P., Wang J., Gregg E.W., Yang W., Gong Q., Li H., Li H., Jiang Y., An Y. (2008). The long-term effect of lifestyle interventions to prevent diabetes in the China Da Qing Diabetes Prevention Study: A 20-year follow-up study. Lancet.

[135] Lindstrom J., Ilanne-Parikka P., Peltonen M., Aunola S., Eriksson J.G., Hemio K., Hamalainen H., Harkonen P., Keinanen-Kiukaanniemi S., Laakso M. (2006). Sustained reduction in the incidence of type 2 diabetes by lifestyle intervention: Follow-up of the Finnish Diabetes Prevention Study. Lancet.

[136] Diabetes Prevention Program Research G. (2015). Long-term effects of lifestyle intervention or metformin on diabetes development and microvascular complications over 15-year follow-up: The Diabetes Prevention Program Outcomes Study. Lancet Diabetes Endocrinol..

[137] Li G., Zhang P., Wang J., An Y., Gong Q., Gregg E.W., Yang W., Zhang B., Shuai Y., Hong J. (2014). Cardiovascular mortality, all-cause mortality, and diabetes incidence after lifestyle intervention for people with impaired glucose tolerance in the Da Qing Diabetes Prevention Study: A 23-year follow-up study. Lancet Diabetes Endocrinol..

[138] Hall K.D., Guo J. (2017). Obesity Energetics: Body Weight Regulation and the Effects of Diet Composition. Gastroenterology.

[139] Wheeler M.L., Dunbar S.A., Jaacks L.M., Karmally W., Mayer-Davis E.J., Wylie-Rosett J., Yancy W.S. (2012). Macronutrients, food groups, and eating patterns in the management of diabetes: A systematic review of the literature, 2010. Diabetes Care.

[140] Brehm B.J., Lattin B.L., Summer S.S., Boback J.A., Gilchrist G.M., Jandacek R.J., D’Alessio D.A. (2009). One-year comparison of a high-monounsaturated fat diet with a high-carbohydrate diet in type 2 diabetes. Diabetes Care.

[141] Davis N.J., Tomuta N., Schechter C., Isasi C.R., Segal-Isaacson C.J., Stein D., Zonszein J., Wylie-Rosett J. (2009). Comparative study of the effects of a 1-year dietary intervention of a low-carbohydrate diet versus a low-fat diet on weight and glycemic control in type 2 diabetes. Diabetes Care.

[142] Guldbrand H., Dizdar B., Bunjaku B., Lindstrom T., Bachrach-Lindstrom M., Fredrikson M., Ostgren C.J., Nystrom F.H. (2012). In type 2 diabetes, randomisation to advice to follow a low-carbohydrate diet transiently improves glycaemic control compared with advice to follow a low-fat diet producing a similar weight loss. Diabetologia.

[143] Papakonstantinou E., Triantafillidou D., Panagiotakos D.B., Koutsovasilis A., Saliaris M., Manolis A., Melidonis A., Zampelas A. (2010). A high-protein low-fat diet is more effective in improving blood pressure and triglycerides in calorie-restricted obese individuals with newly diagnosed type 2 diabetes. Eur. J. Clin. Nutr..

[144] Kodama S., Saito K., Tanaka S., Maki M., Yachi Y., Sato M., Sugawara A., Totsuka K., Shimano H., Ohashi Y. (2009). Influence of fat and carbohydrate proportions on the metabolic profile in patients with type 2 diabetes: A meta-analysis. Diabetes Care.

[145] Wing R.R., Bolin P., Brancati F.L., Bray G.A., Clark J.M., Coday M., Crow R.S., Curtis J.M., Egan C.M., Look AHEAD Research Group (2013). Cardiovascular effects of intensive lifestyle intervention in type 2 diabetes. N. Engl. J. Med..

[146] Pi-Sunyer X., Blackburn G., Brancati F.L., Bray G.A., Bright R., Clark J.M., Curtis J.M., Espeland M.A., Foreyt J.P., Look AHEAD Research Group (2007). Reduction in weight and cardiovascular disease risk factors in individuals with type 2 diabetes: One-year results of the look AHEAD trial. Diabetes Care.

[147] Grandl G., Straub L., Rudigier C., Arnold M., Wueest S., Konrad D., Wolfrum C. (2018). Short-term feeding of a ketogenic diet induces more severe hepatic insulin resistance than an obesogenic high-fat diet. J. Physiol..

[148] Johansen M.Y., MacDonald C.S., Hansen K.B., Karstoft K., Christensen R., Pedersen M., Hansen L.S., Zacho M., Wedell-Neergaard A.S., Nielsen S.T. (2017). Effect of an Intensive Lifestyle Intervention on Glycemic Control in Patients With Type 2 Diabetes: A Randomized Clinical Trial. Jama.

[149] Linmans J.J., Spigt M.G., Deneer L., Lucas A.E., de Bakker M., Gidding L.G., Linssen R., Knottnerus J.A. (2011). Effect of lifestyle intervention for people with diabetes or prediabetes in real-world primary care: Propensity score analysis. BMC Fam. Pract..

[150] Chee W.S.S., Gilcharan Singh H.K., Hamdy O., Mechanick J.I., Lee V.K.M., Barua A., Mohd Ali S.Z., Hussein Z. (2017). Structured lifestyle intervention based on a trans-cultural diabetes-specific nutrition algorithm (tDNA) in individuals with type 2 diabetes: A randomized controlled trial. BMJ Open Diabetes Res. Care.

[151] Ujvari D., Hulchiy M., Calaby A., Nybacka A., Bystrom B., Hirschberg A.L. (2014). Lifestyle intervention up-regulates gene and protein levels of molecules involved in insulin signaling in the endometrium of overweight/obese women with polycystic ovary syndrome. Hum. Reprod..

[152] O’Brien M.J., Perez A., Scanlan A.B., Alos V.A., Whitaker R.C., Foster G.D., Ackermann R.T., Ciolino J.D., Homko C. (2017). PREVENT-DM Comparative Effectiveness Trial of Lifestyle Intervention and Metformin. Am. J. Prev. Med..

[153] Slentz C.A., Bateman L.A., Willis L.H., Granville E.O., Piner L.W., Samsa G.P., Setji T.L., Muehlbauer M.J., Huffman K.M., Bales C.W. (2016). Effects of exercise training alone vs a combined exercise and nutritional lifestyle intervention on glucose homeostasis in prediabetic individuals: A randomised controlled trial. Diabetologia.

[154] Mensink M., Blaak E.E., Corpeleijn E., Saris W.H., de Bruin T.W., Feskens E.J. (2003). Lifestyle intervention according to general recommendations improves glucose tolerance. Obes. Res..

[155] Roumen C., Corpeleijn E., Feskens E.J., Mensink M., Saris W.H., Blaak E.E. (2008). Impact of 3-year lifestyle intervention on postprandial glucose metabolism: The SLIM study. Diabetes Med..

[156] Unwin D., Khalid A.A., Unwin J., Crocombe D., Delon C., Martyn K., Golubic R., Ray S. (2020). Insights from a general practice service evaluation supporting a lower carbohydrate diet in patients with type 2 diabetes mellitus and prediabetes: A secondary analysis of routine clinic data including HbA1c, weight and prescribing over 6 years. BMJ. Nutr. Prev. Health.

[157] Chawla S., Tessarolo Silva F., Amaral Medeiros S., Mekary R.A., Radenkovic D. (2020). The Effect of Low-Fat and Low-Carbohydrate Diets on Weight Loss and Lipid Levels: A Systematic Review and Meta-Analysis. Nutrients.

[158] Van Zuuren E.J., Fedorowicz Z., Kuijpers T., Pijl H. (2018). Effects of low-carbohydrate- compared with low-fat-diet interventions on metabolic control in people with type 2 diabetes: A systematic review including GRADE assessments. Am. J. Clin. Nutr..

[159] Sainsbury E., Kizirian N.V., Partridge S.R., Gill T., Colagiuri S., Gibson A.A. (2018). Effect of dietary carbohydrate restriction on glycemic control in adults with diabetes: A systematic review and meta-analysis. Diabetes Res. Clin. Pr..

[160] Jonsson T., Granfeldt Y., Ahren B., Branell U.C., Palsson G., Hansson A., Soderstrom M., Lindeberg S. (2009). Beneficial effects of a Paleolithic diet on cardiovascular risk factors in type 2 diabetes: A randomized cross-over pilot study. Cardiovasc. Diabetol..

[161] Masharani U., Sherchan P., Schloetter M., Stratford S., Xiao A., Sebastian A., Nolte Kennedy M., Frassetto L. (2015). Metabolic and physiologic effects from consuming a hunter-gatherer (Paleolithic)-type diet in type 2 diabetes. Eur. J. Clin. Nutr..

[162] Lindeberg S., Jonsson T., Granfeldt Y., Borgstrand E., Soffman J., Sjostrom K., Ahren B. (2007). A Palaeolithic diet improves glucose tolerance more than a Mediterranean-like diet in individuals with ischaemic heart disease. Diabetologia.

[163] Goldenberg J.Z., Day A., Brinkworth G.D., Sato J., Yamada S., Jonsson T., Beardsley J., Johnson J.A., Thabane L., Johnston B.C. (2021). Efficacy and safety of low and very low carbohydrate diets for type 2 diabetes remission: Systematic review and meta-analysis of published and unpublished randomized trial data. BMJ.

[164] Snorgaard O., Poulsen G.M., Andersen H.K., Astrup A. (2017). Systematic review and meta-analysis of dietary carbohydrate restriction in patients with type 2 diabetes. BMJ Open Diabetes Res. Care.

[165] Dansinger M.L., Gleason J.A., Griffith J.L., Selker H.P., Schaefer E.J. (2005). Comparison of the Atkins, Ornish, Weight Watchers, and Zone diets for weight loss and heart disease risk reduction: A randomized trial. JAMA.

[166] McClain A.D., Otten J.J., Hekler E.B., Gardner C.D. (2013). Adherence to a low-fat vs. low-carbohydrate diet differs by insulin resistance status. Diabetes Obes. Metab..

[167] Thom G., Lean M. (2017). Is There an Optimal Diet for Weight Management and Metabolic Health?. Gastroenterology.

[168] Johnston B.C., Kanters S., Bandayrel K., Wu P., Naji F., Siemieniuk R.A., Ball G.D., Busse J.W., Thorlund K., Guyatt G. (2014). Comparison of weight loss among named diet programs in overweight and obese adults: A meta-analysis. JAMA.

[169] Clifton P.M., Keogh J.B. (2018). Effects of Different Weight Loss Approaches on CVD Risk. Curr. Atheroscler. Rep..

[170] Noakes T.D., Windt J. (2017). Evidence that supports the prescription of low-carbohydrate high-fat diets: A narrative review. Br. J. Sports Med..

[171] Feinman R.D., Pogozelski W.K., Astrup A., Bernstein R.K., Fine E.J., Westman E.C., Accurso A., Frassetto L., Gower B.A., McFarlane S.I. (2015). Dietary carbohydrate restriction as the first approach in diabetes management: Critical review and evidence base. Nutrition.

[172] Cao Y., Mauger D.T., Pelkman C.L., Zhao G., Townsend S.M., Kris-Etherton P.M. (2009). Effects of moderate (MF) versus lower fat (LF) diets on lipids and lipoproteins: A meta-analysis of clinical trials in subjects with and without diabetes. J. Clin. Lipidol..

[173] Garg A. (1998). High-monounsaturated-fat diets for patients with diabetes mellitus: A meta-analysis. Am. J. Clin. Nutr..

[174] Horikawa C., Yoshimura Y., Kamada C., Tanaka S., Tanaka S., Matsunaga S., Hanyu O., Araki A., Ito H., Tanaka A. (2017). Is the Proportion of Carbohydrate Intake Associated with the Incidence of Diabetes Complications?-An Analysis of the Japan Diabetes Complications Study. Nutrients.

[175] Vitale M., Masulli M., Rivellese A.A., Babini A.C., Boemi M., Bonora E., Buzzetti R., Ciano O., Cignarelli M., Cigolini M. (2016). Influence of dietary fat and carbohydrates proportions on plasma lipids, glucose control and low-grade inflammation in patients with type 2 diabetes-The TOSCA.IT Study. Eur. J. Nutr..

[176] Look AHEAD Research Group (2014). Effect of a long-term behavioural weight loss intervention on nephropathy in overweight or obese adults with type 2 diabetes: A secondary analysis of the Look AHEAD randomised clinical trial. Lancet Diabetes Endocrinol..

[177] Churuangsuk C., Hall J., Reynolds A., Griffin S.J., Combet E., Lean M.E.J. (2022). Diets for weight management in adults with type 2 diabetes: An umbrella review of published meta-analyses and systematic review of trials of diets for diabetes remission. Diabetologia.

[178] Nield L., Moore H.J., Hooper L., Cruickshank J.K., Vyas A., Whittaker V., Summerbell C.D. (2007). Dietary advice for treatment of type 2 diabetes mellitus in adults. Cochrane Database Syst. Rev..

[179] Franz M.J. (2016). Diabetes Nutrition Therapy: Effectiveness, Macronutrients, Eating Patterns and Weight Management. Am. J. Med. Sci..

[180] Trico D., Baldi S., Tulipani A., Frascerra S., Macedo M.P., Mari A., Ferrannini E., Natali A. (2015). Mechanisms through which a small protein and lipid preload improves glucose tolerance. Diabetologia.

[181] Gentilcore D., Chaikomin R., Jones K.L., Russo A., Feinle-Bisset C., Wishart J.M., Rayner C.K., Horowitz M. (2006). Effects of fat on gastric emptying of and the glycemic, insulin, and incretin responses to a carbohydrate meal in type 2 diabetes. J. Clin. Endocrinol. Metab..

[182] Ma J., Stevens J.E., Cukier K., Maddox A.F., Wishart J.M., Jones K.L., Clifton P.M., Horowitz M., Rayner C.K. (2009). Effects of a protein preload on gastric emptying, glycemia, and gut hormones after a carbohydrate meal in diet-controlled type 2 diabetes. Diabetes Care.

[183] Shukla A.P., Iliescu R.G., Thomas C.E., Aronne L.J. (2015). Food Order Has a Significant Impact on Postprandial Glucose and Insulin Levels. Diabetes Care.

[184] Sun L., Goh H.J., Govindharajulu P., Leow M.K., Henry C.J. (2020). Postprandial glucose, insulin and incretin responses differ by test meal macronutrient ingestion sequence (PATTERN study). Clin. Nutr..

[185] Weickert M.O., Pfeiffer A.F. (2008). Metabolic effects of dietary fiber consumption and prevention of diabetes. J. Nutr..

[186] Krezowski P.A., Nuttall F.Q., Gannon M.C., Bartosh N.H. (1986). The effect of protein ingestion on the metabolic response to oral glucose in normal individuals. Am. J. Clin. Nutr..

[187] Nuttall F.Q., Mooradian A.D., Gannon M.C., Billington C., Krezowski P. (1984). Effect of protein ingestion on the glucose and insulin response to a standardized oral glucose load. Diabetes Care.

[188] Clifton P.M., Galbraith C., Coles L. (2014). Effect of a low dose whey/guar preload on glycemic control in people with type 2 diabetes--a randomised controlled trial. Nutr. J..

[189] Watson L.E., Phillips L.K., Wu T., Bound M.J., Checklin H.L., Grivell J., Jones K.L., Clifton P.M., Horowitz M., Rayner C.K. (2019). A whey/guar “preload” improves postprandial glycaemia and glycated haemoglobin levels in type 2 diabetes: A 12-week, single-blind, randomized, placebo-controlled trial. Diabetes Obes. Metab..

[190] Rigamonti A.E., Leoncini R., Casnici C., Marelli O., Col A., Tamini S., Lucchetti E., Cicolini S., Abbruzzese L., Cella S.G. (2019). Whey Proteins Reduce Appetite, Stimulate Anorexigenic Gastrointestinal Peptides and Improve Glucometabolic Homeostasis in Young Obese Women. Nutrients.

[191] Kubota S., Liu Y., Iizuka K., Kuwata H., Seino Y., Yabe D. (2020). A Review of Recent Findings on Meal Sequence: An Attractive Dietary Approach to Prevention and Management of Type 2 Diabetes. Nutrients.

[192] Wadden T.A., Tronieri J.S., Butryn M.L. (2020). Lifestyle modification approaches for the treatment of obesity in adults. Am. Psychol..

[193] American Diabetes Association (2019). 5. Lifestyle Management: Standards of Medical Care in Diabetes-2019. Diabetes Care.

[194] Saslow L.R., Mason A.E., Kim S., Goldman V., Ploutz-Snyder R., Bayandorian H., Daubenmier J., Hecht F.M., Moskowitz J.T. (2017). An Online Intervention Comparing a Very Low-Carbohydrate Ketogenic Diet and Lifestyle Recommendations Versus a Plate Method Diet in Overweight Individuals With Type 2 Diabetes: A Randomized Controlled Trial. J. Med. Internet Res..

[195] Pot G.K., Battjes-Fries M.C., Patijn O.N., Pijl H., Witkamp R.F., de Visser M., van der Zijl N., de Vries M., Voshol P.J. (2019). Nutrition and lifestyle intervention in type 2 diabetes: Pilot study in the Netherlands showing improved glucose control and reduction in glucose lowering medication. BMJ. Nutr. Prev. Health.

[196] Burke L.E., Styn M.A., Sereika S.M., Conroy M.B., Ye L., Glanz K., Sevick M.A., Ewing L.J. (2012). Using mHealth technology to enhance self-monitoring for weight loss: A randomized trial. Am. J. Prev. Med..

[197] Theodoropoulou K.T., Dimitriadis G.D., Tentolouris N., Darviri C., Chrousos G.P. (2020). Diabetes distress is associated with individualized glycemic control in adults with type 2 diabetes mellitus. Hormones.

[198] Gummesson A., Nyman E., Knutsson M., Karpefors M. (2017). Effect of weight reduction on glycated haemoglobin in weight loss trials in patients with type 2 diabetes. Diabetes Obes. Metab..

[199] Mensink M., Blaak E.E., Vidal H., De Bruin T.W., Glatz J.F., Saris W.H. (2003). Lifestyle changes and lipid metabolism gene expression and protein content in skeletal muscle of subjects with impaired glucose tolerance. Diabetologia.

[200] Savikj M., Gabriel B.M., Alm P.S., Smith J., Caidahl K., Bjornholm M., Fritz T., Krook A., Zierath J.R., Wallberg-Henriksson H. (2019). Afternoon exercise is more efficacious than morning exercise at improving blood glucose levels in individuals with type 2 diabetes: A randomised crossover trial. Diabetologia.

[201] Aqeel M., Forster A., Richards E.A., Hennessy E., McGowan B., Bhadra A., Guo J., Gelfand S., Delp E., Eicher-Miller H.A. (2020). The Effect of Timing of Exercise and Eating on Postprandial Response in Adults: A Systematic Review. Nutrients.

[202] Toledo F.G., Menshikova E.V., Azuma K., Radikova Z., Kelley C.A., Ritov V.B., Kelley D.E. (2008). Mitochondrial capacity in skeletal muscle is not stimulated by weight loss despite increases in insulin action and decreases in intramyocellular lipid content. Diabetes.

[203] Dunkley A.J., Charles K., Gray L.J., Camosso-Stefinovic J., Davies M.J., Khunti K. (2012). Effectiveness of interventions for reducing diabetes and cardiovascular disease risk in people with metabolic syndrome: Systematic review and mixed treatment comparison meta-analysis. Diabetes Obes. Metab..

[204] Schellenberg E.S., Dryden D.M., Vandermeer B., Ha C., Korownyk C. (2013). Lifestyle interventions for patients with and at risk for type 2 diabetes: A systematic review and meta-analysis. Ann. Intern. Med..

[205] Terranova C.O., Brakenridge C.L., Lawler S.P., Eakin E.G., Reeves M.M. (2015). Effectiveness of lifestyle-based weight loss interventions for adults with type 2 diabetes: A systematic review and meta-analysis. Diabetes Obes. Metab..

[206] Brinks J., Fowler A., Franklin B.A., Dulai J. (2017). Lifestyle Modification in Secondary Prevention: Beyond Pharmacotherapy. Am. J. Lifestyle Med..

[207] Schwingshackl L., Dias S., Hoffmann G. (2014). Impact of long-term lifestyle programmes on weight loss and cardiovascular risk factors in overweight/obese participants: A systematic review and network meta-analysis. Syst. Rev..

[208] Chen L., Pei J.H., Kuang J., Chen H.M., Chen Z., Li Z.W., Yang H.Z. (2015). Effect of lifestyle intervention in patients with type 2 diabetes: A meta-analysis. Metabolism.

[209] Tuso P. (2014). Prediabetes and lifestyle modification: Time to prevent a preventable disease. Perm. J..

[210] Looney S.M., Raynor H.A. (2013). Behavioral lifestyle intervention in the treatment of obesity. Health Serv. Insights.

[211] Solomon T.P., Haus J.M., Kelly K.R., Rocco M., Kashyap S.R., Kirwan J.P. (2010). Improved pancreatic beta-cell function in type 2 diabetic patients after lifestyle-induced weight loss is related to glucose-dependent insulinotropic polypeptide. Diabetes Care.

[212] Baak M.A., Pramono A., Battista F., Beaulieu K., Blundell J.E., Busetto L., Carraca E.V., Dicker D., Encantado J., Ermolao A. (2021). Effect of different types of regular exercise on physical fittness in adults with overweight or obesity: Systematic review and meta-analyses. Obes. Rev..

[213] Batacan R.B., Duncan M.J., Dalbo V.J., Tucker P.S., Fenning A.S. (2017). Effects of high-intensity interval training on cardiometabolic health: A systematic review and meta-analysis of intervention studies. Br. J. Sports Med..

[214] Miles J.M., Wooldridge D., Grellner W.J., Windsor S., Isley W.L., Klein S., Harris W.S. (2003). Nocturnal and postprandial free fatty acid kinetics in normal and type 2 diabetic subjects: Effects of insulin sensitization therapy. Diabetes.

[215] James D.E., Kraegen E.W., Chisholm D.J. (1985). Effects of exercise training on in vivo insulin action in individual tissues of the rat. J. Clin. Investig..

[216] Romijn J.A., Coyle E.F., Sidossis L.S., Gastaldelli A., Horowitz J.F., Endert E., Wolfe R.R. (1993). Regulation of endogenous fat and carbohydrate metabolism in relation to exercise intensity and duration. Am. J. Physiol..

[217] Wahren J., Felig P., Ahlborg G., Jorfeldt L. (1971). Glucose metabolism during leg exercise in man. J. Clin. Investig..

[218] Clutter W.E., Bier D.M., Shah S.D., Cryer P.E. (1980). Epinephrine plasma metabolic clearance rates and physiologic thresholds for metabolic and hemodynamic actions in man. J. Clin. Investig..

[219] Moghetti P., Bacchi E., Brangani C., Dona S., Negri C. (2016). Metabolic Effects of Exercise. Front Horm. Res..

[220] Coderre L., Kandror K.V., Vallega G., Pilch P.F. (1995). Identification and characterization of an exercise-sensitive pool of glucose transporters in skeletal muscle. J. Biol. Chem..

[221] Leighton B., Blomstrand E., Challiss R.A., Lozeman F.J., Parry-Billings M., Dimitriadis G.D., Newsholme E.A. (1989). Acute and chronic effects of strenuous exercise on glucose metabolism in isolated, incubated soleus muscle of exercise-trained rats. Acta Physiol. Scand..

[222] Ploug T., Galbo H., Richter E.A. (1984). Increased muscle glucose uptake during contractions: No need for insulin. Am. J. Physiol..

[223] Jensen T.E., Richter E.A. (2012). Regulation of glucose and glycogen metabolism during and after exercise. J. Physiol..

[224] Gejl K.D., Ortenblad N., Andersson E., Plomgaard P., Holmberg H.C., Nielsen J. (2017). Local depletion of glycogen with supramaximal exercise in human skeletal muscle fibres. J. Physiol..

[225] Mulya A., Haus J.M., Solomon T.P., Kelly K.R., Malin S.K., Rocco M., Barkoukis H., Kirwan J.P. (2017). Exercise training-induced improvement in skeletal muscle PGC-1alpha-mediated fat metabolism is independent of dietary glycemic index. Obesity.

[226] Newsholme E.A. (1980). Sounding Board. A possible metabolic basis for the control of body weight. N. Engl. J. Med..

[227] Codella R., Ialacqua M., Terruzzi I., Luzi L. (2018). May the force be with you: Why resistance training is essential for subjects with type 2 diabetes mellitus without complications. Endocrine.

[228] Kido K., Ato S., Yokokawa T., Makanae Y., Sato K., Fujita S. (2016). Acute resistance exercise-induced IGF1 expression and subsequent GLUT4 translocation. Physiol. Rep..

[229] Mangine G.T., Hoffman J.R., Gonzalez A.M., Townsend J.R., Wells A.J., Jajtner A.R., Beyer K.S., Boone C.H., Miramonti A.A., Wang R. (2015). The effect of training volume and intensity on improvements in muscular strength and size in resistance-trained men. Physiol. Rep..

[230] Dimitriadis G., Parry-Billings M., Bevan S., Dunger D., Piva T., Krause U., Wegener G., Newsholme E.A. (1992). Effects of insulin-like growth factor I on the rates of glucose transport and utilization in rat skeletal muscle in vitro. Biochem. J..

[231] Dimitriadis G., Parry-Billings M., Dunger D., Bevan S., Colquhoun A., Taylor A., Calder P., Krause U., Wegener G., Newsholme E.A. (1992). Effects of in-vivo administration of insulin-like growth factor-I on the rate of glucose utilization in the soleus muscle of the rat. J. Endocrinol..

[232] Poehlman E.T., Dvorak R.V., DeNino W.F., Brochu M., Ades P.A. (2000). Effects of resistance training and endurance training on insulin sensitivity in nonobese, young women: A controlled randomized trial. J. Clin. Endocrinol. Metab..

[233] Paquin J., Lagace J.C., Brochu M., Dionne I.J. (2021). Exercising for Insulin Sensitivity—Is There a Mechanistic Relationship With Quantitative Changes in Skeletal Muscle Mass?. Front Physiol..

[234] Tjonna A.E., Lee S.J., Rognmo O., Stolen T.O., Bye A., Haram P.M., Loennechen J.P., Al-Share Q.Y., Skogvoll E., Slordahl S.A. (2008). Aerobic interval training versus continuous moderate exercise as a treatment for the metabolic syndrome: A pilot study. Circulation.

[235] Garber C.E., Blissmer B., Deschenes M.R., Franklin B.A., Lamonte M.J., Lee I.M., Nieman D.C., Swain D.P., American College of Sports M. (2011). American College of Sports Medicine position stand. Quantity and quality of exercise for developing and maintaining cardiorespiratory, musculoskeletal, and neuromotor fitness in apparently healthy adults: Guidance for prescribing exercise. Med. Sci. Sports Exerc..

[236] Kirwan J.P., Sacks J., Nieuwoudt S. (2017). The essential role of exercise in the management of type 2 diabetes. Cleve. Clin. J. Med..

[237] Riddell M.C., Gallen I.W., Smart C.E., Taplin C.E., Adolfsson P., Lumb A.N., Kowalski A., Rabasa-Lhoret R., McCrimmon R.J., Hume C. (2017). Exercise management in type 1 diabetes: A consensus statement. Lancet Diabetes Endocrinol..

[238] De Matos M.A., Vieira D.V., Pinhal K.C., Lopes J.F., Dias-Peixoto M.F., Pauli J.R., de Castro Magalhaes F., Little J.P., Rocha-Vieira E., Amorim F.T. (2018). High-Intensity Interval Training Improves Markers of Oxidative Metabolism in Skeletal Muscle of Individuals With Obesity and Insulin Resistance. Front Physiol..

[239] Acosta-Manzano P., Rodriguez-Ayllon M., Acosta F.M., Niederseer D., Niebauer J. (2020). Beyond general resistance training. Hypertrophy versus muscular endurance training as therapeutic interventions in adults with type 2 diabetes mellitus: A systematic review and meta-analysis. Obes. Rev..

[240] Iaccarino G., Franco D., Sorriento D., Strisciuglio T., Barbato E., Morisco C. (2021). Modulation of Insulin Sensitivity by Exercise Training: Implications for Cardiovascular Prevention. J. Cardiovasc. Transl. Res..

[241] Palermi S., Iacono O., Sirico F., Modestino M., Ruosi C., Spera R., De Luca M. (2021). The complex relationship between physical activity and diabetes: An overview. J. Basic Clin. Physiol. Pharm..

[242] American Diabetes Association Professional Practice Committee (2022). 17. Diabetes Advocacy: Standards of Medical Care in Diabetes-2022. Diabetes Care.

[243] Sulli G., Manoogian E.N.C., Taub P.R., Panda S. (2018). Training the Circadian Clock, Clocking the Drugs, and Drugging the Clock to Prevent, Manage, and Treat Chronic Diseases. Trends Pharm. Sci..

[244] Voigt R.M., Forsyth C.B., Keshavarzian A. (2019). Circadian rhythms: A regulator of gastrointestinal health and dysfunction. Expert Rev. Gastroenterol. Hepatol..

[245] Bozek K., Relogio A., Kielbasa S.M., Heine M., Dame C., Kramer A., Herzel H. (2009). Regulation of clock-controlled genes in mammals. PLoS ONE.

[246] Hirayama J., Sassone-Corsi P. (2005). Structural and functional features of transcription factors controlling the circadian clock. Curr. Opin. Genet. Dev..

[247] Menet J.S., Pescatore S., Rosbash M. (2014). CLOCK:BMAL1 is a pioneer-like transcription factor. Genes Dev..

[248] Kalsbeek A., la Fleur S., Fliers E. (2014). Circadian control of glucose metabolism. Mol. Metab..

[249] Manoogian E.N.C., Panda S. (2017). Circadian rhythms, time-restricted feeding, and healthy aging. Ageing Res. Rev..

[250] Goel N., Stunkard A.J., Rogers N.L., Van Dongen H.P., Allison K.C., O’Reardon J.P., Ahima R.S., Cummings D.E., Heo M., Dinges D.F. (2009). Circadian rhythm profiles in women with night eating syndrome. J. Biol. Rhythm..

[251] Sadacca L.A., Lamia K.A., deLemos A.S., Blum B., Weitz C.J. (2011). An intrinsic circadian clock of the pancreas is required for normal insulin release and glucose homeostasis in mice. Diabetologia.

[252] Kalsbeek A., Foppen E., Schalij I., Van Heijningen C., van der Vliet J., Fliers E., Buijs R.M. (2008). Circadian control of the daily plasma glucose rhythm: An interplay of GABA and glutamate. PLoS ONE.

[253] Vieira E., Merino B., Quesada I. (2015). Role of the clock gene Rev-erbalpha in metabolism and in the endocrine pancreas. Diabetes Obes. Metab..

[254] Muhlbauer E., Wolgast S., Finckh U., Peschke D., Peschke E. (2004). Indication of circadian oscillations in the rat pancreas. FEBS Lett.

[255] Dickmeis T. (2009). Glucocorticoids and the circadian clock. J. Endocrinol..

[256] Asher G., Sassone-Corsi P. (2015). Time for food: The intimate interplay between nutrition, metabolism, and the circadian clock. Cell.

[257] Gill S., Panda S. (2015). A Smartphone App Reveals Erratic Diurnal Eating Patterns in Humans that Can Be Modulated for Health Benefits. Cell Metab..

[258] Lund J., Arendt J., Hampton S.M., English J., Morgan L.M. (2001). Postprandial hormone and metabolic responses amongst shift workers in Antarctica. J. Endocrinol..

[259] Zimberg I.Z., Fernandes Junior S.A., Crispim C.A., Tufik S., de Mello M.T. (2012). Metabolic impact of shift work. Work.

[260] Henry C.J., Kaur B., Quek R.Y.C. (2020). Chrononutrition in the management of diabetes. Nutr. Diabetes.

[261] Hara R., Wan K., Wakamatsu H., Aida R., Moriya T., Akiyama M., Shibata S. (2001). Restricted feeding entrains liver clock without participation of the suprachiasmatic nucleus. Genes Cells.

[262] Damiola F., Le Minh N., Preitner N., Kornmann B., Fleury-Olela F., Schibler U. (2000). Restricted feeding uncouples circadian oscillators in peripheral tissues from the central pacemaker in the suprachiasmatic nucleus. Genes Dev..

[263] Morris C.J., Yang J.N., Garcia J.I., Myers S., Bozzi I., Wang W., Buxton O.M., Shea S.A., Scheer F.A. (2015). Endogenous circadian system and circadian misalignment impact glucose tolerance via separate mechanisms in humans. Proc. Natl. Acad. Sci. USA.

[264] Mazri F.H., Manaf Z.A., Shahar S., Mat Ludin A.F. (2019). The Association between Chronotype and Dietary Pattern among Adults: A Scoping Review. Int. J. Environ. Res. Public Health.

[265] Eckel-Mahan K.L., Patel V.R., de Mateo S., Orozco-Solis R., Ceglia N.J., Sahar S., Dilag-Penilla S.A., Dyar K.A., Baldi P., Sassone-Corsi P. (2013). Reprogramming of the circadian clock by nutritional challenge. Cell.

[266] Solomon T.P., Chambers E.S., Jeukendrup A.E., Toogood A.A., Blannin A.K. (2008). The effect of feeding frequency on insulin and ghrelin responses in human subjects. Br. J. Nutr..

[267] Hirao A., Tahara Y., Kimura I., Shibata S. (2009). A balanced diet is necessary for proper entrainment signals of the mouse liver clock. PLoS ONE.

[268] Kuroda H., Tahara Y., Saito K., Ohnishi N., Kubo Y., Seo Y., Otsuka M., Fuse Y., Ohura Y., Hirao A. (2012). Meal frequency patterns determine the phase of mouse peripheral circadian clocks. Sci. Rep..

[269] Hirao A., Nagahama H., Tsuboi T., Hirao M., Tahara Y., Shibata S. (2010). Combination of starvation interval and food volume determines the phase of liver circadian rhythm in Per2::Luc knock-in mice under two meals per day feeding. Am. J. Physiol. Gastrointest. Liver Physiol..

[270] Oike H., Oishi K., Kobori M. (2014). Nutrients, Clock Genes, and Chrononutrition. Curr. Nutr. Rep..

[271] Bo S., Broglio F., Settanni F., Parasiliti Caprino M., Ianniello A., Mengozzi G., De Francesco A., Fadda M., Fedele D., Guggino A. (2017). Effects of meal timing on changes in circulating epinephrine, norepinephrine, and acylated ghrelin concentrations: A pilot study. Nutr. Diabetes.

[272] Takahashi M., Ozaki M., Kang M.I., Sasaki H., Fukazawa M., Iwakami T., Lim P.J., Kim H.K., Aoyama S., Shibata S. (2018). Effects of Meal Timing on Postprandial Glucose Metabolism and Blood Metabolites in Healthy Adults. Nutrients.

[273] Chen H.J., Chuang S.Y., Chang H.Y., Pan W.H. (2019). Energy intake at different times of the day: Its association with elevated total and LDL cholesterol levels. Nutr. Metab. Cardiovasc. Dis..

[274] Xue P., Tan X., Tang X., Benedict C. (2021). Chronotype preference and glycemic control in type 2 diabetes. Sleep.

[275] Maki K.C., Phillips-Eakley A.K., Smith K.N. (2016). The Effects of Breakfast Consumption and Composition on Metabolic Wellness with a Focus on Carbohydrate Metabolism. Adv. Nutr..

[276] Dhurandhar E.J. (2016). True, true, unrelated? A review of recent evidence for a causal influence of breakfast on obesity. Curr. Opin. Endocrinol. Diabetes Obes..

[277] Ballon A., Neuenschwander M., Schlesinger S. (2019). Breakfast Skipping Is Associated with Increased Risk of Type 2 Diabetes among Adults: A Systematic Review and Meta-Analysis of Prospective Cohort Studies. J. Nutr..

[278] Mekary R.A., Giovannucci E., Willett W.C., van Dam R.M., Hu F.B. (2012). Eating patterns and type 2 diabetes risk in men: Breakfast omission, eating frequency, and snacking. Am. J. Clin. Nutr..

[279] Odegaard A.O., Jacobs D.R., Steffen L.M., Van Horn L., Ludwig D.S., Pereira M.A. (2013). Breakfast frequency and development of metabolic risk. Diabetes Care.

[280] Betts J.A., Richardson J.D., Chowdhury E.A., Holman G.D., Tsintzas K., Thompson D. (2014). The causal role of breakfast in energy balance and health: A randomized controlled trial in lean adults. Am. J. Clin. Nutr..

[281] Bonnet J.P., Cardel M.I., Cellini J., Hu F.B., Guasch-Ferre M. (2020). Breakfast Skipping, Body Composition, and Cardiometabolic Risk: A Systematic Review and Meta-Analysis of Randomized Trials. Obesity.

[282] Rosi A., Martini D., Scazzina F., Dall’Aglio E., Leonardi R., Monti L., Fasano F., Di Dio C., Riggio L., Brighenti F. (2018). Nature and Cognitive Perception of 4 Different Breakfast Meals Influence Satiety-Related Sensations and Postprandial Metabolic Responses but Have Little Effect on Food Choices and Intake Later in the Day in a Randomized Crossover Trial in Healthy Men. J. Nutr..

[283] Chowdhury E.A., Richardson J.D., Holman G.D., Tsintzas K., Thompson D., Betts J.A. (2016). The causal role of breakfast in energy balance and health: A randomized controlled trial in obese adults. Am. J. Clin. Nutr..

[284] Reutrakul S., Hood M.M., Crowley S.J., Morgan M.K., Teodori M., Knutson K.L. (2014). The relationship between breakfast skipping, chronotype, and glycemic control in type 2 diabetes. Chronobiol. Int..

[285] Jakubowicz D., Wainstein J., Landau Z., Raz I., Ahren B., Chapnik N., Ganz T., Menaged M., Barnea M., Bar-Dayan Y. (2017). Influences of Breakfast on Clock Gene Expression and Postprandial Glycemia in Healthy Individuals and Individuals With Diabetes: A Randomized Clinical Trial. Diabetes Care.

[286] Nas A., Mirza N., Hagele F., Kahlhofer J., Keller J., Rising R., Kufer T.A., Bosy-Westphal A. (2017). Impact of breakfast skipping compared with dinner skipping on regulation of energy balance and metabolic risk. Am. J. Clin. Nutr..

[287] Kobayashi F., Ogata H., Omi N., Nagasaka S., Yamaguchi S., Hibi M., Tokuyama K. (2014). Effect of breakfast skipping on diurnal variation of energy metabolism and blood glucose. Obes. Res. Clin Pr..

[288] Nakajima K., Suwa K. (2015). Association of hyperglycemia in a general Japanese population with late-night-dinner eating alone, but not breakfast skipping alone. J. Diabetes Metab. Disord..

[289] Morgan L.M., Aspostolakou F., Wright J., Gama R. (1999). Diurnal variations in peripheral insulin resistance and plasma non-esterified fatty acid concentrations: A possible link?. Ann. Clin. Biochem..

[290] Livesey G., Taylor R., Livesey H.F., Buyken A.E., Jenkins D.J.A., Augustin L.S.A., Sievenpiper J.L., Barclay A.W., Liu S., Wolever T.M.S. (2019). Dietary Glycemic Index and Load and the Risk of Type 2 Diabetes: Assessment of Causal Relations. Nutrients.

[291] Toh D.W.K., Koh E.S., Kim J.E. (2020). Lowering breakfast glycemic index and glycemic load attenuates postprandial glycemic response: A systematically searched meta-analysis of randomized controlled trials. Nutrition.

[292] Kim H., Stote K.S., Behall K.M., Spears K., Vinyard B., Conway J.M. (2009). Glucose and insulin responses to whole grain breakfasts varying in soluble fiber, beta-glucan: A dose response study in obese women with increased risk for insulin resistance. Eur. J. Nutr..

[293] Papakonstantinou E., Chaloulos P., Papalexi A., Mandala I. (2018). Effects of bran size and carob seed flour of optimized bread formulas on glycemic responses in humans: A randomized clinical trial. J. Funct. Foods.

[294] Papakonstantinou E., Orfanakos N., Farajian P., Kapetanakou A.E., Makariti I.P., Grivokostopoulos N., Ha M.A., Skandamis P.N. (2017). Short-term effects of a low glycemic index carob-containing snack on energy intake, satiety, and glycemic response in normal-weight, healthy adults: Results from two randomized trials. Nutrition.

[295] Gourdomichali T., Papakonstantinou E. (2018). Short-term effects of six Greek honey varieties on glycemic response: A randomized clinical trial in healthy subjects. Eur. J. Clin. Nutr..

[296] Jakubowicz D., Wainstein J., Landau Z., Ahren B., Barnea M., Bar-Dayan Y., Froy O. (2017). High-energy breakfast based on whey protein reduces body weight, postprandial glycemia and HbA1C in Type 2 diabetes. J. Nutr. Biochem..

[297] Jakubowicz D., Froy O., Wainstein J., Boaz M. (2012). Meal timing and composition influence ghrelin levels, appetite scores and weight loss maintenance in overweight and obese adults. Steroids.

[298] Neumann B.L., Dunn A., Johnson D., Adams J.D., Baum J.I. (2016). Breakfast Macronutrient Composition Influences Thermic Effect of Feeding and Fat Oxidation in Young Women Who Habitually Skip Breakfast. Nutrients.

[299] Pedersen E., Lange K., Clifton P. (2016). Effect of carbohydrate restriction in the first meal after an overnight fast on glycemic control in people with type 2 diabetes: A randomized trial. Am. J. Clin. Nutr..

[300] Rabinovitz H.R., Boaz M., Ganz T., Jakubowicz D., Matas Z., Madar Z., Wainstein J. (2014). Big breakfast rich in protein and fat improves glycemic control in type 2 diabetics. Obesity.

[301] Jakubowicz D., Wainstein J., Ahren B., Bar-Dayan Y., Landau Z., Rabinovitz H.R., Froy O. (2015). High-energy breakfast with low-energy dinner decreases overall daily hyperglycaemia in type 2 diabetic patients: A randomised clinical trial. Diabetologia.

[302] Papakonstantinou E., Triantafillidou D., Panagiotakos D.B., Iraklianou S., Berdanier C.D., Zampelas A. (2010). A high protein low fat meal does not influence glucose and insulin responses in obese individuals with or without type 2 diabetes. J. Hum. Nutr. Diet..

[303] Dong J.Y., Zhang Z.L., Wang P.Y., Qin L.Q. (2013). Effects of high-protein diets on body weight, glycaemic control, blood lipids and blood pressure in type 2 diabetes: Meta-analysis of randomised controlled trials. Br. J. Nutr..

[304] Ericson U., Hellstrand S., Brunkwall L., Schulz C.A., Sonestedt E., Wallstrom P., Gullberg B., Wirfalt E., Orho-Melander M. (2015). Food sources of fat may clarify the inconsistent role of dietary fat intake for incidence of type 2 diabetes. Am. J. Clin. Nutr..

[305] Guasch-Ferre M., Becerra-Tomas N., Ruiz-Canela M., Corella D., Schroder H., Estruch R., Ros E., Aros F., Gomez-Gracia E., Fiol M. (2017). Total and subtypes of dietary fat intake and risk of type 2 diabetes mellitus in the Prevencion con Dieta Mediterranea (PREDIMED) study. Am. J. Clin. Nutr..

[306] Wu T., Fu O., Yao L., Sun L., Zhuge F., Fu Z. (2012). Differential responses of peripheral circadian clocks to a short-term feeding stimulus. Mol. Biol. Rep..

[307] Tsuchida Y., Hata S., Sone Y. (2013). Effects of a late supper on digestion and the absorption of dietary carbohydrates in the following morning. J. Physiol. Anthr..

[308] Grant C.L., Coates A.M., Dorrian J., Kennaway D.J., Wittert G.A., Heilbronn L.K., Pajcin M., Della Vedova C., Gupta C.C., Banks S. (2017). Timing of food intake during simulated night shift impacts glucose metabolism: A controlled study. Chronobiol. Int..

[309] Bandin C., Scheer F.A., Luque A.J., Avila-Gandia V., Zamora S., Madrid J.A., Gomez-Abellan P., Garaulet M. (2015). Meal timing affects glucose tolerance, substrate oxidation and circadian-related variables: A randomized, crossover trial. Int. J. Obes..

[310] Lopez-Minguez J., Saxena R., Bandin C., Scheer F.A., Garaulet M. (2018). Late dinner impairs glucose tolerance in MTNR1B risk allele carriers: A randomized, cross-over study. Clin. Nutr..

[311] Ahmed S.H., Chowdhury T.A., Hussain S., Syed A., Karamat A., Helmy A., Waqar S., Ali S., Dabhad A., Seal S.T. (2020). Ramadan and Diabetes: A Narrative Review and Practice Update. Diabetes.

[312] Garaulet M., Gomez-Abellan P., Alburquerque-Bejar J.J., Lee Y.C., Ordovas J.M., Scheer F.A. (2013). Timing of food intake predicts weight loss effectiveness. Int. J. Obes..

[313] Imai S., Kajiyama S., Hashimoto Y., Nitta A., Miyawaki T., Matsumoto S., Ozasa N., Tanaka M., Kajiyama S., Fukui M. (2018). Consuming snacks mid-afternoon compared with just after lunch improves mean amplitude of glycaemic excursions in patients with type 2 diabetes: A randomized crossover clinical trial. Diabetes Metab..

[314] Kessler K., Hornemann S., Petzke K.J., Kemper M., Kramer A., Pfeiffer A.F., Pivovarova O., Rudovich N. (2017). The effect of diurnal distribution of carbohydrates and fat on glycaemic control in humans: A randomized controlled trial. Sci. Rep..

[315] Gibbs M., Harrington D., Starkey S., Williams P., Hampton S. (2014). Diurnal postprandial responses to low and high glycaemic index mixed meals. Clin. Nutr..

[316] Scheer F.A., Morris C.J., Shea S.A. (2013). The internal circadian clock increases hunger and appetite in the evening independent of food intake and other behaviors. Obesity.

[317] Jakubowicz D., Barnea M., Wainstein J., Froy O. (2013). High caloric intake at breakfast vs. dinner differentially influences weight loss of overweight and obese women. Obesity.

[318] Chen H.J., Wang Y., Cheskin L.J. (2016). Relationship between frequency of eating and cardiovascular disease mortality in U.S. adults: The NHANES III follow-up study. Ann. Epidemiol..

[319] Mattson M.P. (2005). The need for controlled studies of the effects of meal frequency on health. Lancet.

[320] Kahleova H., Belinova L., Malinska H., Oliyarnyk O., Trnovska J., Skop V., Kazdova L., Dezortova M., Hajek M., Tura A. (2014). Eating two larger meals a day (breakfast and lunch) is more effective than six smaller meals in a reduced-energy regimen for patients with type 2 diabetes: A randomised crossover study. Diabetologia.

[321] Jenkins D.J., Wolever T.M., Vuksan V., Brighenti F., Cunnane S.C., Rao A.V., Jenkins A.L., Buckley G., Patten R., Singer W. (1989). Nibbling versus gorging: Metabolic advantages of increased meal frequency. N. Engl. J. Med..

[322] Seagle H.M., Strain G.W., Makris A., Reeves R.S., American Dietetic Association (2009). Position of the American Dietetic Association: Weight management. J. Am. Diet. Assoc..

[323] Papakonstantinou E., Kechribari I., Mitrou P., Trakakis E., Vassiliadi D., Georgousopoulou E., Zampelas A., Kontogianni M.D., Dimitriadis G. (2016). Effect of meal frequency on glucose and insulin levels in women with polycystic ovary syndrome: A randomised trial. Eur. J. Clin. Nutr..

[324] Papakonstantinou E., Kontogianni M.D., Mitrou P., Magriplis E., Vassiliadi D., Nomikos T., Lambadiari V., Georgousopoulou E., Dimitriadis G. (2018). Effects of 6 vs 3 eucaloric meal patterns on glycaemic control and satiety in people with impaired glucose tolerance or overt type 2 diabetes: A randomized trial. Diabetes Metab..

[325] Jenkins D.J., Ocana A., Jenkins A.L., Wolever T.M., Vuksan V., Katzman L., Hollands M., Greenberg G., Corey P., Patten R. (1992). Metabolic advantages of spreading the nutrient load: Effects of increased meal frequency in non-insulin-dependent diabetes. Am. J. Clin. Nutr..

[326] Jakubowicz D., Landau Z., Tsameret S., Wainstein J., Raz I., Ahren B., Chapnik N., Barnea M., Ganz T., Menaged M. (2019). Reduction in Glycated Hemoglobin and Daily Insulin Dose Alongside Circadian Clock Upregulation in Patients With Type 2 Diabetes Consuming a Three-Meal Diet: A Randomized Clinical Trial. Diabetes Care.

[327] Kahleova H., Lloren J.I., Mashchak A., Hill M., Fraser G.E. (2017). Meal Frequency and Timing Are Associated with Changes in Body Mass Index in Adventist Health Study 2. J. Nutr..

[328] Farshchi H.R., Taylor M.A., Macdonald I.A. (2004). Regular meal frequency creates more appropriate insulin sensitivity and lipid profiles compared with irregular meal frequency in healthy lean women. Eur. J. Clin. Nutr..

[329] Farshchi H.R., Taylor M.A., Macdonald I.A. (2005). Beneficial metabolic effects of regular meal frequency on dietary thermogenesis, insulin sensitivity, and fasting lipid profiles in healthy obese women. Am. J. Clin. Nutr..

[330] Bertelsen J., Christiansen C., Thomsen C., Poulsen P.L., Vestergaard S., Steinov A., Rasmussen L.H., Rasmussen O., Hermansen K. (1993). Effect of meal frequency on blood glucose, insulin, and free fatty acids in NIDDM subjects. Diabetes Care.

[331] Arnold L., Mann J.I., Ball M.J. (1997). Metabolic effects of alterations in meal frequency in type 2 diabetes. Diabetes Care.

[332] Arnold L.M., Ball M.J., Duncan A.W., Mann J. (1993). Effect of isoenergetic intake of three or nine meals on plasma lipoproteins and glucose metabolism. Am. J. Clin. Nutr..

[333] Salehi M., Kazemi A., Hasan Zadeh J. (2014). The effects of 6 isocaloric meals pattern on blood lipid profile, glucose, hemoglobin a1c, insulin and malondialdehyde in type 2 diabetic patients: A randomized clinical trial. Iran J. Med. Sci..

[334] Franz M.J., VanWormer J.J., Crain A.L., Boucher J.L., Histon T., Caplan W., Bowman J.D., Pronk N.P. (2007). Weight-loss outcomes: A systematic review and meta-analysis of weight-loss clinical trials with a minimum 1-year follow-up. J. Am. Diet. Assoc..

[335] Chaix A., Zarrinpar A., Miu P., Panda S. (2014). Time-restricted feeding is a preventative and therapeutic intervention against diverse nutritional challenges. Cell Metab..

[336] Hatori M., Vollmers C., Zarrinpar A., DiTacchio L., Bushong E.A., Gill S., Leblanc M., Chaix A., Joens M., Fitzpatrick J.A. (2012). Time-restricted feeding without reducing caloric intake prevents metabolic diseases in mice fed a high-fat diet. Cell Metab..

[337] Adamovich Y., Rousso-Noori L., Zwighaft Z., Neufeld-Cohen A., Golik M., Kraut-Cohen J., Wang M., Han X., Asher G. (2014). Circadian clocks and feeding time regulate the oscillations and levels of hepatic triglycerides. Cell Metab..

[338] Paoli A., Tinsley G., Bianco A., Moro T. (2019). The Influence of Meal Frequency and Timing on Health in Humans: The Role of Fasting. Nutrients.

[339] Veech R.L., Bradshaw P.C., Clarke K., Curtis W., Pawlosky R., King M.T. (2017). Ketone bodies mimic the life span extending properties of caloric restriction. IUBMB Life.

[340] Di Francesco A., Di Germanio C., Bernier M., de Cabo R. (2018). A time to fast. Science.

[341] Hawley J.A., Sassone-Corsi P., Zierath J.R. (2020). Chrono-nutrition for the prevention and treatment of obesity and type 2 diabetes: From mice to men. Diabetologia.

[342] Hutchison A.T., Regmi P., Manoogian E.N.C., Fleischer J.G., Wittert G.A., Panda S., Heilbronn L.K. (2019). Time-Restricted Feeding Improves Glucose Tolerance in Men at Risk for Type 2 Diabetes: A Randomized Crossover Trial. Obesity.

[343] Wehrens S.M.T., Christou S., Isherwood C., Middleton B., Gibbs M.A., Archer S.N., Skene D.J., Johnston J.D. (2017). Meal Timing Regulates the Human Circadian System. Curr. Biol..

[344] Jamshed H., Beyl R.A., Della Manna D.L., Yang E.S., Ravussin E., Peterson C.M. (2019). Early Time-Restricted Feeding Improves 24-Hour Glucose Levels and Affects Markers of the Circadian Clock, Aging, and Autophagy in Humans. Nutrients.

[345] Wilkinson M.J., Manoogian E.N.C., Zadourian A., Lo H., Fakhouri S., Shoghi A., Wang X., Fleischer J.G., Navlakha S., Panda S. (2020). Ten-Hour Time-Restricted Eating Reduces Weight, Blood Pressure, and Atherogenic Lipids in Patients with Metabolic Syndrome. Cell Metab..

[346] Carter S., Clifton P.M., Keogh J.B. (2018). Effect of Intermittent Compared With Continuous Energy Restricted Diet on Glycemic Control in Patients With Type 2 Diabetes: A Randomized Noninferiority Trial. JAMA Netw Open.

[347] Carter S., Clifton P.M., Keogh J.B. (2019). The effect of intermittent compared with continuous energy restriction on glycaemic control in patients with type 2 diabetes: 24-month follow-up of a randomised noninferiority trial. Diabetes Res. Clin. Pr..

[348] Varady K.A., Bhutani S., Klempel M.C., Kroeger C.M., Trepanowski J.F., Haus J.M., Hoddy K.K., Calvo Y. (2013). Alternate day fasting for weight loss in normal weight and overweight subjects: A randomized controlled trial. Nutr. J..

[349] Catenacci V.A., Pan Z., Ostendorf D., Brannon S., Gozansky W.S., Mattson M.P., Martin B., MacLean P.S., Melanson E.L., Troy Donahoo W. (2016). A randomized pilot study comparing zero-calorie alternate-day fasting to daily caloric restriction in adults with obesity. Obesity.

[350] Trepanowski J.F., Kroeger C.M., Barnosky A., Klempel M.C., Bhutani S., Hoddy K.K., Gabel K., Freels S., Rigdon J., Rood J. (2017). Effect of Alternate-Day Fasting on Weight Loss, Weight Maintenance, and Cardioprotection Among Metabolically Healthy Obese Adults: A Randomized Clinical Trial. JAMA Intern. Med..

[351] Gabel K., Kroeger C.M., Trepanowski J.F., Hoddy K.K., Cienfuegos S., Kalam F., Varady K.A. (2019). Differential Effects of Alternate-Day Fasting Versus Daily Calorie Restriction on Insulin Resistance. Obesity.

[352] Hoddy K.K., Kroeger C.M., Trepanowski J.F., Barnosky A., Bhutani S., Varady K.A. (2014). Meal timing during alternate day fasting: Impact on body weight and cardiovascular disease risk in obese adults. Obesity.

[353] Harvie M.N., Pegington M., Mattson M.P., Frystyk J., Dillon B., Evans G., Cuzick J., Jebb S.A., Martin B., Cutler R.G. (2011). The effects of intermittent or continuous energy restriction on weight loss and metabolic disease risk markers: A randomized trial in young overweight women. Int. J. Obes..

[354] Harvie M., Wright C., Pegington M., McMullan D., Mitchell E., Martin B., Cutler R.G., Evans G., Whiteside S., Maudsley S. (2013). The effect of intermittent energy and carbohydrate restriction v. daily energy restriction on weight loss and metabolic disease risk markers in overweight women. Br. J. Nutr..

[355] Sutton E.F., Beyl R., Early K.S., Cefalu W.T., Ravussin E., Peterson C.M. (2018). Early Time-Restricted Feeding Improves Insulin Sensitivity, Blood Pressure, and Oxidative Stress Even without Weight Loss in Men with Prediabetes. Cell Metab..

[356] Parr E.B., Devlin B.L., Lim K.H.C., Moresi L.N.Z., Geils C., Brennan L., Hawley J.A. (2020). Time-Restricted Eating as a Nutrition Strategy for Individuals with Type 2 Diabetes: A Feasibility Study. Nutrients.

[357] Moro T., Tinsley G., Bianco A., Marcolin G., Pacelli Q.F., Battaglia G., Palma A., Gentil P., Neri M., Paoli A. (2016). Effects of eight weeks of time-restricted feeding (16/8) on basal metabolism, maximal strength, body composition, inflammation, and cardiovascular risk factors in resistance-trained males. J. Transl. Med..

[358] Raynor H.A., Li F., Cardoso C. (2018). Daily pattern of energy distribution and weight loss. Physiol. Behav..

[359] Rothschild J., Hoddy K.K., Jambazian P., Varady K.A. (2014). Time-restricted feeding and risk of metabolic disease: A review of human and animal studies. Nutr. Rev..

[360] Tinsley G.M., La Bounty P.M. (2015). Effects of intermittent fasting on body composition and clinical health markers in humans. Nutr. Rev..

[361] Carlson O., Martin B., Stote K.S., Golden E., Maudsley S., Najjar S.S., Ferrucci L., Ingram D.K., Longo D.L., Rumpler W.V. (2007). Impact of reduced meal frequency without caloric restriction on glucose regulation in healthy, normal-weight middle-aged men and women. Metabolism.

[362] Stote K.S., Baer D.J., Spears K., Paul D.R., Harris G.K., Rumpler W.V., Strycula P., Najjar S.S., Ferrucci L., Ingram D.K. (2007). A controlled trial of reduced meal frequency without caloric restriction in healthy, normal-weight, middle-aged adults. Am. J. Clin. Nutr..

[363] Williams K.V., Mullen M.L., Kelley D.E., Wing R.R. (1998). The effect of short periods of caloric restriction on weight loss and glycemic control in type 2 diabetes. Diabetes Care.

[364] Carter S., Clifton P.M., Keogh J.B. (2016). The effects of intermittent compared to continuous energy restriction on glycaemic control in type 2 diabetes; a pragmatic pilot trial. Diabetes Res. Clin. Pr..

[365] Qin B., Nagasaki M., Ren M., Bajotto G., Oshida Y., Sato Y. (2003). Cinnamon extract (traditional herb) potentiates in vivo insulin-regulated glucose utilization via enhancing insulin signaling in rats. Diabetes Res. Clin. Pr..

[366] Jarvill-Taylor K.J., Anderson R.A., Graves D.J. (2001). A hydroxychalcone derived from cinnamon functions as a mimetic for insulin in 3T3-L1 adipocytes. J. Am. Coll. Nutr..

[367] Broadhurst C.L., Polansky M.M., Anderson R.A. (2000). Insulin-like biological activity of culinary and medicinal plant aqueous extracts in vitro. J. Agric. Food Chem..

[368] Liu Y., Cotillard A., Vatier C., Bastard J.P., Fellahi S., Stevant M., Allatif O., Langlois C., Bieuvelet S., Brochot A. (2015). A Dietary Supplement Containing Cinnamon, Chromium and Carnosine Decreases Fasting Plasma Glucose and Increases Lean Mass in Overweight or Obese Pre-Diabetic Subjects: A Randomized, Placebo-Controlled Trial. PLoS ONE.

[369] Zare R., Nadjarzadeh A., Zarshenas M.M., Shams M., Heydari M. (2019). Efficacy of cinnamon in patients with type II diabetes mellitus: A randomized controlled clinical trial. Clin. Nutr..

[370] Gupta Jain S., Puri S., Misra A., Gulati S., Mani K. (2017). Effect of oral cinnamon intervention on metabolic profile and body composition of Asian Indians with metabolic syndrome: A randomized double -blind control trial. Lipids Health Dis..

[371] Beejmohun V., Peytavy-Izard M., Mignon C., Muscente-Paque D., Deplanque X., Ripoll C., Chapal N. (2014). Acute effect of Ceylon cinnamon extract on postprandial glycemia: Alpha-amylase inhibition, starch tolerance test in rats, and randomized crossover clinical trial in healthy volunteers. BMC Complement. Altern. Med..

[372] Lu T., Sheng H., Wu J., Cheng Y., Zhu J., Chen Y. (2012). Cinnamon extract improves fasting blood glucose and glycosylated hemoglobin level in Chinese patients with type 2 diabetes. Nutr. Res..

[373] Akilen R., Tsiami A., Devendra D., Robinson N. (2010). Glycated haemoglobin and blood pressure-lowering effect of cinnamon in multi-ethnic Type 2 diabetic patients in the UK: A randomized, placebo-controlled, double-blind clinical trial. Diabet. Med. A J. Br. Diabet. Assoc..

[374] Mang B., Wolters M., Schmitt B., Kelb K., Lichtinghagen R., Stichtenoth D.O., Hahn A. (2006). Effects of a cinnamon extract on plasma glucose, HbA, and serum lipids in diabetes mellitus type 2. Eur. J. Clin. Investig..

[375] Khan A., Safdar M., Ali Khan M.M., Khattak K.N., Anderson R.A. (2003). Cinnamon improves glucose and lipids of people with type 2 diabetes. Diabetes Care.

[376] Solomon T.P., Blannin A.K. (2009). Changes in glucose tolerance and insulin sensitivity following 2 weeks of daily cinnamon ingestion in healthy humans. Eur. J. Appl. Physiol..

[377] Talaei B., Amouzegar A., Sahranavard S., Hedayati M., Mirmiran P., Azizi F. (2017). Effects of Cinnamon Consumption on Glycemic Indicators, Advanced Glycation End Products, and Antioxidant Status in Type 2 Diabetic Patients. Nutrients.

[378] Whitfield P., Parry-Strong A., Walsh E., Weatherall M., Krebs J.D. (2016). The effect of a cinnamon-, chromium- and magnesium-formulated honey on glycaemic control, weight loss and lipid parameters in type 2 diabetes: An open-label cross-over randomised controlled trial. Eur. J. Nutr..

[379] Markey O., McClean C.M., Medlow P., Davison G.W., Trinick T.R., Duly E., Shafat A. (2011). Effect of cinnamon on gastric emptying, arterial stiffness, postprandial lipemia, glycemia, and appetite responses to high-fat breakfast. Cardiovasc. Diabetol..

[380] Wainstein J., Stern N., Heller S., Boaz M. (2011). Dietary cinnamon supplementation and changes in systolic blood pressure in subjects with type 2 diabetes. J. Med. Food.

[381] Blevins S.M., Leyva M.J., Brown J., Wright J., Scofield R.H., Aston C.E. (2007). Effect of cinnamon on glucose and lipid levels in non insulin-dependent type 2 diabetes. Diabetes Care.

[382] Vanschoonbeek K., Thomassen B.J., Senden J.M., Wodzig W.K., van Loon L.J. (2006). Cinnamon supplementation does not improve glycemic control in postmenopausal type 2 diabetes patients. J. Nutr..

[383] Liljeberg H.G., Bjorck I.M. (1996). Delayed gastric emptying rate as a potential mechanism for lowered glycemia after eating sourdough bread: Studies in humans and rats using test products with added organic acids or an organic salt. Am. J. Clin. Nutr..

[384] Marco M.L., Sanders M.E., Ganzle M., Arrieta M.C., Cotter P.D., De Vuyst L., Hill C., Holzapfel W., Lebeer S., Merenstein D. (2021). The International Scientific Association for Probiotics and Prebiotics (ISAPP) consensus statement on fermented foods. Nat. Rev. Gastroenterol. Hepatol..

[385] Capurso A., Capurso C. (2020). The Mediterranean way: Why elderly people should eat wholewheat sourdough bread-a little known component of the Mediterranean diet and healthy food for elderly adults. Aging Clin. Exp. Res..

[386] Liljeberg H., Bjorck I. (1998). Delayed gastric emptying rate may explain improved glycaemia in healthy subjects to a starchy meal with added vinegar. Eur. J. Clin. Nutr..

[387] Liljeberg H.G., Lonner C.H., Bjorck I.M. (1995). Sourdough fermentation or addition of organic acids or corresponding salts to bread improves nutritional properties of starch in healthy humans. J. Nutr..

[388] Poutanen K., Flander L., Katina K. (2009). Sourdough and cereal fermentation in a nutritional perspective. Food Microbiol..

[389] Battilana P., Ornstein K., Minehira K., Schwarz J.M., Acheson K., Schneiter P., Burri J., Jequier E., Tappy L. (2001). Mechanisms of action of beta-glucan in postprandial glucose metabolism in healthy men. Eur. J. Clin. Nutr..

[390] Scazzina F., Del Rio D., Pellegrini N., Brighenti F. (2009). Sourdough bread: Starch digestibility and postprandial glycemic response. J. Cereal Sci..

[391] Ostman E.M., Liljeberg Elmstahl H.G., Bjorck I.M. (2001). Inconsistency between glycemic and insulinemic responses to regular and fermented milk products. Am. J. Clin. Nutr..

[392] Brighenti F., Benini L., Del Rio D., Casiraghi C., Pellegrini N., Scazzina F., Jenkins D.J., Vantini I. (2006). Colonic fermentation of indigestible carbohydrates contributes to the second-meal effect. Am. J. Clin. Nutr..

[393] Johnston C.S., Gaas C.A. (2006). Vinegar: Medicinal uses and antiglycemic effect. MedGenMed.

[394] Salbe A.D., Johnston C.S., Buyukbese M.A., Tsitouras P.D., Harman S.M. (2009). Vinegar lacks antiglycemic action on enteral carbohydrate absorption in human subjects. Nutr. Res..

[395] Ostman E., Granfeldt Y., Persson L., Bjorck I. (2005). Vinegar supplementation lowers glucose and insulin responses and increases satiety after a bread meal in healthy subjects. Eur. J. Clin. Nutr..

[396] Brighenti F., Castellani G., Benini L., Casiraghi M.C., Leopardi E., Crovetti R., Testolin G. (1995). Effect of neutralized and native vinegar on blood glucose and acetate responses to a mixed meal in healthy subjects. Eur. J. Clin. Nutr..

[397] Johnston C.S., Buller A.J. (2005). Vinegar and peanut products as complementary foods to reduce postprandial glycemia. J. Am. Diet. Assoc..

[398] Mitrou P., Petsiou E., Papakonstantinou E., Maratou E., Lambadiari V., Dimitriadis P., Spanoudi F., Raptis S.A., Dimitriadis G. (2015). Vinegar Consumption Increases Insulin-Stimulated Glucose Uptake by the Forearm Muscle in Humans with Type 2 Diabetes. J. Diabetes Res..

[399] Liatis S., Grammatikou S., Poulia K.A., Perrea D., Makrilakis K., Diakoumopoulou E., Katsilambros N. (2010). Vinegar reduces postprandial hyperglycaemia in patients with type II diabetes when added to a high, but not to a low, glycaemic index meal. Eur. J. Clin. Nutr..

[400] Mitrou P., Raptis A.E., Lambadiari V., Boutati E., Petsiou E., Spanoudi F., Papakonstantinou E., Maratou E., Economopoulos T., Dimitriadis G. (2010). Vinegar decreases postprandial hyperglycemia in patients with type 1 diabetes. Diabetes Care.

[401] White A.M., Johnston C.S. (2007). Vinegar ingestion at bedtime moderates waking glucose concentrations in adults with well-controlled type 2 diabetes. Diabetes Care.

[402] Johnston C.S., Kim C.M., Buller A.J. (2004). Vinegar improves insulin sensitivity to a high-carbohydrate meal in subjects with insulin resistance or type 2 diabetes. Diabetes Care.

[403] Mitrou P., Petsiou E., Papakonstantinou E., Maratou E., Lambadiari V., Dimitriadis P., Spanoudi F., Raptis S.A., Dimitriadis G. (2015). The role of acetic acid on glucose uptake and blood flow rates in the skeletal muscle in humans with impaired glucose tolerance. Eur. J. Clin. Nutr..

[404] Van Dijk J.W., Tummers K., Hamer H.M., van Loon L.J. (2012). Vinegar co-ingestion does not improve oral glucose tolerance in patients with type 2 diabetes. J. Diabetes Complicat..

[405] Hlebowicz J., Darwiche G., Bjorgell O., Almer L.O. (2007). Effect of apple cider vinegar on delayed gastric emptying in patients with type 1 diabetes mellitus: A pilot study. BMC Gastroenterol..

[406] Ogawa N., Satsu H., Watanabe H., Fukaya M., Tsukamoto Y., Miyamoto Y., Shimizu M. (2000). Acetic acid suppresses the increase in disaccharidase activity that occurs during culture of caco-2 cells. J. Nutr..

[407] Siddiqui F.J., Assam P.N., de Souza N.N., Sultana R., Dalan R., Chan E.S. (2018). Diabetes Control: Is Vinegar a Promising Candidate to Help Achieve Targets?. J. Evid. Based Integr. Med..

[408] Savaiano D.A., Hutkins R.W. (2021). Yogurt, cultured fermented milk, and health: A systematic review. Nutr. Rev..

[409] Grom L.C., Coutinho N.M., Guimaraes J.T., Balthazar C.F., Silva R., Rocha R.S., Freitas M.Q., Duarte M.C.K.H., Pimentel T.C., Esmerino E.A. (2020). Probiotic dairy foods and postprandial glycemia: A mini-review. Trends Food Sci. Technol..

[410] Salles B.I.M., Cioffi D., Ferreira S.R.G. (2020). Probiotics supplementation and insulin resistance: A systematic review. Diabetol. Metab. Syndr..

[411] Li L., Li P., Xu L. (2021). Assessing the effects of inulin-type fructan intake on body weight, blood glucose, and lipid profile: A systematic review and meta-analysis of randomized controlled trials. Food Sci. Nutr..

[412] Wang L., Yang H., Huang H., Zhang C., Zuo H.X., Xu P., Niu Y.M., Wu S.S. (2019). Inulin-type fructans supplementation improves glycemic control for the prediabetes and type 2 diabetes populations: Results from a GRADE-assessed systematic review and dose-response meta-analysis of 33 randomized controlled trials. J. Transl. Med..

[413] Palma-Duran S.A., Vlassopoulos A., Lean M., Govan L., Combet E. (2017). Nutritional intervention and impact of polyphenol on glycohemoglobin (HbA1c) in non-diabetic and type 2 diabetic subjects: Systematic review and meta-analysis. Crit. Rev. Food Sci. Nutr..

[414] Kim Y., Keogh J.B., Clifton P.M. (2016). Polyphenols and Glycemic Control. Nutrients.

[415] Teoh S.L., Lai N.M., Vanichkulpitak P., Vuksan V., Ho H., Chaiyakunapruk N. (2018). Clinical evidence on dietary supplementation with chia seed (Salvia hispanica L.): A systematic review and meta-analysis. Nutr. Rev..

[416] Nowrouzi-Sohrabi P., Hassanipour S., Sisakht M., Daryabeygi-Khotbehsara R., Savardashtaki A., Fathalipour M. (2020). The effectiveness of pistachio on glycemic control and insulin sensitivity in patients with type 2 diabetes, prediabetes and metabolic syndrome: A systematic review and meta-analysis. Diabetes Metab. Syndr.

[417] Bullo M., Juanola-Falgarona M., Hernandez-Alonso P., Salas-Salvado J. (2015). Nutrition attributes and health effects of pistachio nuts. Br. J. Nutr..

[418] Kendall C.W., Josse A.R., Esfahani A., Jenkins D.J. (2011). The impact of pistachio intake alone or in combination with high-carbohydrate foods on post-prandial glycemia. Eur. J. Clin. Nutr..

[419] Kendall C.W., West S.G., Augustin L.S., Esfahani A., Vidgen E., Bashyam B., Sauder K.A., Campbell J., Chiavaroli L., Jenkins A.L. (2014). Acute effects of pistachio consumption on glucose and insulin, satiety hormones and endothelial function in the metabolic syndrome. Eur. J. Clin. Nutr..

[420] Moon J.H., Lee J.Y., Kang S.B., Park J.S., Lee B.W., Kang E.S., Ahn C.W., Lee H.C., Cha B.S. (2010). Dietary monounsaturated fatty acids but not saturated fatty acids preserve the insulin signaling pathway via IRS-1/PI3K in rat skeletal muscle. Lipids.

[421] Schwingshackl L., Strasser B., Hoffmann G. (2011). Effects of monounsaturated fatty acids on glycaemic control in patients with abnormal glucose metabolism: A systematic review and meta-analysis. Ann. Nutr. Metab..

[422] Li S.C., Liu Y.H., Liu J.F., Chang W.H., Chen C.M., Chen C.Y. (2011). Almond consumption improved glycemic control and lipid profiles in patients with type 2 diabetes mellitus. Metabolism.

[423] Ojo O., Wang X.H., Ojo O.O., Adegboye A.R.A. (2021). The Effects of Almonds on Gut Microbiota, Glycometabolism, and Inflammatory Markers in Patients with Type 2 Diabetes: A Systematic Review and Meta-Analysis of Randomised Controlled Trials. Nutrients.

[424] Tindall A.M., Johnston E.A., Kris-Etherton P.M., Petersen K.S. (2019). The effect of nuts on markers of glycemic control: A systematic review and meta-analysis of randomized controlled trials. Am. J. Clin. Nutr..

[425] Ma Y., Njike V.Y., Millet J., Dutta S., Doughty K., Treu J.A., Katz D.L. (2010). Effects of walnut consumption on endothelial function in type 2 diabetic subjects: A randomized controlled crossover trial. Diabetes Care.

[426] Neale E.P., Guan V., Tapsell L.C., Probst Y.C. (2020). Effect of walnut consumption on markers of blood glucose control: A systematic review and meta-analysis. Br. J. Nutr..

[427] Amirani E., Milajerdi A., Reiner Z., Mirzaei H., Mansournia M.A., Asemi Z. (2020). Effects of whey protein on glycemic control and serum lipoproteins in patients with metabolic syndrome and related conditions: A systematic review and meta-analysis of randomized controlled clinical trials. Lipids Health Dis..

[428] Adams R.L., Broughton K.S. (2016). Insulinotropic Effects of Whey: Mechanisms of Action, Recent Clinical Trials, and Clinical Applications. Ann. Nutr. Metab..

